# Transcriptional and morphological responses following distinct muscle contraction protocols for Snell dwarf (*Pit1^dw/dw^*) mice

**DOI:** 10.14814/phy2.70027

**Published:** 2024-09-03

**Authors:** Erik P. Rader, Kimberly A. McKinstry, Brent A. Baker

**Affiliations:** ^1^ Centers for Disease Control and Prevention National Institute for Occupational Safety and Health Morgantown West Virginia USA

**Keywords:** plantarflexor muscles, skeletal muscle, stretch‐shortening contractions

## Abstract

The Snell dwarf mouse (*Pit1* ^*dw/dw*^), an animal model of congenital combined pituitary hormone deficiency, displays skeletal muscle weakness. While enhanced responsivity to repeated exposures of muscle contractions have been documented for Snell dwarf mice, the response following single exposure to distinct contraction protocols remained uncharacterized. The purpose of this study was to investigate the muscle recovery of Snell dwarf and control littermate mice following a single exposure to two separate protocols—an intermittent slow velocity (30°/s) contraction protocol or a continuous rapid velocity (500°/s) contraction protocol. Following both protocols for control mice, torque values were 30% and 80% of pre‐protocol values at 5 min and 3 days, respectively. At 10 days, performance returned to baseline for the 30°/s protocol and were depressed for the 500°/s protocol. For Snell dwarf mice following both protocols, torques were depressed to 5% of pre‐protocol values at 5 min and returned to baseline by 3 days. Recovery following the 30°/s protocol for control mice and both protocols for Snell dwarf mice coincided with increased transcriptional output, upregulation of cytokine‐mediated signaling genes, and a distribution shift to smaller muscle fibers with reduced area per nucleus. These features represent efficacious remodeling ubiquitous across distinct contraction paradigms in the context of the *Pit1* mutation.

## 1 INTRODUCTION

The Snell dwarf mouse, a mouse model first described almost a century ago, represents the first case of hereditary dwarfism noted in mice and has proven to be valuable in basic research regarding congenital combined pituitary hormone deficiency and longevity (Bartke, 2021; Qian et al., 2022; Snell, 1929). Congenital combined pituitary hormone deficiency with deficiencies in multiple pituitary hormones has an incidence up to 1 per 10,000 births in the human population (Jakobsen et al., 2023). Snell dwarf mice harbor a spontaneous recessive point mutation in *Pit1*(*Pou1f1*), an anterior pituitary transcriptional factor, resulting in growth hormone, prolactin, and thyroid‐stimulating hormone deficiencies (Qian et al., 2022). With more than 30 pathogenic variants of *PIT1* reported, these mutations are among the most common of known genetic causes for combined pituitary hormone deficiency (Akiba et al., 2022; Chen et al., 2021; Hassan et al., 2022). Consequences of congenital hypopituitarism are characterized by blunted development and short stature (Jakobsen et al., 2023; Qian et al., 2022).

Given the growth hormone deficiency and reduction of insulin‐like growth factor‐1 to near undetectable levels, compromised skeletal muscle size and, consequently, strength would be expected with the *Pit1* mutation and valuable to characterize (Brown‐Borg & Bartke, 2012; Cuneo & Wallace, 2005; Huuskonen et al., 2011). However, despite multiple lines of research regarding longevity investigated in mutant dwarf mouse models such as Snell dwarf mice, reports relevant to skeletal muscle are limited. For instance, a study regarding biceps brachii and soleus muscles of 33 day old Snell dwarf mice reported muscle mass and length values consistent with muscle girth estimates that were 35% of those in wild‐type mice (Stickland et al., 1994). Meanwhile, investigations of long‐lived Ames dwarf mutant (*Prop1* ^*df/df*^) mice (mice also deficient in growth hormone, prolactin, and thyroid‐stimulating hormone) assessed lean body mass and exposed mice to physical activity such as swimming and or the wire hang test (Arum et al., 2013; Heiman et al., 2003; Romanick et al., 2004). However, direct measures of skeletal muscle performance were undocumented especially in the context of the *Pit1* mutation. In this setting, our laboratory sought to characterize these features for the Snell dwarf mouse.

Utilizing a dynamometer to assess plantarflexor muscles in vivo, we determined that maximum isometric torques of Snell dwarf mice (3‐ to 12‐month‐old) were only 10% of their wild‐type littermate values (Rader et al., 2022). Meanwhile, plantarflexor muscle mass normalized to tibial length for Snell dwarf mice was 30% of those for wild‐type littermates indicating that muscles were weak with the *Pit1* mutation even after accounting for smaller muscle size. The mice were then exposed to repeated sessions (three sessions per week for 4 weeks) of stretch‐shortening contractions (SSCs), contractions common when lifting loads, in which muscle is activated throughout a sequence of three consecutive phases—muscle at fixed length (isometric), stretched, and lastly, shortened (Heckel et al., 2019; Rader & Baker, 2017; Vaczi et al., 2014). These SSCs were administered intermittently (3 s pauses between each SSC) and at a slow velocity (30°/s ankle rotation), parameters comparable to those undertaken by individuals when lifting loads (Heckel et al., 2019; Vaczi et al., 2014). Snell dwarf mice exhibited exceptional adaptation after the repeated SSC exposures (Rader et al., 2022). For instance, muscles of Snell dwarf mice became more resistant to initial performance deficits 5 min following SSCs—evident by a threefold increase in post‐session torque (final session value vs. first session value) while wild‐type littermates increased their values by 1.5‐fold. In addition, Snell dwarf mice were less susceptible to reductions in maximum isometric torque from session to session of SSC exposures. While muscles of adult wild‐type littermates maladapted becoming weaker during 4 weeks of SSC exposures, no such performance drop was observed for age‐matched Snell dwarf mice. Furthermore, evidence of remodeling was observed for muscles of Snell dwarf mice in the form of increased muscle fiber density—a feature absent in the littermate response. Overall, these findings demonstrated an extraordinary capacity for recovery to SSCs and a resistance to maladaptation for muscles of Snell dwarf mice.

While this research provided insights on intermittent slow velocity SSCs, several issues were unresolved. Individuals are also exposed to isolated instances (i.e., single exposures) of SSCs (i.e., not always regularly repeated). Consequently, evaluating muscle multiple days following such a single exposure becomes relevant. Furthermore, SSCs vary in velocity (up to several hundreds of degrees per second for plyometric‐type movements) and muscle activation mode (intermittent vs. continuous activation) (Bobbert et al., 1987; Mori et al., 2014; Rader & Baker, 2017; Vaczi et al., 2011, 2014). The topic of contraction velocity has been debated especially in the context of plyometric versus traditional resistance exercise in terms of anabolic signaling. While a growing body of evidence has indicated that such signaling can occur in plyometrics, the response is better established in traditional resistance activity (Arntz et al., 2022; Grgic et al., 2021). This has been attributed to the stimuli of greater time under tension that accompany slow velocity contractions in resistance exercise (Burd et al., 2012; Chapman et al., 2006; Grgic et al., 2021; Wilk et al., 2021). Meanwhile, susceptibility to compromised adaptation has been demonstrated following rapid velocity contractions especially during stretching (i.e., lengthening contractions) resulting in high peak tension and possibility of muscle fiber ultrastructural damage (Chapman et al., 2006; McCully & Faulkner, 1986; Mori et al., 2014; Rader & Baker, 2017). Aside from research gaps regarding single exposures and different SSCs in the context of the *Pit1* mutation, outcomes of cytokine‐mediated signaling gene expression, total RNA levels, and myonuclei morphology are lacking. Cytokine‐mediated signaling has a significant role in chemotaxis and remodeling following contractions (Docherty et al., 2022; Ochi et al., 2011; Rader et al., 2015). Increased total RNA is indicative of global transcriptional output and ribosomal biogenesis, factors in adaptation (Figueiredo & McCarthy, 2019; Kotani et al., 2019, 2021; Rader & Baker, 2022). Meanwhile, morphological features such as increased muscle fiber density and decreased muscle fiber area per myonucleus (indicative of decreased myonuclear domain) has been proposed to enhance transcriptional capacity per unit of tissue (Dungan et al., 2019; Murach et al., 2022; Rader & Baker, 2022).

The purpose of this study was to characterize these features and assess recovery for muscles of Snell dwarf and control littermate mice in the days following a single exposure to two distinct SSC protocols—an intermittent slow velocity (30°/s ankle rotation) contraction protocol or a continuous rapid velocity (500°/s) contraction protocol. Given the exceptional capacity for the remodeling and adaptative responses to repeated SSC exposures observed previously for Snell dwarf mice (relative to the lack of such responses for control mice), the proposal emerged that muscles of Snell dwarf mice may be resistant to maladaptation from SSCs in general and, consequently, less susceptible to divergent responses. Conversely, muscles of wild‐type littermate mice may be more sensitive to differences in SSC exposures. Therefore, the following hypotheses were tested—that for control mice, transcriptional/morphological responses and muscle mass/performance recovery would be disparate between the two protocols (with greater responses/recovery following the 30°/s protocol vs. 500°/s protocol) while for Snell dwarf mice, such responses and recovery would be more ubiquitous across both protocols. The outcomes of this research have implications for research regarding what physical activities to consider for improving the skeletal muscle phenotype and quality of life in this form of congenital hypopituitarism.

## 2 MATERIALS AND METHODS

### 2.1 Experimental animals

Male Snell dwarf (*Pit1* ^*dw/dw*^) mice and their age‐matched normal‐phenotype control (*Pit1* ^*dw/+*^ and *Pit1* ^*+/+*^) littermates were F1 generation produced by bidirectional mating of DW/J *Pit1* ^*dw/+*^ (Jax# 000643) and B6.DW *Pit1* ^*dw/+*^ (Jax# 000772) mice (Madsen et al., 2004; Rader et al., 2022). The mice were provided NIH‐31 Open diet (Teklad 7917, Indianapolis, IN, USA) ad libitum and housed in an Association for Assessment and Accreditation of Laboratory Animal Care International—accredited animal quarters. Two normal‐sized female littermate was housed with each Snell dwarf mouse—that is, three mice per cage regarding Snell dwarf (*Pit1* ^*dw/dw*^) mice. Housing with at least one normal‐sized littermate is needed to provide warmth to the Snell dwarf mouse (Flurkey et al., 2001, 2002; Madsen et al., 2004). Each male control mouse was housed singly—that is, one mouse per cage regarding control (*Pit1* ^*dw/+*^ and *Pit1* ^*+/+*^) mice. At 3 months of age, mice were randomized, anesthetized (isoflurane gas 2%–3% by volume), and muscles were exposed to either an intermittent slow velocity contraction protocol or continuous high velocity contraction protocol (Figure S1). The muscles of the mice were assessed again at either 3 days—a time period in which inflammatory and degenerative responses can be observed—or 10 days—a time period in which degree of recovery can be assessed (Baker et al., 2006; Brooks & Faulkner, 1990; McCully & Faulkner, 1985; Pizza et al., 2002; Rader et al., 2015). After this assessment while still anesthetized, all mice were euthanized by pentobarbital (100–300 mg/kg body weight) intraperitoneal injection followed by exsanguination. All animal procedures were done in accordance with the Guide for the Care and Use of Laboratory Animals (8th edition, National Academies Press) and approved by the Animal Care and Use Committee at the National Institute for Occupational Safety and Health in Morgantown, WV.

### 2.2 SSC exposures

The SSC exposures were based on previous protocols in our laboratory for intermittent slow velocity contractions and continuous high velocity contractions in rats and mice (Rader et al., 2017, 2022; Rader & Baker, 2020). Each mouse was anesthetized with isoflurane gas (2%–3% by volume), placed in dorsal recumbency on a heated table, and the left foot secured to a footplate of a dual mode muscle lever system (Whole Mouse Test System, 1300A, Aurora Scientific, ON, Canada). Platinum electrodes were placed subcutaneously to activate the tibial nerve and stimulation parameters were set for maximal contraction at 8‐V magnitude, 0.2‐ms pulse duration, and 150‐Hz frequency. Prior to protocol exposure, static and dynamic plantarflexion performances were evaluated (Figure S2). Static performance consisted of a maximal isometric contraction with the ankle at a 90° angle between tibia and foot (Ashton‐Miller et al., 1992). After a 1 min rest period, dynamic performance was first assessed by a slow SSC (SSC_s_) consisting of an isometric contraction for 100 ms at 90° ankle angle immediately followed by rotation to 70° at 30°/s (i.e., the lengthening contraction phase), returning to 90° at the same velocity (i.e., the shortening contraction phase), and then immediately holding at that angle for an additional 100 ms isometric contraction—with plantarflexor muscles activated during the entire duration of the different phases of the SSC_s_. The second dynamic assessment consisted of a fast SSC (SSC_f_) which was the same as SSC_s_ with the exception that velocity was 500°/s. Peak torque values were recorded for these dynamic contractions (and all SSCs of the study) at the apex of the lengthening contraction portion of SSCs.

Both SSC exposure protocols consisted of 8 sets with 2 min rest intervals between sets and 10 SSCs per set (Figure S2). Each SSC of the intermittent slow velocity contraction protocol (30°/s protocol) were separated by 3 s and involved the same contraction parameters as SSC_s_. The SSCs for the continuous high velocity contraction protocol were the same as those the 30°/s protocol with the exceptions the contractions were continuous (i.e., no rest periods between each SSC) within each set and at 500°/s. The torque time integral was calculated as the integral of torque versus time during the stretch and shortening phases of each SSC (Jakobsgaard et al., 2021). Work was determined by dividing torque time integral by the time interval to calculate average torque and then multiplying by the angular displacement (Mendias et al., 2006). Power was calculated by dividing work by the time interval (i.e., rate of work) (Lynch et al., 2001). Set total values for torque time integral, work, and power were determined by summing the values of all the SSCs in the set. To determine the effect of SSC protocol on acute performance, isometric contraction, SSC_s_, and SSC_f_ measures were recorded at 5, 6, and 7 min post‐protocol, respectively, at the same settings as pre‐protocol measures. At 3 or 10 days, static and dynamic performance was assessed again using the same settings as those for pre‐protocol measures. These time points corresponded with periods of cytokine response (at 3 days) in muscles of rats exposed to SSCs and significant recovery of performance and muscle fiber morphology following SSCs (at 10 days) (Baker et al., 2006; Krajnak et al., 2006; Rader et al., 2015). Immediately following this assessment, both right and left plantarflexor muscles were removed and weighed. Tibia length was measured and used to normalize muscle mass (muscle mass divided by tibia length). Baseline muscle quality was calculated by dividing initial maximal isometric torque by normalized muscle mass of the nonexposed plantarflexor muscle group. Each gastrocnemius muscle mid‐belly portion was immersed in tissue freezing media and placed in cold isopentane (−160°C) for quantitative morphology/immunofluorescence while remaining tissue was stored for RNA and DNA analysis.

### 2.3 Quantitative morphology

Gastrocnemius muscle tissue was cryosectioned (12 μm thickness), hematoxylin and eosin stained, and analyzed by a stereological procedure as done previously and described below (Baker et al., 2006; Rader et al., 2016, 2018). The stereological method in biomedical research was established more than 50 years ago and has been utilized in various analyses of a variety of tissues including skeletal muscle (Broskey et al., 2013; Darban Maghami et al., 2022; Mandarim‐de‐Lacerda, 2003; Weibel et al., 1966). The investigator was blinded to sample identification. At each microscope field (40× magnification), nodes (i.e., line intersections) of a 121‐node 11‐line overlay graticule (0.04 mm^2^ square with 100 divisions) were evaluated. Each node was identified as overlaying a degenerative muscle fiber, nondegenerative muscle fiber (further classified as peripherally nucleated or centrally nucleated), or interstitium (cellular or noncellular). Degenerative muscle fibers were defined as having the following attributes—loss of contact with surrounding fibers, interdigitation of the sarcolemma by cellular infiltrates, and internalization of cellular infiltrates (Baker et al., 2006). A node was designated as cellular interstitium when overlaying a nucleus between muscle fibers whereas a node classified as noncellular interstitium overlayed a point between muscle fibers without a nucleus. A total of 10 fields (or maximum number of fields possible without overlap) were assessed per muscle section—5 fields for both lateral and medial regions of the muscle section (so that up to 1210 nodes were evaluated per muscle). Percent of muscle tissue was calculated as the percentage of nodes in the sampled cross‐section which overlaid each type of tissue relative to the total number of nodes.

### 2.4 Total RNA and mRNA analysis

A portion of frozen gastrocnemius (GTN) muscle tissue was homogenized with a Mini‐BeadBeater 8 (Biospec) while in a vial of 1 mL of TRIzol with 1.0 mm zirconia beads (BioSpec, Cat#11079110zx, Bartlesville, OK, USA). Due to the small muscle sizes of the Snell dwarf mice, tissue availability was limited resulting in the tissue amount analyzed varying from that of control mice (~17 mg for Snell dwarf mice and ~23 mg for control mice). Therefore, data analysis regarding RNA was performed for each genotype separately and with tissue amount as a random factor to account for any possible effect of variations in starting material. Total RNA was isolated using a RNAqueous phenol‐free total RNA Isolation Kit (Ambion, Cat# AM1912, USA) and concentration quantified (NanoDrop 2000c, Thermo Fisher Scientific, Pittsburgh, PA, USA) (Rader & Baker, 2022). The cDNA was synthesized using the RT^2^ First Strand Kit (Qiagen, Cat# 330401, USA). The expression of genes relevant to cytokine‐mediated signaling was analyzed per manufacturer's instructions for the RT^2^ Profiler™ PCR Array (Qiagen, Cat# PAMM‐077Z, USA). Gene expression was considered significantly differentially regulated when fold regulation exceeded twofold regulation (below 0.5‐fold change or above 2.0‐fold change) and *p* < 0.05.

### 2.5 DNA methylation

Total DNA was isolated from *~*15 mg of frozen GTN muscle using the DNeasy Blood & Tissue Kit (Qiagen, Cat# 69506, USA) and gene promoter DNA methylation was determined by means of a cytokine‐relevant EpiTect Methyl II PCR Array (Qiagen, Cat# EAMM‐521ZA, USA) per manufacturer's instructions (Rader & Baker, 2020). This system consists of PCR quantification of remaining DNA following either digestion of unmethylated DNA, methylated DNA, both unmethylated and methylated DNA, or a mock digestion (i.e., no enzymes included) of DNA. A quantity of 1 μg of genomic DNA was added to restriction digestion buffer (120 μL total volume). This was then apportioned to the four different restriction digest conditions. A PCR master mix was added to each digest and then loaded (25 μL) into each well of the array. Each well contained primers flanking a distinct promoter region of interest. The PCR was then run using an Applied Biosystems 7500 Real‐Time PCR instrument. Percent methylation was calculated from C_t_ values analyzed with a template provided by Qiagen.

### 2.6 Immunofluorescence

GTN muscle sections were fixed in HistoChoice (Sigma‐Aldrich; H2904, Hatfield, PA, USA) for 45 min, washed (3 × 5 min in PBS), permeabilized (0.2% Tween20 in PBS for 10 min), washed (3 × 5 min in PBS), and then blocked with 10% donkey serum in PBST (0.05% Tween20 in PBS) for 1 h. A primary polyclonal antibody for dystrophin (Abcam; ab15277; 1:400) was applied for 1 h, sections were washed (3 × 5 min in PBS), and secondary antibody (donkey anti‐rabbit IgG Cy3; Millipore Sigma AP182CMI, USA; at 1:500 in PBST) was applied for 2 h. Sections were washed (3 × 5 min washes in PBS) and mounted with Prolong™ Gold Antifade Reagent (Thermo Fisher Scientific; P36931, USA) with 4′, 6‐diamidino‐2‐phenylindole (DAPI). With the investigator blinded to sample identification, each muscle section was assessed based on a standardized method (Rader & Baker, 2022). For both lateral and medial regions of the muscle section, non‐overlapping images were captured and an overlay graticule with a 0.04 mm^2^ square boundary was placed at the center of each image. Using ImageJ (version 1.46, National Institutes of Health, USA), muscle fibers (and their myonuclei) in which the topmost portion was within the graticule boundary were analyzed. For each section, muscle fibers (144 ± 87 fibers per section, 8795 fibers for entire study) and myonuclei (179 ± 84 nuclei per section, 10,929 nuclei for entire study) were manually counted and traced to determine muscle fiber size, nuclei size, nuclei shape, and fiber area per nucleus. Furthermore, the index of circularity was determined for each nucleus by the equation 4π (area/perimeter^2^) with a perfect circle as a value of 1 and increasing elongation as the value decreases.

### 2.7 Statistical analysis

Data were analyzed using ANOVA (JMP version 15, SAS Institute, Inc., Cary, NC, USA) with the variable of animal identification as a random factor to account for repeated measures when appropriate. Post hoc comparisons were performed using Fisher's least significant difference method. Data regarding degenerating muscle fibers were not normally distributed and, therefore, analyzed by Kruskal–Wallis ANOVA on ranks. Differences in frequency distribution were assessed by chi‐squared analysis (SigmaPlot version 14.0, Chicago, IL, USA). All data are expressed as means ± standard deviation (SD). *p* < 0.05 was considered statistically significant.

## 3 RESULTS

### 3.1 At baseline, muscles of Snell dwarf mice were small and weak even after accounting for tibial length

Body weights for Snell dwarf mice (8.1 ± 1.0 g) were 25% of those for control mice (32.1 ± 4.3 g), *p* < 0.0001. Meanwhile, tibial lengths for Snell dwarf mice (11.9 ± 0.4 mm) were 65% of those for control mice (18.2 ± 0.3 mm), *p* < 0.0001. At baseline, muscles of Snell dwarf mice were small and weak. Snell dwarf values of muscle mass normalized to tibial length of gastrocnemius, plantaris, and soleus were 35%, 33%, and 37%, respectively, of values for control mice, *p* < 0.05 (Table S1). Initial maximum isometric torque, SSC_s_ peak torque, and SSC_f_ peak torque values of Snell dwarf mice were 14%, 18%, and 18% compared with control mice (Figure S3). Muscle weakness was evident after accounting for the difference in muscle size by dividing isometric performance by normalized muscle mass to determine muscle quality. Snell dwarf muscle quality was 40% relative to that of control mice (0.58 ± 0.16 mN·m/mg/mm vs. 1.45 ± 0.18 mN·m/mg/mm, *p* < 0.0001).

### 3.2 During both SSC protocols, distinct performance profiles resulted in comparable torque deficits. These deficits were more pronounced for Snell dwarf mice

The performance profiles during the two protocols were distinct—greater torque, work, and power values were characteristic of the 500°/s protocol and prolonged time under tension and greater torque time integral values were typical of the 30°/s protocol. For both genotypes, the initial SSC of the first set for the 500°/s protocol elicited a 1.5‐fold greater value of peak torque compared with that of the 30°/s protocol value (Table S2). Furthermore, the greater peak torques of the 500°/s protocol (relative to the 30°/s protocol) persisted throughout the first set. For instance, for control mice, by the last contraction of the first set for the 500°/s protocol, peak torque (19.93 ± 7.01 mN·m) was 85% of that of the initial SSC (23.90 ± 8.03 mN·m, *p* < 0.0001). In contrast, by the end of the first set for the 30°/s protocol for control mice, SSC peak torque (5.20 ± 2.11 mN·m) dropped to 35% of that of the initial SSC (15.62 ± 4.25 mN·m) *p* < 0.0001. Considering the first set for both genotypes, total work and power were twofold, and 40‐fold greater, respectively, for the 500°/s protocol relative to 30°/s protocol values (Table S2). However, compared with the 500°/s protocol, the 30°/s protocol‐induced sevenfold greater torque time integral values for both genotypes (Table S2). This was consistent with a 17‐fold longer time under tension inherent in design of the 30°/s protocol (vs. the 500°/s protocol), 13.3 s versus 0.8 s of total time of active stretch‐shortening during each set.

The performance decrements by the end of the protocols were influenced by genotype. By the last set for both protocols, total work, power, and torque time integral dropped to a greater extent for Snell dwarf mice relative to that of control mice—to 30% of first set values for Snell dwarf mice versus 40% of first set values for control mice, *p* < 0.05 (Table S2). For control mice exposed to the 30°/s protocol, short‐term fatigue was observed. This was evident since a 1/3rd of the torque deficit for the last SSC was transient with peak torque increasing from 3.98 ± 1.63 mN·m for the last SSC of the final set to 7.18 ± 4.10 mN·m for the comparable SSC (i.e., SSC_s_) at 6 min afterwards. For the 500°/s protocol to control mice, the torque deficit was more sustained with peak torque at 10.17 ± 8.21 mN·m by the last SSC with no increase 7 min post‐protocol for the comparable SSC (i.e., SSC_f_, *p* = 0.432). A sustained torque deficit in the minutes following the SSC protocols was also observed for Snell dwarf mice as evident by no increase in SSC peak torque following both protocols relative to final protocol SSC values (*p* > 0.05). Regarding the torque deficits for control mice which remained minutes following both protocols, maximum isometric torques at 5 min, SSC_s_ peak torques at 6 min, and SSC_f_ peak torques at 7 min were 30%, 40%, and 40% of pre‐protocol values, respectively (Figure S3). Muscles of Snell dwarf mice exhibited greater performance reductions post‐protocols with maximum isometric torques at 5 min, SSC_s_ peak torques at 6 min, and SSC_f_ peak torques at 7 min that were 5%, 30%, and 30% of pre‐protocol values (Figure S3). A significant interaction between genotype and timing (pre‐ vs. post‐protocol) was observed for these measures (*p* < 0.0001).

### 3.3 For control mice, performance and muscle mass outcomes were dependent on SSC protocol whereas for Snell dwarf mice, these outcomes were protocol‐independent

**FIGURE 1:**
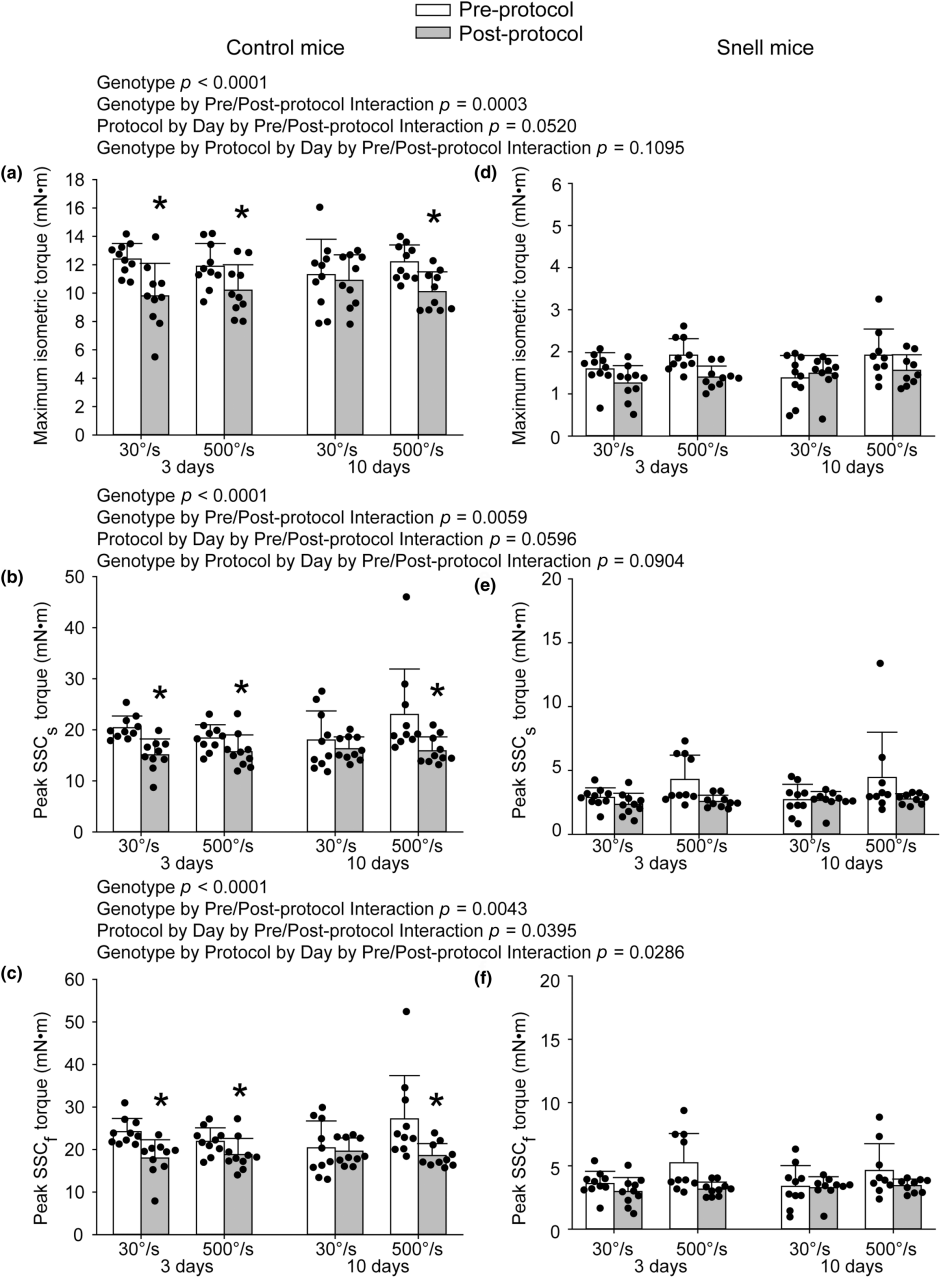
For control mice, significant deficits in maximal performance values were observed at 3 days following both the 30°/s and 500°/s protocols whereas at 10 days only the 500°/s protocol exhibited a deficit. Post‐ versus pre‐protocol comparisons for Snell dwarf mice did not reach significance. Performance included maximum isometric torque (a), SSC_s_ peak torque (b), and SSC_f_ peak torque (c) for control mice and maximum isometric torque (d), SSC_s_ peak torque (e), and SSC_f_ peak torque (f) for Snell dwarf mice. Sample sizes were *N* = 9 to 10 per group. Dots represent raw values. Error bars denote means ± SD. Relevant ANOVA interactions are noted. *Different from pre‐protocol value, *p* < 0.05.

Performance measures at 3 days were depressed to 80% of pre‐protocol values for muscles of control mice regardless of protocol (Figure 1a–c). However, by 10 days protocol‐dependent results emerged. Performance returned to pre‐protocol values following the 30°/s protocol whereas the deficit remained following the 500°/s protocol (Figure 1a–c). The outcomes for muscles of Snell dwarf mice were distinct in that performance measures were at approximate baseline values already by 3 days following both protocols (Figure 1d–f). Overall, the factors of number of days post‐protocol and genotype on performance are demonstrated by the ANOVA interaction of genotype, protocol, and pre/post‐protocol timing for SSC_f_ peak torque, *p* = 0.0286 (Figure 1c,f).

**FIGURE 2:**
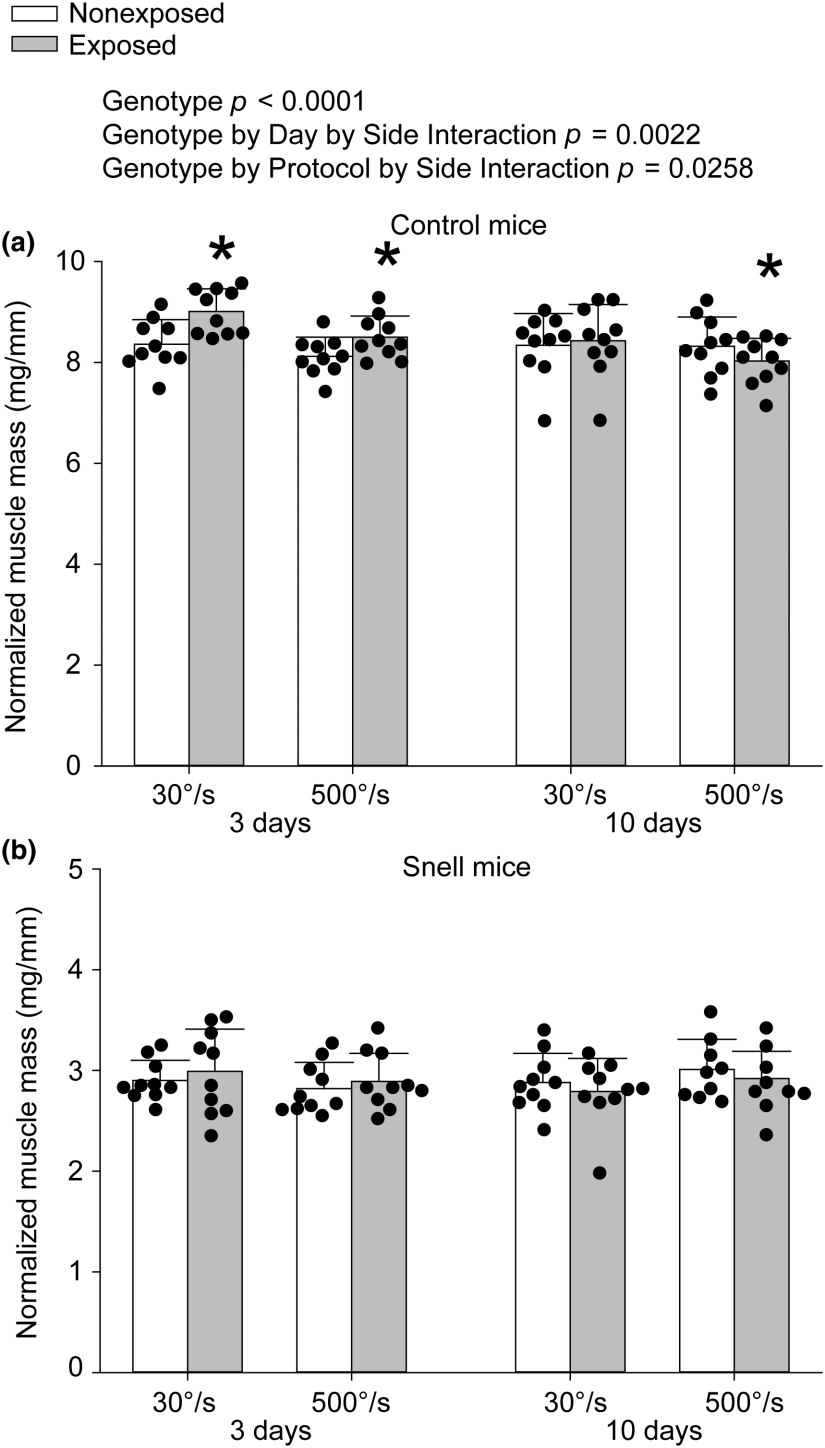
For control mice (a), both protocols increased plantarflexor muscle mass at 3 days while at 10 days muscle mass was decreased only following the 500°/s protocol. For Snell dwarf mice (b), although responses followed a similar profile to control, exposed versus nonexposed differences in muscle mass did not reach significance. Sample sizes were *N* = 9 to 10 per group. Dots represent raw values. Error bars denote means ± SD. Relevant ANOVA interactions are noted. *Different from nonexposed value, *p* < 0.05.

For control mice, both the 30°/s and 500°/s protocols induced plantarflexor muscle mass increases of approximately 5% at 3 days indicative of edema, *p* < 0.0001 for the 30°/s protocol and *p* = 0.0002 for the 500°/s protocol (Figure 2a). While muscle mass returned to baseline levels for the 30°/s protocol, muscle mass was 5% lower in the exposed leg for the 500°/s protocol at 10 days, *p* = 0.0044 (Figure 2a). No protocol‐induced changes in muscle mass were observed for Snell dwarf mice at 3 and 10 days (Figure 2b). The ANOVA interaction of genotype with number of days post‐protocol, protocol (500°/s vs. 30°/s), and exposure status (i.e., left exposed vs. right nonexposed muscle) was significant at *p* = 0.0258.

### 3.4 Transient increased interstitium 3 days after the 30°/s protocol for control mice and both protocols for Snell dwarf mice

To characterize the muscle tissue in the days following the protocols, noncellular interstitium, cellular interstitium, degenerative muscle fibers, nondegenerative muscle fibers, and nondegenerative centrally nucleated muscle fibers were quantified (Figure S4). For control mice, the tissue percentage of noncellular interstitium increased 3 days following the 30°/s protocol, the same time period of increased muscle mass for that group (Figure S5a). This was not accompanied by an increase in cellular interstitium or overt degenerative muscle fibers (Figure S5b,c). A decrease in tissue percentage of peripherally nucleated muscle fibers was observed at 3 days concomitant with the increased noncellular interstitium tissue percentage at that time (Figure 5d). Peripherally nucleated muscle fiber tissue percentage was also decreased 10 days post 30°/s protocol which coincided with the increase in centrally nucleated muscle fiber tissue percentage, an indicator of remodeling, for the same group (Figure S5e).

For muscles of Snell dwarf mice, noncellular interstitium tissue percentage was elevated for both protocols at 3 days (Figure S5f). Cellular interstitium was greater in general for muscles of Snell dwarf mice but no protocol‐induced changes were observed (Figure S5g). The tissue percentage of degenerative muscle fibers was <1% of the tissue (Figure S5h). Similar to control mice, a decrease in peripherally nucleated muscle fiber tissue percentage at 10 days following the 30°/s protocol reflected an increase in centrally nucleated muscle fiber tissue percentage (Figure S5j).

### 3.5 For control mice transcriptional output was protocol dependent whereas transcriptional responses for Snell dwarf mice were comparable between protocols

**FIGURE 3:**
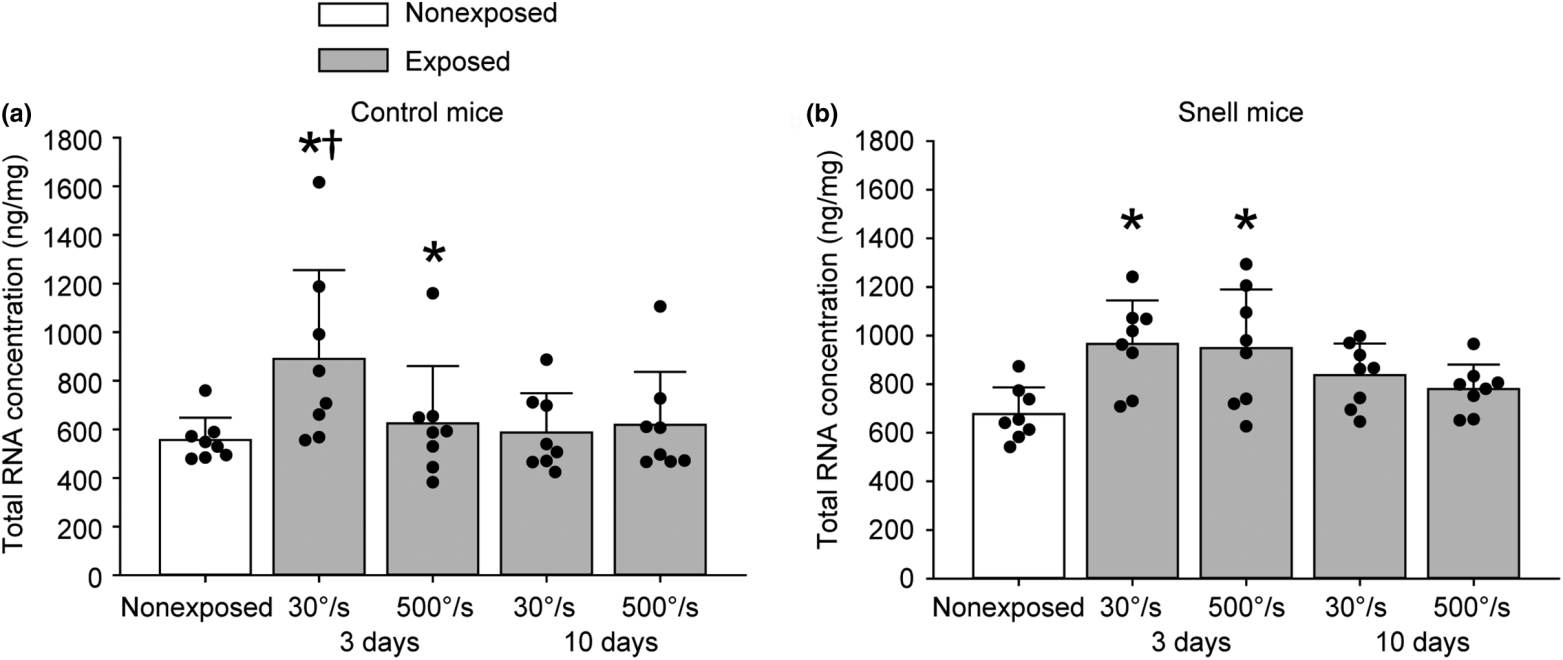
For control mice (a), total RNA concentration was elevated 3 days following both 30°/s and 500°/s protocols with a greater extent following the 30°/s protocol. For Snell dwarf mice (b), the elevations at 3 days were independent of protocol. Sample sizes were *N* = 8 per group. Dots represent raw values. Error bars denote means ± SD. *Different from nonexposed value, *p* < 0.05. †Different from time‐matched 500°/s protocol value, *p* < 0.05.

For muscles of control mice at 3 days, the 30°/s protocol induced a 1.6‐fold increase in total RNA concentration while the 500°/s protocol induced increased total RNA levels but to a diminished degree (Figure 3a). For muscles of Snell dwarf mice, total RNA concentration was elevated by 1.4‐fold for both protocols at 3 days (Figure 3b). These greater total RNA responses coincided with favorable performance and muscle mass outcomes.

### 3.6 Magnified upregulation of cytokine‐mediated signaling genes 3 days following the 30°/s protocol for control mice and both protocols for Snell dwarf mice

**FIGURE 4:**
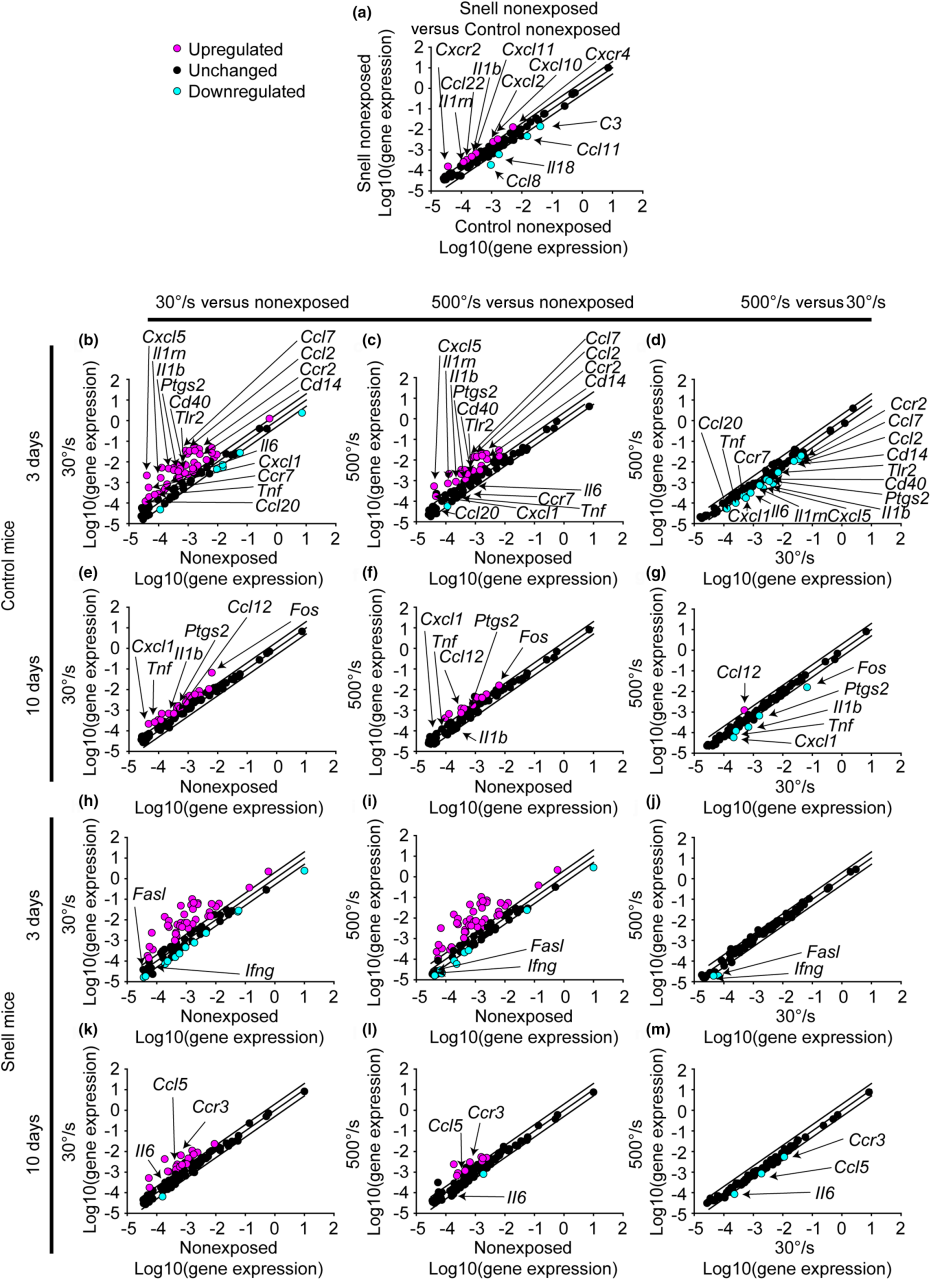
Each scatter regarding cytokine‐mediated signaling genes compares the gene expression (2^ (‐Delta C_T_)) between two selected groups—(a) nonexposed muscles of Snell dwarf vs control mice and (b–m) contraction‐exposed vs nonexposed muscles or between contraction protocols for control and Snell dwarf mice. Sample sizes were *N* = seven to nine per group. The center diagonal line indicates unchanged gene expression while the outer lines represent the twofold threshold for up or downregulation. Genes were considered differentially expressed when fold regulation exceeded twofold regulation and *p* < 0.05. Genes which were differentially regulated when comparing 500°/s versus 30°/s exposure were identified on the scatter plot and additionally denoted in the corresponding exposed vs nonexposed plots for reference.

To investigate gene expression relevant to remodeling, mRNA levels of 88 genes—with an emphasis on cytokine‐mediated signaling—were analyzed (Tables S3–, S15 and Figure 4a–m). Genes were considered differentially expressed when fold regulation exceeded 2‐fold regulation and *p* < 0.05. For muscles of control mice, the 30°/s protocol at 3 days induced 44 upregulated genes and 8 downregulated genes (Figure 4b). Meanwhile, the 500°/s protocol resulted in 24 upregulated genes and 1 downregulated gene compared to nonexposed muscle (Figure 4c). To assess how gene expression differed between the protocols directly, muscles exposed to the 500°/s protocol were compared relative to muscles exposed to the 30°/s protocol. At 3 days, gene expression for 15 genes were lower for the 500°/s protocol vs that of the 30°/s protocol (Figure 4d). At 10 days, gene expression for the 500°/s protocol was also less overall relative to the 30°/s protocol as evident by 5 genes with lower expression (Figure 4g).

At baseline, muscles of Snell dwarf mice differentially expressed 12 genes for chemokines, interleukins, and their receptors relative to those of control mice—an outcome consistent with an increased tissue percentage of cellular interstitium (Figure 4a). The 30°/s protocol at 3 days induced 37 upregulated genes and 12 downregulated genes (Figure 4h). The response 3 days following the 500°/s protocol was comparable with 43 upregulated genes and 8 downregulated genes (Figure 4i). The responses following both protocols were so alike that only two genes were differentially regulated when directly comparing them with each other (Figure 4j). At 10 days, less genes were upregulated for both protocols relative to that at 3 days (Figure 4k,l).

Venn diagrams demonstrated that while considerable differential gene expression was in common among the protocols for control mice (25 genes at 3 days and 11 genes at 10 days), additional gene regulation was apparent following the 30°/s protocol (25 genes at 3 days and 10 genes at 10 days) (Figure S6a,b). For instance, upregulation of *Actb*, encoding a key cytoskeleton protein, was exclusively observed after the 30°/s protocol (Figure S6a). For Snell dwarf mice, almost all the differentially regulated genes were in common for both protocols. For example, *Actb* was upregulated following both protocols in muscles of Snell dwarf mice (Figure S6a).

To determine whether DNA methylation of cytokine‐relevant genes was a possible underlying process following the distinct protocols, additional tissue exclusively available for control mice was analyzed. Alterations in DNA methylation were not pervasive—of the 18 genes analyzed, only one (*Tgfb1*) demonstrated demethylation of promoter DNA and this occurred for the group corresponding with 3 days post 30°/s protocol (Figure S7).

### 3.7 The 30°/s protocol induced a shift to smaller fibers with reduced fiber area per nucleus in for control mice whereas both protocols induced these outcomes for Snell dwarf mice

**FIGURE 5:**
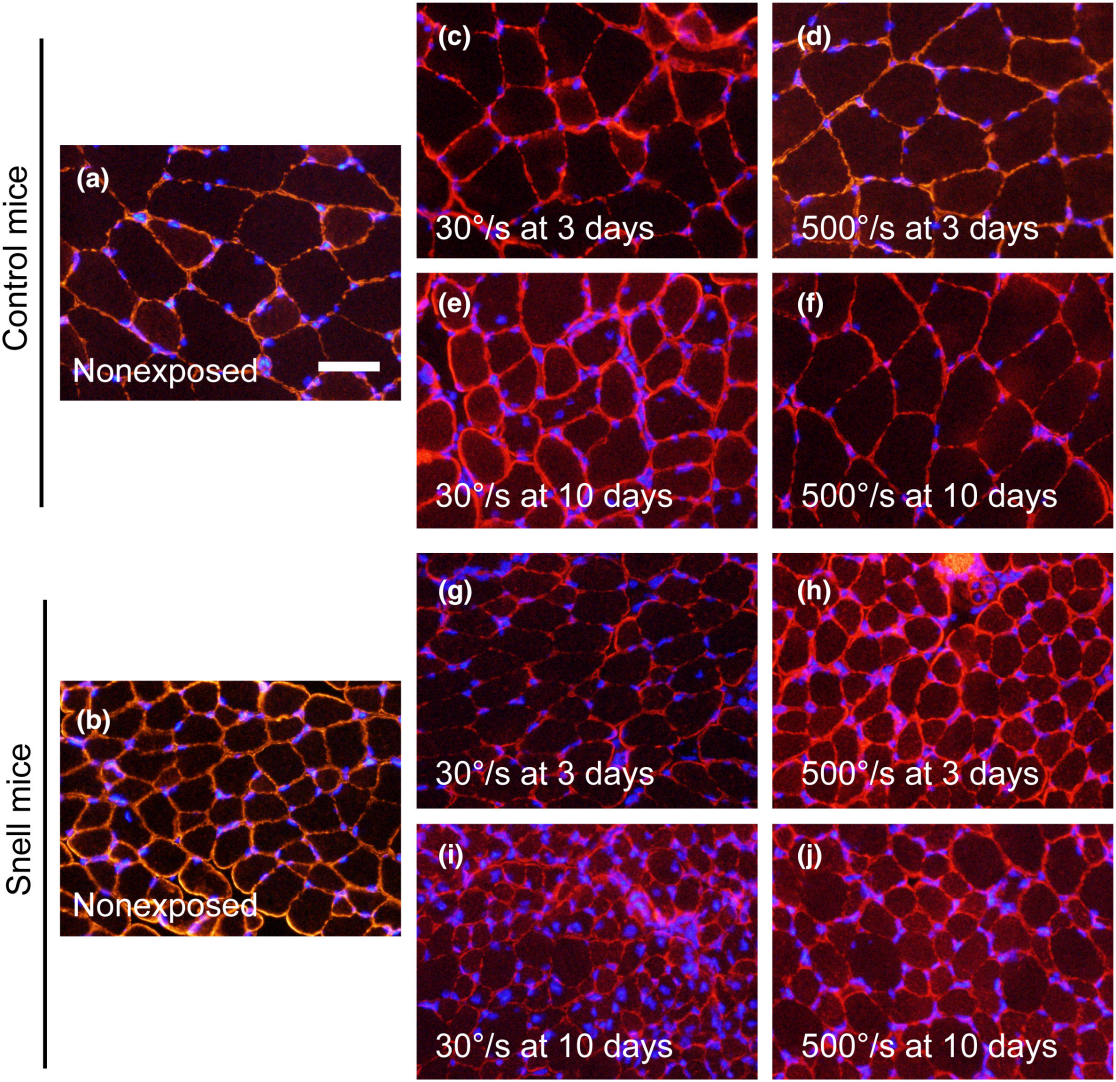
Immunofluorescence labeling for dystrophin (red) and nuclei (blue) in transverse sections of nonexposed (a, b) and exposed (c–j) muscles. Scale bar = 50 μm.

**FIGURE 6:**
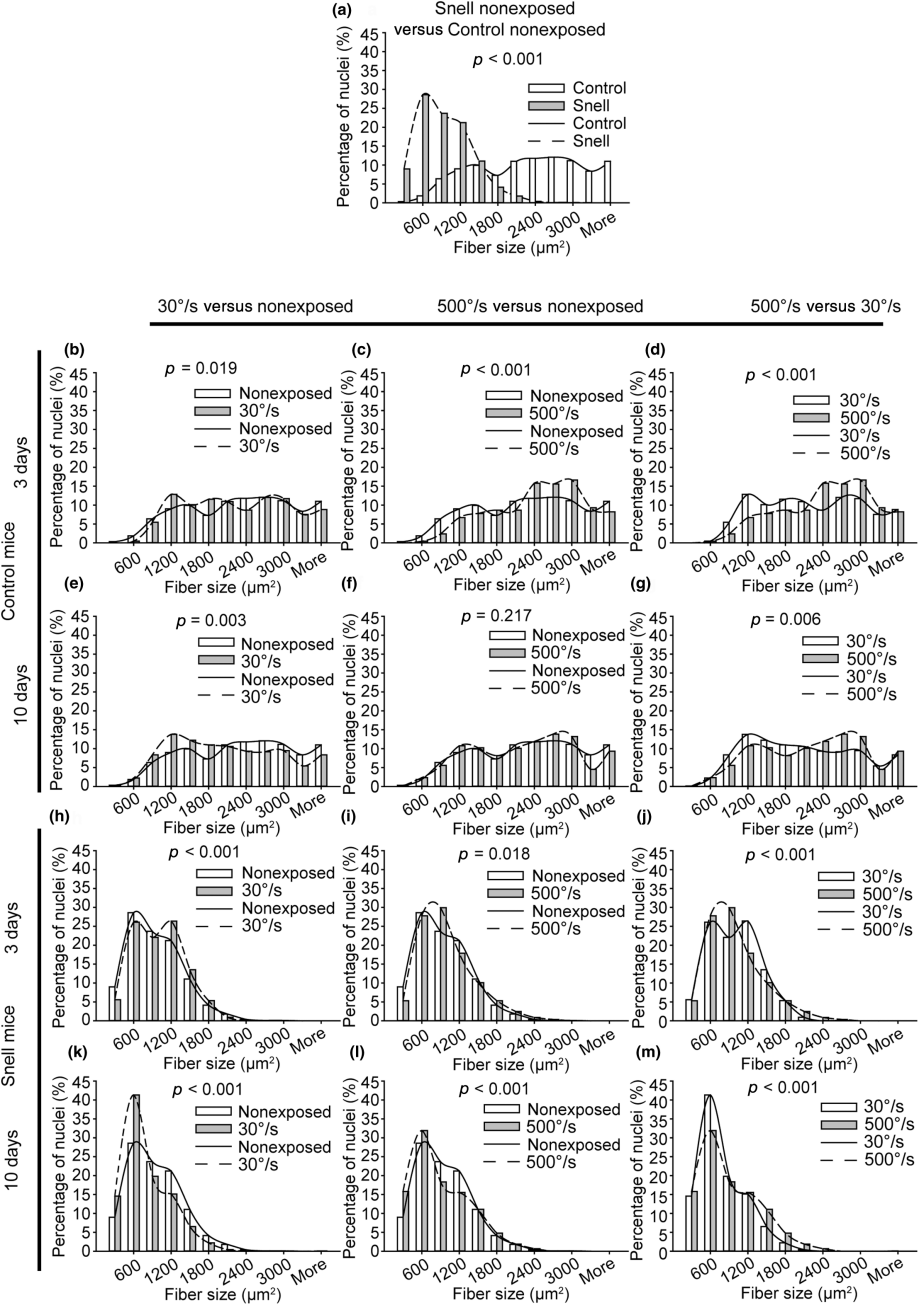
Distribution shifts to smaller muscle fibers followed the 30°/s protocol for muscles of control mice and both protocols for muscles of Snell dwarf mice—frequency distributions for nonexposed (a) and exposed (b–m) muscles (*N* = six to eight muscles evaluated per group with the exception *N* = 4 for the Snell/3 days/500°/s group). Chi‐squared analysis was performed to determine distribution alterations between groups, *p* < 0.05.

Transverse muscle sections were immunofluorescence labeled for dystrophin and nuclei to characterize muscle fiber size and myonuclear number (Figure 5a–j). The distributions for muscle fiber area were analyzed initially (Figure 6a–m). For muscle fibers of control mice, the 30°/s protocol induced a shift to smaller sizes at Days 3 and 10, *p* < 0.05 (Figure 6b,e). In contrast, the 500°/s protocol induced a shift to larger size at 3 days (*p* < 0.001) which returned to baseline by 10 days, *p* = 0.217 (Figure 6c,f). For Snell dwarf mice, muscle fibers were small at baseline compared with those of control mice, *p* < 0.001 (Figure 6a). At 3 days, distribution peaks for Snell dwarf mice shifted to larger fiber areas irrespective of protocol, *p* < 0.05 (Figure 6h,i). Then at 10 days, fiber area distribution shifted to smaller fiber sizes for both protocols for Snell dwarf mice, *p* < 0.001 (Figure 6k,l).

The protocol‐induced response in myonuclear size exhibited similarity to that observed for muscle fiber area at the 3 day time period (Figure S8a–m). For control mice, the 500°/s protocol shifted the distribution to larger sizes at 3 days, *p* = 0.006 (Figure S8c). This response was ubiquitous for muscles of Snell dwarf mice, *p* < 0.001 (Figure S8h,i). At 10 days, a distribution of larger myonuclei persisted in muscles of control mice (500°/s protocol), *p* < 0.001, and Snell dwarf mice, *p* < 0.001 (30°/s protocol) (Figure S8f,k).

To examine the effect of the protocols on nuclear shape, myonuclear circularity was evaluated (Figure S9a–m). At baseline, myonuclei were less circular for Snell dwarf mice relative to those of control mice, *p* = 0.003 (Figure S9a). A protocol‐induced distribution shift in myonuclei circularity was exclusive to the 30°/s protocol at 3 days for both genotypes, *p* < 0.05 (Figure S9b,h). This was characterized as a shift rightward to more circular myonuclei.

**FIGURE 7:**
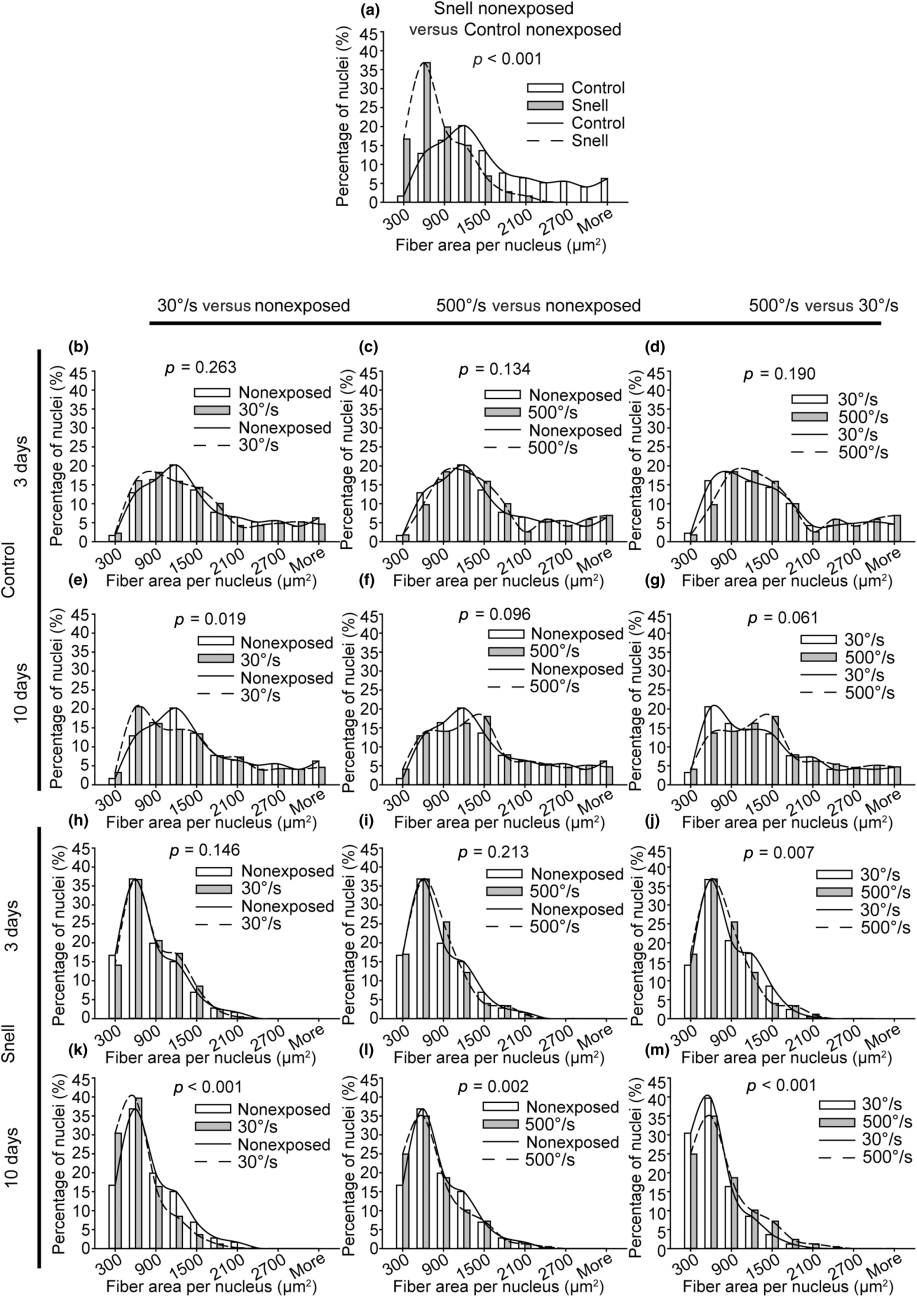
For nonexposed muscles fiber area per nucleus distribution was shifted to smaller sizes for Snell dwarf mice compared with that of control mice. Exposure to the 30°/s protocol induced a shift to smaller sizes for control mice whereas both protocols induced such a shift for Snell dwarf mice. Frequency distributions were determined for nonexposed (a) and exposed (b–m) muscles (*N* = six to eight muscles evaluated per group with the exception *N* = 4 for the Snell/3 days/500°/s group). Chi‐squared analysis was performed to determine distribution alterations between groups, *p* < 0.05.

Following the measures of muscle fiber area and myonuclear size/shape, fiber area per nucleus (i.e., an indicator of myonuclear domain) was determined (Figure 7a–m). For control mice, a shift in fiber area per nucleus was observed exclusively 10 days following the 30°/s protocol to smaller size, *p* = 0.019 (Figure 7e). Fiber area per nucleus for Snell dwarf mice was shifted to decreased size at baseline relative to that of control mice, *p* < 0.001 (Figure 7a). Shifts to even smaller size for Snell dwarf mice were observed 10 days following both protocols, *p* < 0.01 (Figure 7k,l).

## 4 DISCUSSION

The purpose of the present study was to address the research gap regarding characterization of muscles of Snell dwarf mice relative to their littermate control mice in the days following a single exposure to two distinct SSC protocols—an intermittent slow velocity (30°/s ankle rotation) contraction protocol or a continuous rapid velocity (500°/s) contraction protocol. The findings demonstrated that both protocols induced initial reductions in maximum isometric torque capacity at 5 min post‐protocol—reduced to 5% of pre‐protocol values for Snell dwarf mice and to 30% of pre‐protocol values for control mice. For control mice at 3 days, muscle mass increased by 5% and performance remained depressed at 80% of pre‐protocol. Protocol‐dependent outcomes emerged by 10 days with a muscle mass deficit following the 500°/s protocol and a return to baseline values following the 30°/s protocol. For Snell dwarf mice, already by 3 days, performance recovered from the initial deficit in maximum isometric torque and no muscle mass changes were observed. The outcomes from the 30°/s protocol for control mice and both protocols for Snell dwarf mice were accompanied by a transient increase in interstitium, increased total transcriptional output, cytokine‐mediated signaling gene upregulation, and a distribution shift to smaller muscle fibers with less fiber area per nucleus. Overall, the results supported the hypothesis that muscles of control littermate mice exhibit differential transcriptional/morphological and muscle mass/performance responses following intermittent slow velocity vs continuous rapid velocity contraction exposures while for Snell dwarf mice, such responses are more universal—a finding consistent with the remodeling observed with repeated SSC exposure in the presence of the *Pit1* mutation (Rader et al., 2018, 2022).

The muscles of Snell dwarf mice were remarkable in that performance recovered by 3 days following the initial toque deficits of the 30°/s and 500°/s protocols while muscles of control mice still exhibited depressed performance. Performance recovery by several days is not uncommon following resistance training and has been demonstrated previously (Goulart et al., 2021; Monteiro et al., 2019). However, this is striking in the present study since the performance deficits during both SSC protocols were more pronounced for Snell dwarf mice compared with those of control mice. Contraction‐induced performance deficits are typically ascribed to fatigue and/or muscle fiber ultrastructural disruption (e.g., disruption to neuromuscular junction, sarcolemma, sarcoplasmic reticulum, and/or sarcomere; Baumann et al., 2022; Chapman et al., 2006; McCully & Faulkner, 1985, 1986; Mori et al., 2014; Rader & Baker, 2017). Given the lower peak torques for the muscles Snell dwarf mice at baseline, a decreased extent of ultrastructural disruption induced by tension/strain may have occurred in the context of the *Pit1* mutation. In previous research, contraction‐induced damage has been demonstrated to be proportional to the forces and work undergone during lengthening contractions (Brooks et al., 1995). If decreased ultrastructural disruption was present for muscles of Snell dwarf mice, recovery in performance by 3 days could have resulted because of less damage to repair rather than accelerated repair. However, this possibility could not be evaluated since direct measurement of muscle fiber damage was lacking in the present study. Furthermore, performance recovery by 3 days as observed for Snell dwarf mice did not necessarily imply that the protocols induced only fatigue in the absence of muscle fiber damage. Recovery from lengthening contractions by 3 days despite evidence of ultrastructural disruption (such as sustained performance deficits 1 day post‐protocol and histological observation of muscle fiber damage) has been observed in some reports previously in mouse models (Baumann et al., 2020, 2022; Brooks, 1998). The data of the present study did provide results relevant to short‐term fatigue. While muscles of control mice incurred a degree of short‐term fatigue so that 1/3rd of the torque deficit was resolved within 6 min following the 30°/s protocol, performance deficits remained unchanged over that duration for Snell dwarf mice across protocols. This illustrated a more sustained form of fatigue and/or ultrastructural damage for Snell dwarf mice that persisted past the initial minutes following the protocols. Testing muscles at additional timepoints post‐protocol in future work would help further characterize the form of fatigue or damage present.

Torque time integral optimization (i.e., summation of mechanical stress over time) as a major factor in inducing muscle adaptation is indicated by previous data regarding wild‐type rats which demonstrate a correlation between torque time integral and anabolic signaling (Ato et al., 2016; Ochi et al., 2011). The present study also supported this notion in the context of the control mice, in particular, in that the higher torque time integral of the 30°/s protocol (relative to the 500°/s protocol) was concomitant with greater muscle adaptation in terms of performance, cytokine‐mediated signaling gene upregulation, and morphological features. However, in regards to the Snell dwarf mice, torque time integrals were especially small (15% of those for control mice) and varied between protocols for Snell dwarf mice. Therefore, torque time integral values likely did not account for the analogous transcriptional/morphological responsivity across protocols in the context of the *Pit1* mutation. Rather the Snell dwarf feature of small baseline values of muscle fiber area per myonuclei, indicative of decreased myonuclear domain, may have been a factor in the recovery/remodeling across protocols. The proposal that smaller myonuclear domains offer the capacity for responsiveness because of greater transcriptional potential has been supported in resistance training studies regarding older individuals and high responders (Dirks et al., 2017; Sieljacks et al., 2019). In these studies, small baseline myonuclear domain sizes accompanied the largest relative changes in adaptation in the form of muscle fiber size (Dirks et al., 2017; Sieljacks et al., 2019). Therefore, responsiveness to both protocols for Snell dwarf mice—despite small and varied torque time integrals—may have been possible in the presence of small myonuclear domains at baseline.

While genotype‐dependent differences were observed in how ubiquitous recovery was across both SSC protocols, several distinct morphological/transcriptional responses were in common between Snell dwarf mice and control mice when recovery did take place. Transcriptional responses included increased total RNA levels and upregulation of cytokine‐mediated signaling gene expression. Increased total RNA is indicative of ribosomal biogenesis since more than 85% of total RNA is ribosomal RNA (Figueiredo & McCarthy, 2019; Kotani et al., 2021). Ribosomal biogenesis has been observed in the days following a single bout of muscle contractions in prior research (Kotani et al., 2019, 2021; West et al., 2018). This increase in overall transcriptional output was accompanied by heightened differential gene regulation regarding cytokine‐mediated signaling. Performance and muscle mass recovery concomitant with total transcriptional levels and remodeling‐related gene expression has been observed in other contexts involving exposure to muscle overload or contractions (Baumann et al., 2023; Kirby et al., 2015; Meyer et al., 2013; Rader & Baker, 2020). Furthermore, the cytokine‐mediated signaling response, in particular, has accompanied remodeling and recovery in prior research regarding muscle contractions (Docherty et al., 2022; Ochi et al., 2011; Rader et al., 2015).

By 10 days, muscle fiber area and fiber area per nucleus shifted to smaller sizes in the instances of efficacious recovery (i.e., 30°/s protocol for control mice and both protocols for Snell dwarf mice). Since interstitial space and muscle mass were at baseline values, a shift to smaller muscle fibers indicated that transverse muscle fiber number increased. Such a finding was observed in previous research in our laboratory regarding 1 month of volitional weight‐lifting in rats (Rader et al., 2014). Increased fiber number has been observed by others (as well as in synergist ablation overload models) and the mechanisms of fiber splitting and *denovo* fiber formation have been proposed (Ho et al., 1980; Murach et al., 2019; Soffe et al., 2016; Tamaki et al., 1992). An advantage of smaller muscle fibers include greater oxygen diffusion efficiency and, therefore, energetics during contractions (Murach et al., 2019; Rader et al., 2014).

In addition to analyzing muscle fiber morphology, myonuclear size, shape, and density were evaluated. Despite the potential for nuclear shape and size to reflect extent of chromatin condensation and, possibly, transcriptional potential, SSC‐induced alterations in these features did not consistently accompany transcriptional/recovery responses across genotypes in the present study (Mascetti et al., 2001; Rader & Baker, 2022; Versaevel et al., 2012). Rather evidence for myonuclear addition following SSC exposure was observed—given the increase in myonuclear density as indicated by changes in muscle fiber area per nucleus—and reliably concomitant with recovery. Prior research regarding training/detraining also demonstrated evidence for myonuclear addition (Bruusgaard et al., 2010; Rader & Baker, 2022). In other studies, myonuclei accretion was observed following weighted wheel running in mice (Dungan et al., 2019; Murach et al., 2022). Such an adaptation is proposed to support remodeling due to greater transcriptional capacity and support eventual hypertrophy with repeated exposure to muscle contractions. Overall, the present research supports the notion that increasing cross‐sectional fiber number with decreased fiber area per nucleus are sensitive adaptive responses following muscular activity.

The rapid recovery following a single exposure of SSCs for Snell dwarf mice was consistent with the responsivity observed with repeated intermittent slow velocity SSC exposures reported previously (Rader et al., 2018, 2022). The present findings extend this earlier research by demonstrating effective recovery also following continuous rapid velocity SSCs in addition to intermittent slow velocity SSC exposures for Snell dwarf mice. Such SSCs are relevant since activities such as plyometrics have been promoted as a viable alternative to slower SSCs (such as in traditional resistance training) for inducing muscle adaptation (Grgic et al., 2021; MacDonald et al., 2012; Morris et al., 2022). Our results indicate that a spectrum of physical activities have the potential to offset the weakness exhibited with this model of hypopituitarism. While the results of the present study are significant, the findings should not be directly applied to humans without additional consideration. For instance, fiber type differences exist between gastrocnemius muscles of mice and humans—those of mice predominantly consisting of type II muscle fibers whereas muscles of humans are a mix of type I and II fibers (Burkholder et al., 1994; Edgerton et al., 1975; Rader et al., 2022; Schoenfeld et al., 2020). An active area of research has been investigating the extent to which responses to stress/stimuli such as susceptibility to contraction‐induced damage and adaptation are fiber type specific (Fry, 2004; Macaluso et al., 2012; Macpherson et al., 1996; Schoenfeld et al., 2020). Therefore, the present research should be regarded as supportive (rather than definitive) for the proposal that a spectrum of physical activities have the potential to offset the weakness exhibited by this preclinical model of hypopituitarism. Further investigation in the human population is warranted to achieve more conclusive and translational inferences.

## AUTHOR CONTRIBUTIONS

E.P.R. and B.A.B. designed the study and interpreted the data; E.P.R., K.A.M. and B.A.B. performed the experiments; E.P.R. wrote the original draft; K.A.M. and B.A.B. reviewed and edited the manuscript. All authors approved the final version of the manuscript.

## CONFLICT OF INTEREST STATEMENT

The authors declare no conflicts of interest.

## PUBLICATION DISCLAIMERS STATEMENT

The findings and conclusions in this report are those of the author(s) and do not necessarily represent the official position of the National Institute for Occupational Safety and Health, Centers for Disease Control and Prevention.

## DATA AVAILABILTY STATEMENT

The data that support the findings of this study are available from the corresponding author upon reasonable request.

## ETHICS STATEMENT

All animal experimental procedures performed in this study were approved by the Animal Care and Use Committee at the National Institute for Occupational Safety and Health in Morgantown, WV.

## Supporting information


Figure S1.

Figure S2.

Figure S3.

Figure S4.

Figure S5.

Figure S6.

Figure S7.

Figure S8.

Figure S9.

Table S1.

Table S2.

Table S3.

Table S4.

Table S5.

Table S6.

Table S7.

Table S8.

Table S9.

Table S10.

Table S11.

Table S12.

Table S13.

Table S14.

Table S15.
