# Supplementary figures and images for: Transcriptional and morphological responses following distinct muscle contraction protocols for Snell dwarf (Pit1dw/dw ) mice
Source: Physiol Rep. 2024 Sep 3;12(17):e70027. doi: 10.14814/phy2.70027 (PMC11371489; doi:10.14814/phy2.70027)

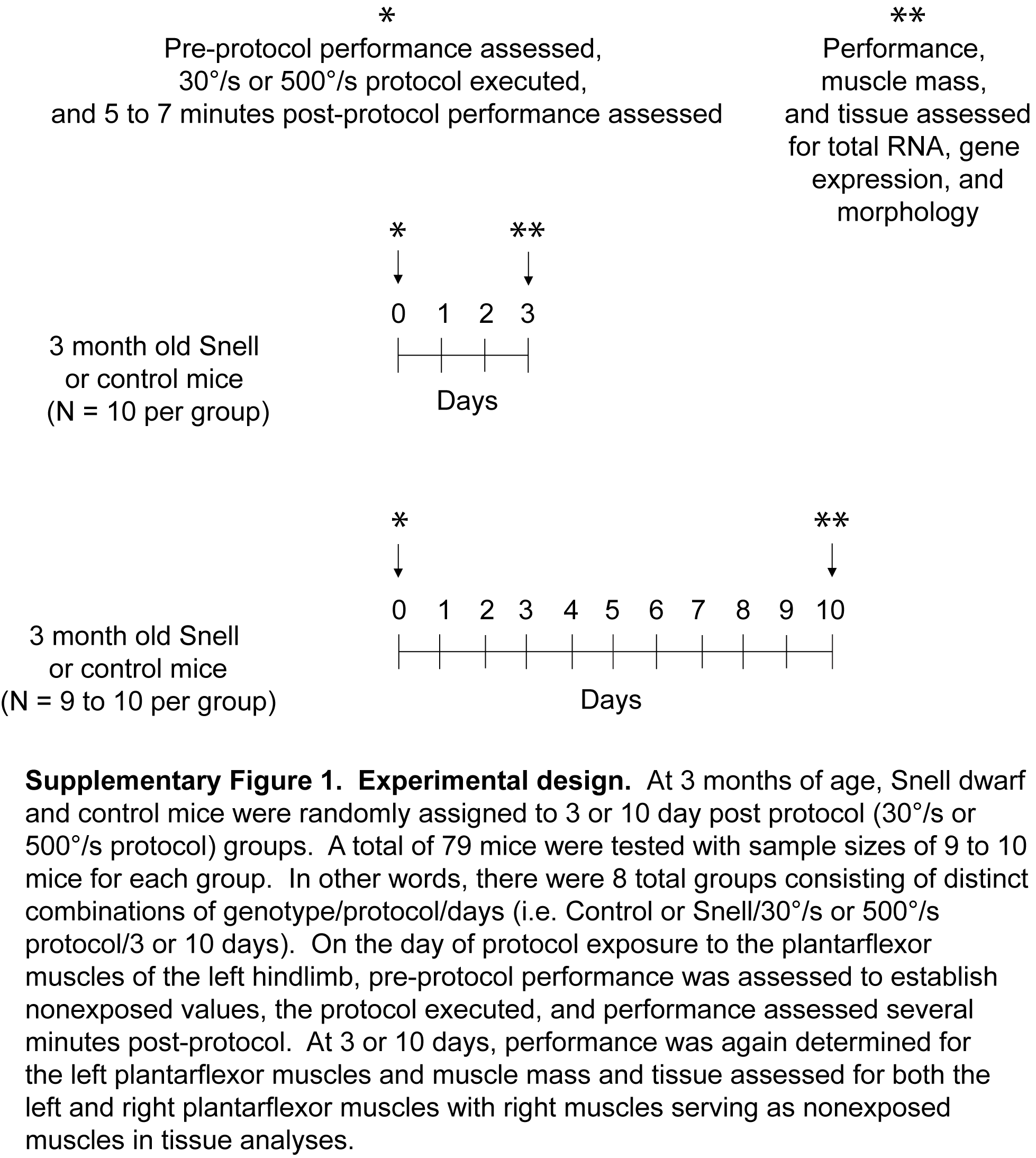

Supplement: Supplementary file 1 — Figure S1. [file PHY2-12-e70027-s003.tif]

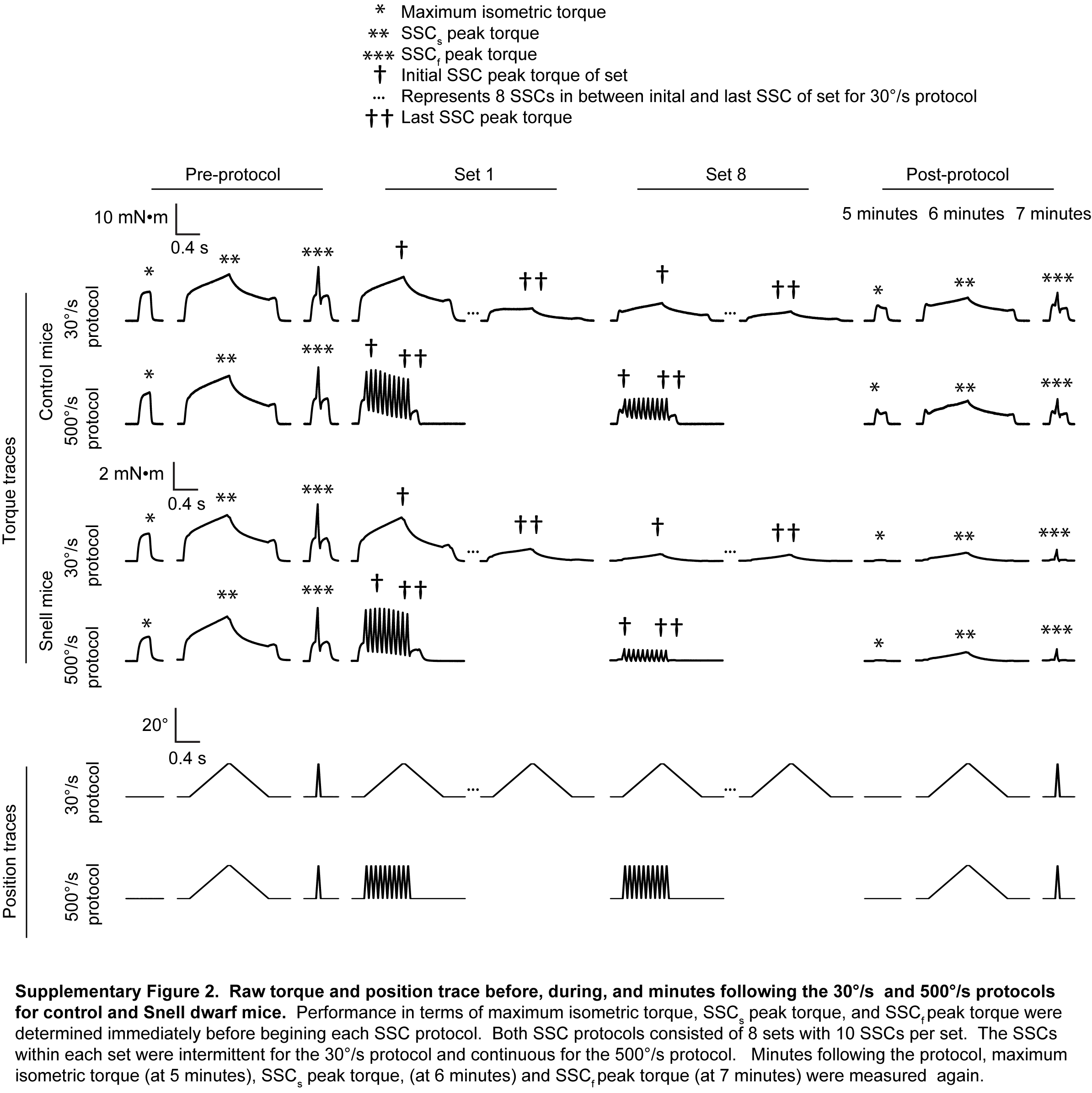

Supplement: Supplementary file 2 — Figure S2. [file PHY2-12-e70027-s023.tif]

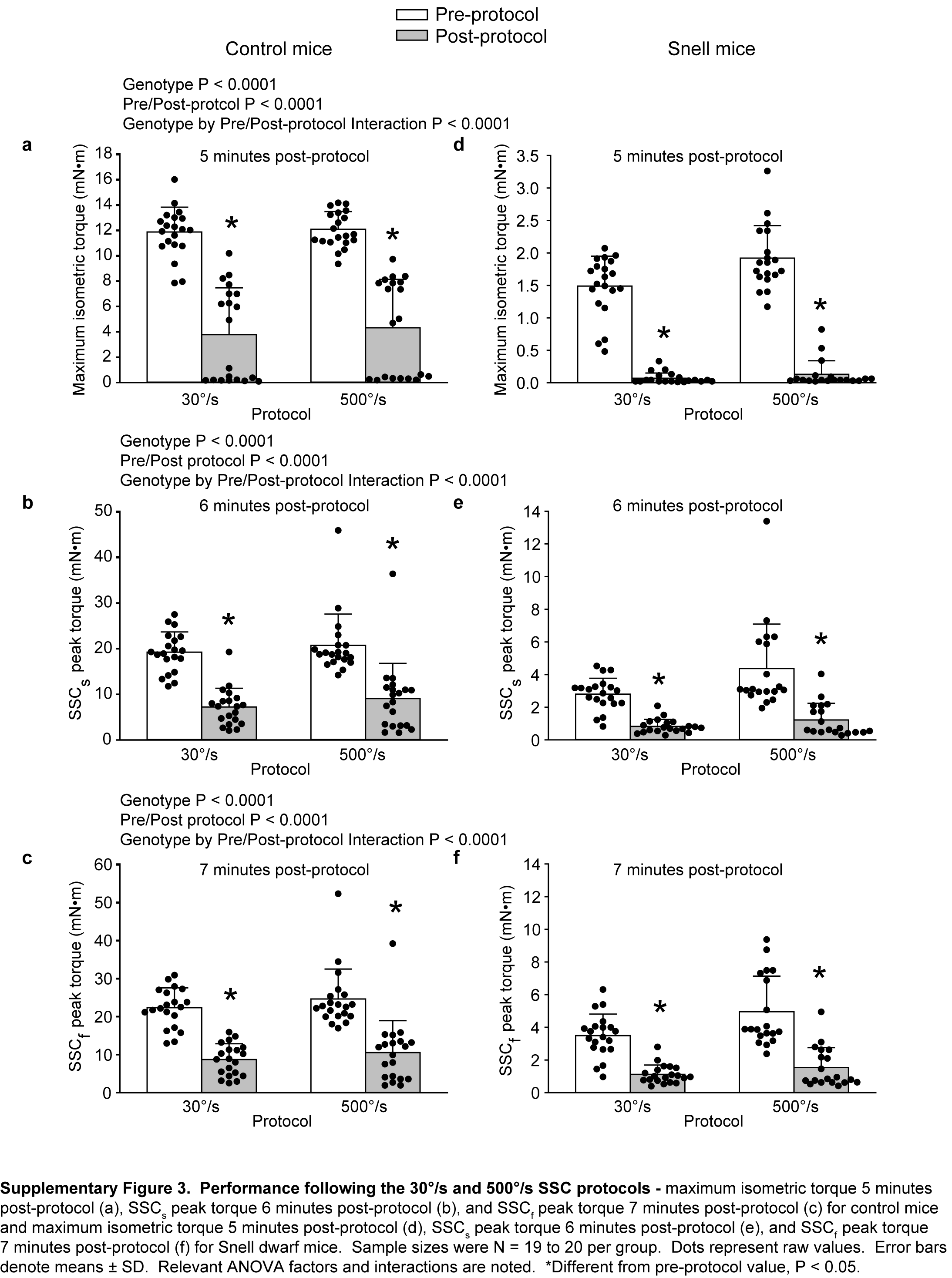

Supplement: Supplementary file 3 — Figure S3. [file PHY2-12-e70027-s016.tif]

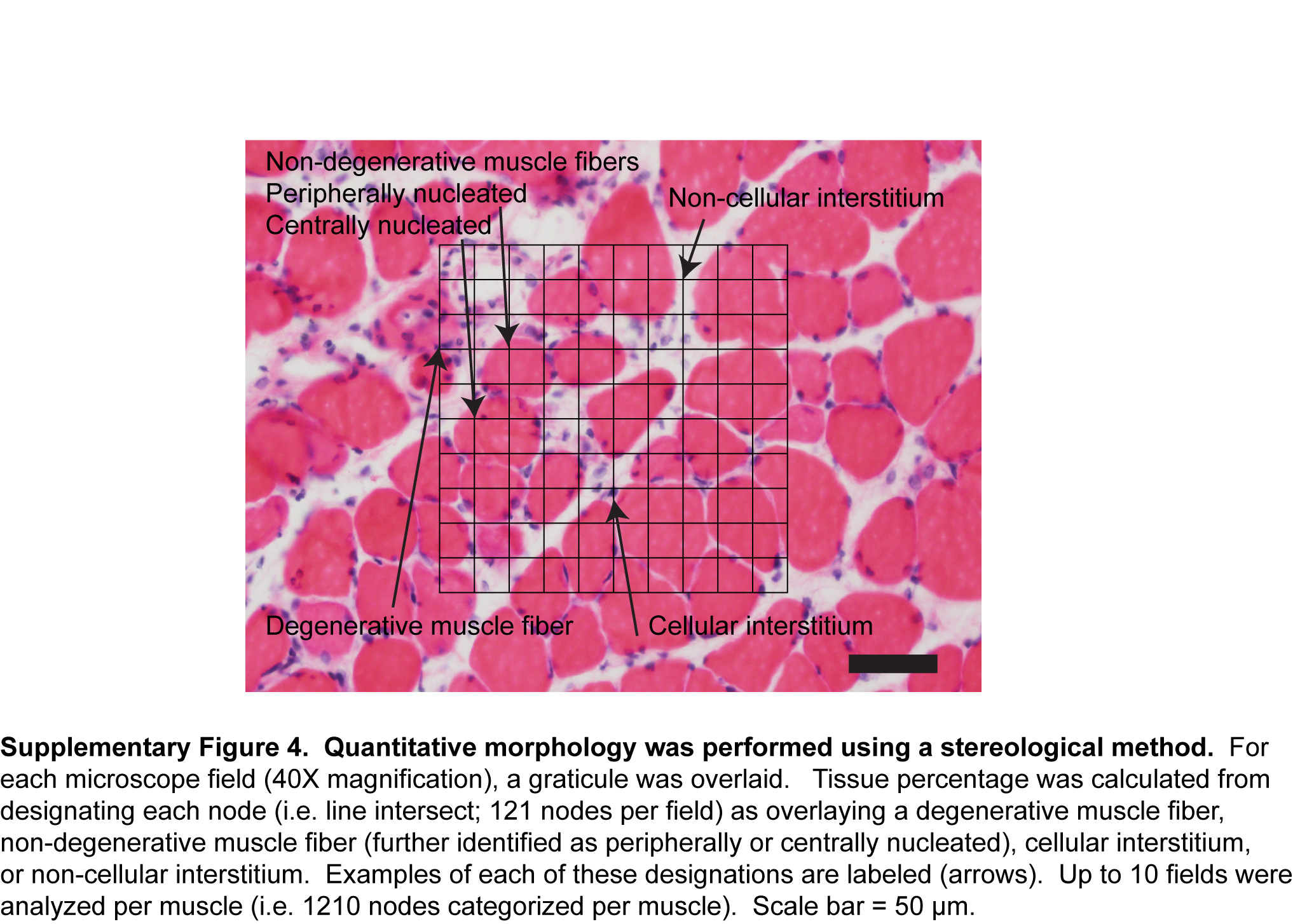

Supplement: Supplementary file 4 — Figure S4. [file PHY2-12-e70027-s009.tif]

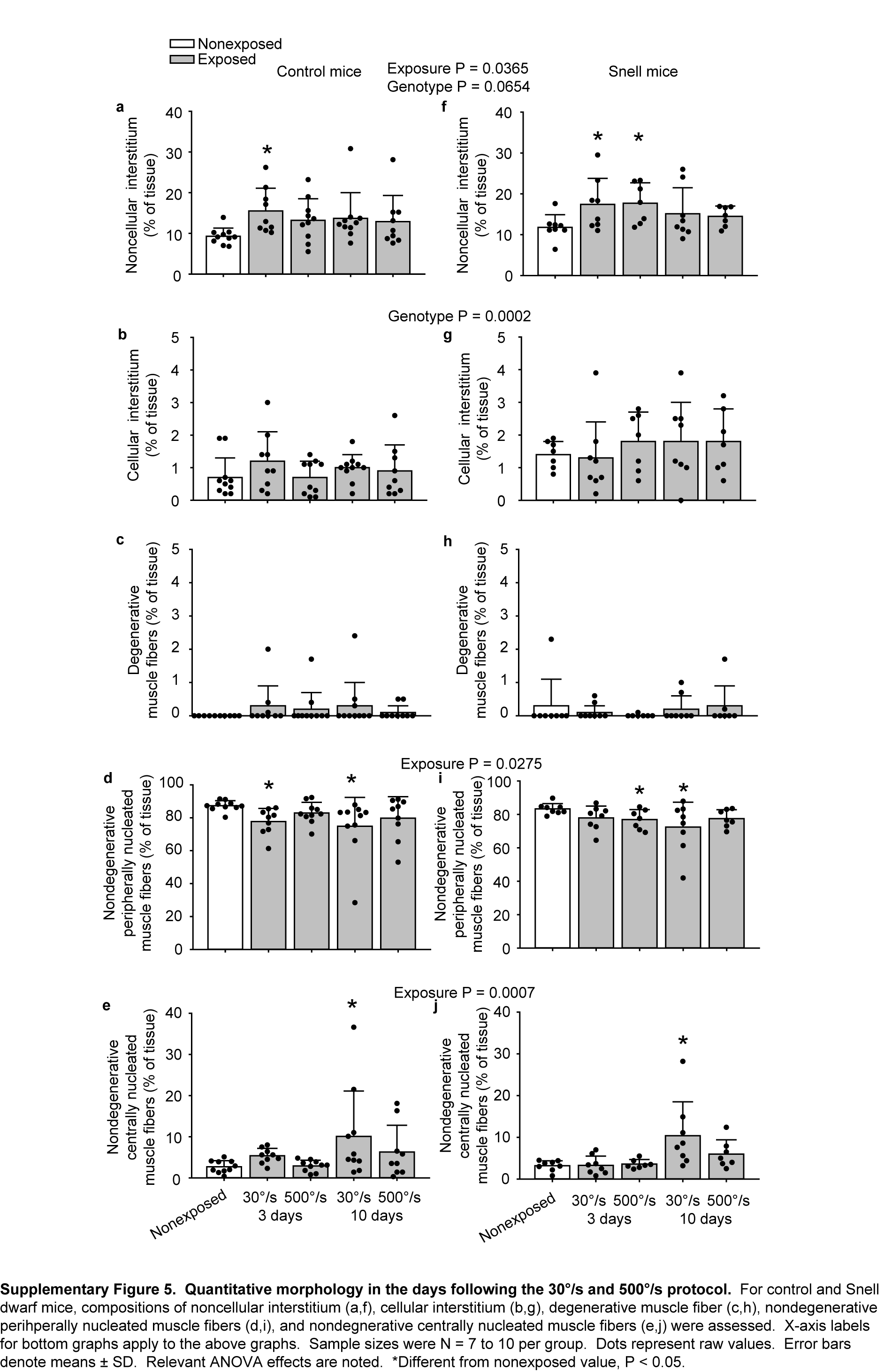

Supplement: Supplementary file 5 — Figure S5. [file PHY2-12-e70027-s018.tif]

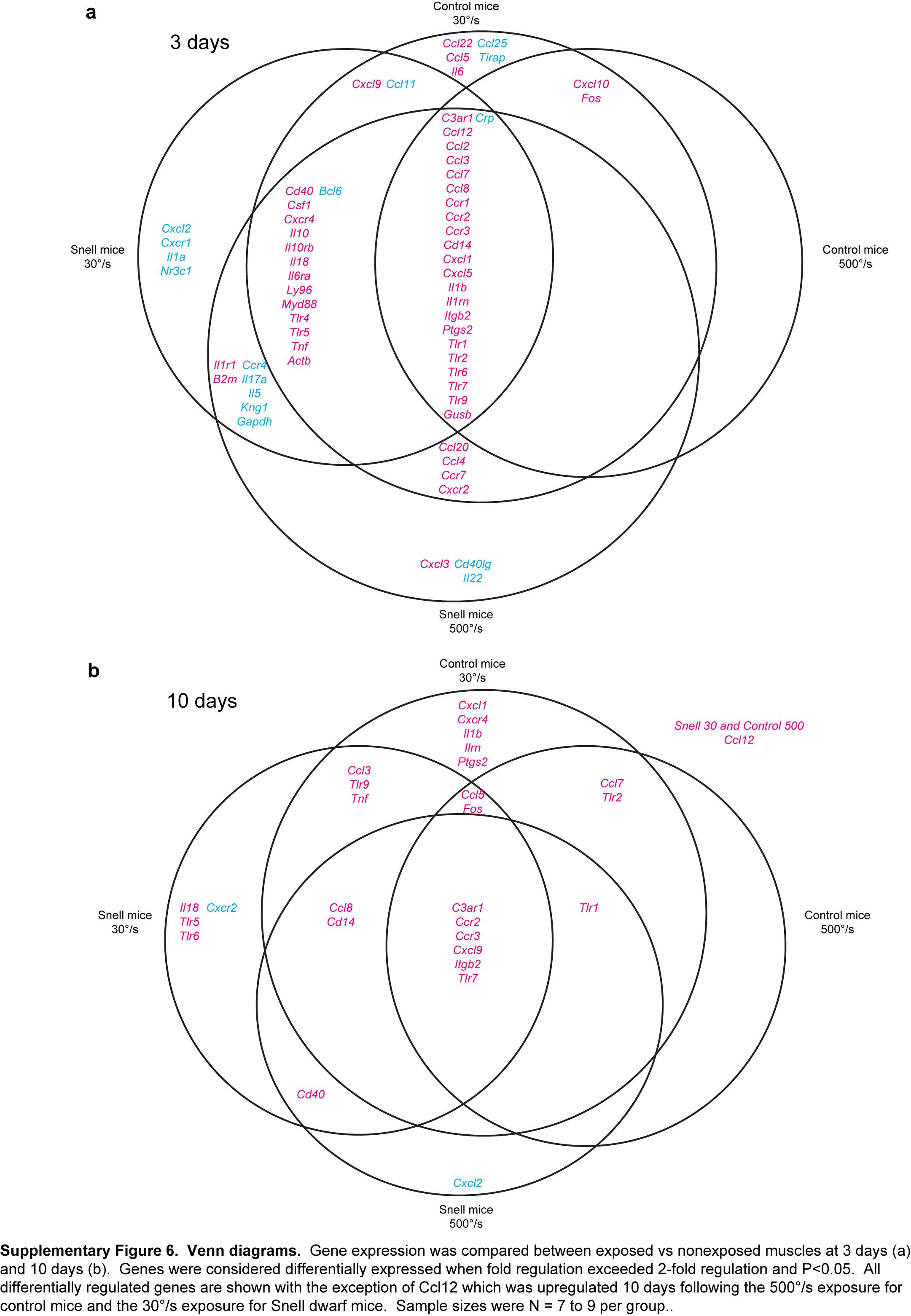

Supplement: Supplementary file 6 — Figure S6. [file PHY2-12-e70027-s021.tif]

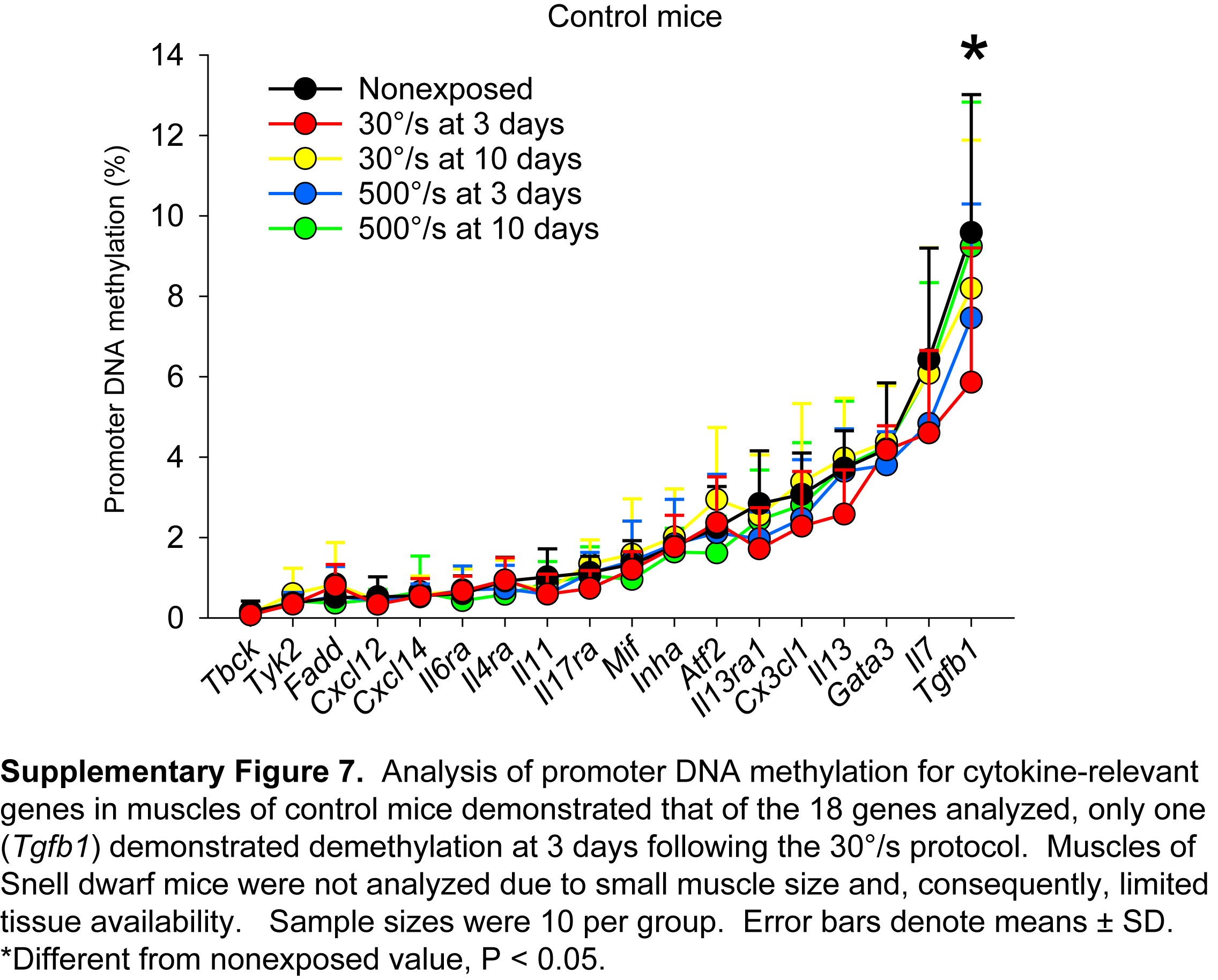

Supplement: Supplementary file 7 — Figure S7. [file PHY2-12-e70027-s005.tif]

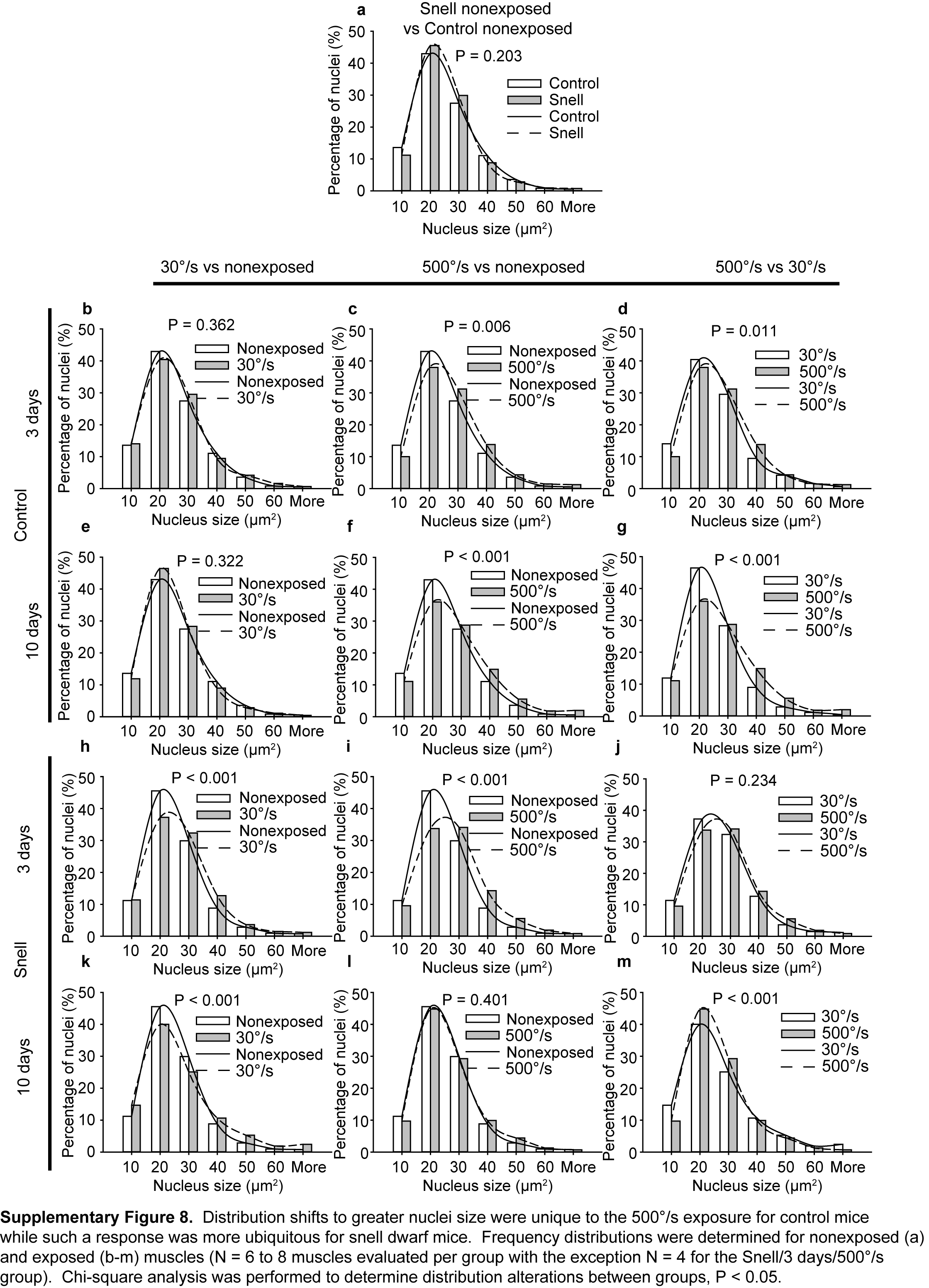

Supplement: Supplementary file 8 — Figure S8. [file PHY2-12-e70027-s013.tif]

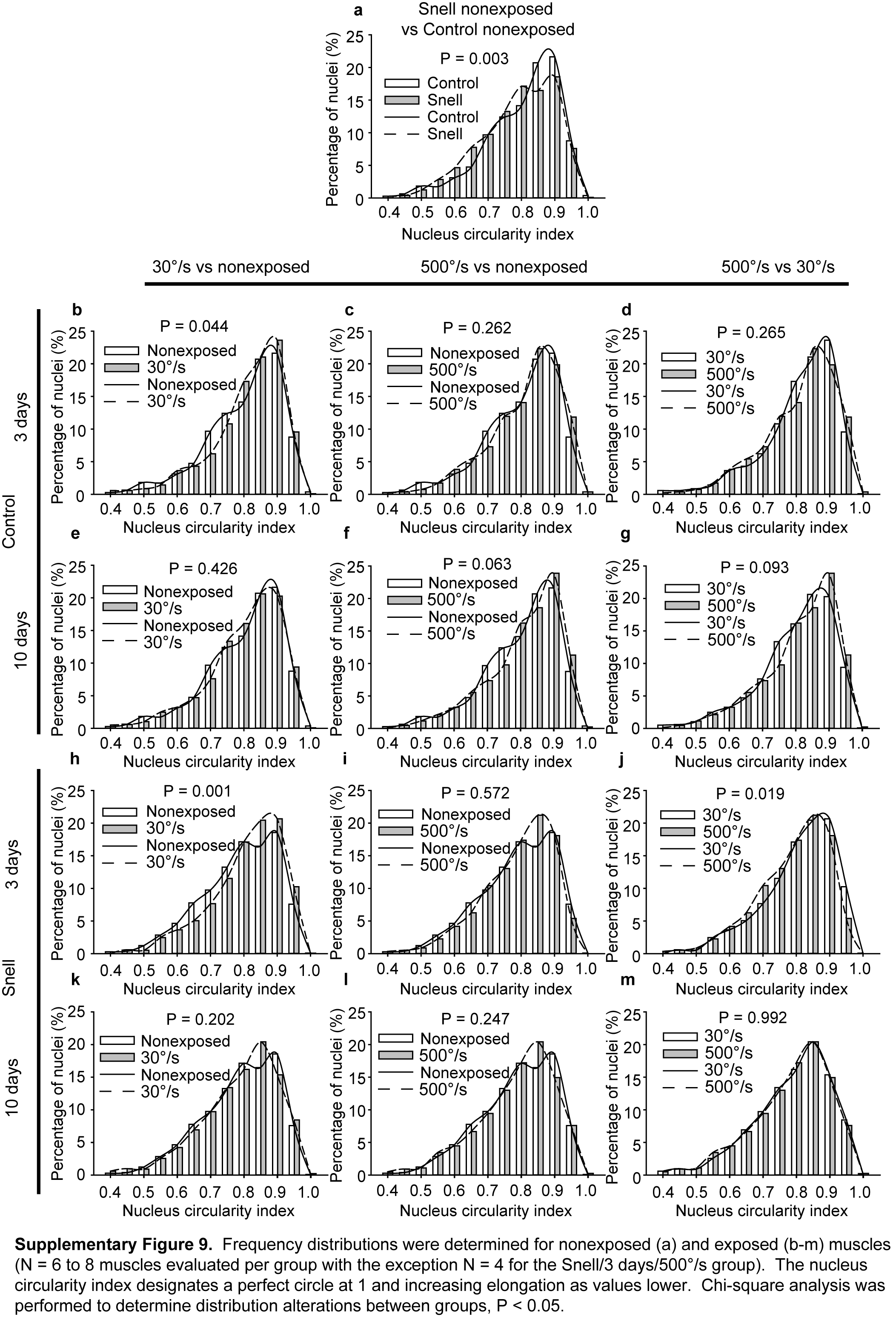

Supplement: Supplementary file 9 — Figure S9. [file PHY2-12-e70027-s002.tif]
