# Supplementary material for: Transcriptional and morphological responses following distinct muscle contraction protocols for Snell dwarf (Pit1dw/dw ) mice
Source: Physiol Rep. 2024 Sep 3;12(17):e70027. doi: 10.14814/phy2.70027 (PMC11371489; doi:10.14814/phy2.70027)
Supplement: Supplementary file 10 — Table S1. [file PHY2-12-e70027-s004.docx]

**Supplementary Table 1. Muscle mass data for exposed and nonexposed plantarflexor muscles of Snell dwarf and control mice.**

| Control |  |  |  | Muscle mass (mg) | | | Normalized muscle mass (mg/mm) | | |
| --- | --- | --- | --- | --- | --- | --- | --- | --- | --- |
|  | 3 days |  |  |  |  |  |  |  |  |
|  |  | 30°/s |  | Gastrocnemius | Plantaris | Soleus | Gastrocnemius | Plantaris | Soleus |
|  |  |  | Right | 129.0 ± 9.2 | 16.1 ± 1.8 | 8.0 ± 0.8 | 7.04 ± 0.41 | 0.88± 0.08 | 0.44 ± 0.04 |
|  |  |  | Left | 138.3 ± 9.3* | 18.1 ± 1.5* | 8.5 ± 0.7 | 7.56 ± 0.39* | 0.99 ± 0.07* | 0.46 ± 0.03* |
|  |  | 500°/s |  |  |  |  |  |  |  |
|  |  |  | Right | 125.6 ± 7.3 | 14.8 ± 0.9 | 7.2 ± 1.2 | 6.91 ± 0.37 | 0.82 ± 0.05 | 0.39 ± 0.07 |
|  |  |  | Left | 130.0 ± 6.5* | 17.5 ± 1.6* | 7.7 ± 1.0* | 7.12 ± 0.33* | 0.96 ± 0.09* | 0.42 ± 0.05* |
|  | 10 days |  |  |  |  |  |  |  |  |
|  |  | 30°/s |  |  |  |  |  |  |  |
|  |  |  | Right | 128.4 ± 12.3 | 15.5 ± 1.5 | 7.8 ± 1.0 | 7.06 ± 0.55 | 0.85 ± 0.07 | 0.43 ± 0.05 |
|  |  |  | Left | 128.1 ± 12.4 | 17.3 ± 2.1* | 8.1 ± 1.3 | 7.04 ± 0.59 | 0.95 ± 0.10* | 0.44 ± 0.07 |
|  |  | 500°/s |  |  |  |  |  |  |  |
|  |  |  | Right | 127.2 ± 9.5 | 16.4 ± 2.0 | 8.4 ± 1.0 | 6.96 ± 0.46 | 0.90 ± 0.10 | 0.46 ± 0.05 |
|  |  |  | Left | 122.1 ± 9.2* | 16.8 ± 1.9 | 7.7 ± 1.2* | 6.69 ± 0.42* | 0.92 ± 0.10 | 0.42 ± 0.06* |
|  | | | | | | | | | |
| Snell |  |  |  |  |  |  |  |  |  |
|  | 3 days |  |  |  |  |  |  |  |  |
|  |  | 30°/s |  |  |  |  |  |  |  |
|  |  |  | Right | 29.6 ± 2.5 | 3.6 ± 0.4 | 1.6 ± 0.2 | 2.46 ± 0.18 | 0.30 ± 0.03 | 0.13 ± 0.02 |
|  |  |  | Left | 30.1 ± 5.0 | 3.9 ± 0.7 | 1.8 ± 0.2 | 2.51 ± 0.38 | 0.33 ± 0.05 | 0.15 ± 0.02 |
|  |  | 500°/s |  |  |  |  |  |  |  |
|  |  |  | Right | 27.8 ± 3.5 | 3.6 ± 0.5 | 1.8 ± 0.3 | 2.36 ± 0.21 | 0.31 ± 0.03 | 0.15 ± 0.02 |
|  |  |  | Left | 28.6 ± 3.6 | 3.9 ± 0.7 | 1.8 ± 0.4 | 2.41 ± 0.23 | 0.33 ± 0.05 | 0.15 ± 0.03 |
|  | 10 days |  |  |  |  |  |  |  |  |
|  |  | 30°/s |  |  |  |  |  |  |  |
|  |  |  | Right | 27.3 ± 4.2 | 3.8 ± 0.8 | 1.7 ± 0.4 | 2.42 ± 0.22 | 0.32 ± 0.06 | 0.14 ± 0.03 |
|  |  |  | Left | 28.5 ± 3.3 | 3.6 ± 0.7 | 1.7 ± 0.3 | 2.34 ± 0.31 | 0.31 ± 0.06 | 0.14 ± 0.03 |
|  |  | 500°/s |  |  |  |  |  |  |  |
|  |  |  | Right | 30.2 ± 4.5 | 4.0 ± 0.5 | 1.8 ± 0.3 | 2.52 ± 0.28 | 0.33 ± 0.03 | 0.15 ± 0.03 |
|  |  |  | Left | 29.0 ± 3.8 | 3.9 ± 0.4 | 1.8 ± 0.2 | 2.43 ± 0.25 | 0.33 ± 0.03 | 0.15 ± 0.02 |

Values are expressed as means ± SD. Sample sizes were N = 9 to 10 per group. All Snell dwarf values were different from comparable control values, P < 0.05. *Different from comparable right value; P < 0.05.
