# Supplementary material for: Transcriptional and morphological responses following distinct muscle contraction protocols for Snell dwarf (Pit1dw/dw ) mice
Source: Physiol Rep. 2024 Sep 3;12(17):e70027. doi: 10.14814/phy2.70027 (PMC11371489; doi:10.14814/phy2.70027)
Supplement: Supplementary file 11 — Table S2. [file PHY2-12-e70027-s006.docx]

**Supplementary Table 2. Performance data during distinct SSC protocols for plantarflexor muscles of Snell dwarf and control mice.**

|  |  | 30°/s | 500°/s |
| --- | --- | --- | --- |
| Control |  |  |  |
| Set 1 | Initial SSC peak torque (mN·m) | 15.62 ± 4.25 | 23.90 ± 8.03* |
|  | Last SSC peak torque (mN·m) | 5.20 ± 2.11 | 19.93 ± 7.01* |
|  | Total work (mJ) | 6.23 ± 2.32 | 13.81 ± 2.98* |
|  | Total power (mW) | 0.47 ± 0.17 | 17.70 ± 3.82* |
|  | Total torque time integral (mN·m·s) | 74.59 ± 27.78 | 9.69 ± 2.09* |
| Set 8 | Initial SSC peak torque (mN·m) | 5.57 ± 2.50 | 11.19 ± 9.15* |
|  | Last SSC peak torque (mN·m) | 3.98 ± 1.63 | 10.17 ± 8.21* |
|  | Total work (mJ) | 2.53 ± 1.23 | 6.30 ± 4.51* |
|  | Total power (mW) | 0.19 ± 0.09 | 8.07 ± 5.78* |
|  | Total torque time integral (mN·m·s) | 30.26 ± 14.68 | 4.42 ± 3.16* |
| Snell |  |  |  |
| Set 1 | Initial SSC peak torque (mN·m) | 2.60 ± 0.97 | 4.63 ± 1.96 |
|  | Last SSC peak torque (mN·m) | 0.94 ± 0.42 | 4.22 ± 1.70* |
|  | Total work (mJ) | 0.99 ± 0.36 | 2.37 ± 0.73 |
|  | Total power (mW) | 0.07 ± 0.03 | 3.04 ± 0.94* |
|  | Total torque time integral (mN·m·s) | 11.91 ± 4.34 | 1.66 ± 0.51* |
| Set 8 | Initial SSC peak torque (mN·m) | 0.74 ± 0.41 | 1.56 ± 1.24 |
|  | Last SSC peak torque (mN·m) | 0.68 ± 0.37 | 1.36 ± 1.07 |
|  | Total work (mJ) | 0.29 ± 0.15 | 0.58 ± 0.51 |
|  | Total power (mW) | 0.02 ± 0.01 | 0.74 ± 0.65 |
|  | Total torque time integral (mN·m·s) | 3.52 ± 1.85 | 0.41 ± 0.36 |

Values are expressed as means ± SD. Sample sizes were N = 19 to 20 per group. In general, greater values of torque, work, and power were reached during the 500°/s protocol while greater torque time integral resulted during the 30°/s protocol. All Snell dwarf values were different from comparable control values with exceptions of 30°/s total power values and 500°/s set 8 total impulse values, P < 0.05. *Different from comparable 30°/s value; P < 0.05.
