# Supplementary material for: Transcriptional and morphological responses following distinct muscle contraction protocols for Snell dwarf (Pit1dw/dw ) mice
Source: Physiol Rep. 2024 Sep 3;12(17):e70027. doi: 10.14814/phy2.70027 (PMC11371489; doi:10.14814/phy2.70027)
Supplement: Supplementary file 12 — Table S3. [file PHY2-12-e70027-s020.docx]

|  | RefSeq | Snell dwarf nonexposed vs control nonexposed | |  |  | RefSeq | Snell dwarf nonexposed vs control nonexposed | |
| --- | --- | --- | --- | --- | --- | --- | --- | --- |
|  |  |  |  |  |  |  |  |  |
|  |  | Fold change | P value |  |  |  | Fold change | P value |
| *Bcl6* | NM_009744 | 1.04 | 0.808444 |  | *Il17a* | NM_010552 | 1.45 | 0.056583 |
| *C3* | NM_009778 | 0.35 | 0.048268 |  | *Il18* | NM_008360 | 0.35 | 0.000000 |
| *C3ar1* | NM_009779 | 0.99 | 0.902420 |  | *Il1a* | NM_010554 | 1.35 | 0.049869 |
| *C4b* | NM_009780 | 0.70 | 0.161295 |  | *Il1b* | NM_008361 | 2.14 | 0.016357 |
| *Ccl1* | NM_011329 | 1.39 | 0.193577 |  | *Il1r1* | NM_008362 | 0.96 | 0.798369 |
| *Ccl11* | NM_011330 | 0.31 | 0.000001 |  | *Il1rap* | NM_008364 | 0.93 | 0.578735 |
| *Ccl12* | NM_011331 | 1.48 | 0.281457 |  | *Il1rn* | NM_031167 | 2.20 | 0.041086 |
| *Ccl17* | NM_011332 | 1.40 | 0.184984 |  | *Il22* | NM_016971 | 1.53 | 0.200108 |
| *Ccl19* | NM_011888 | 0.57 | 0.000671 |  | *Il23a* | NM_031252 | 1.72 | 0.014431 |
| *Ccl2* | NM_011333 | 2.20 | 0.005663 |  | *Il23r* | NM_144548 | 1.19 | 0.489572 |
| *Ccl20* | NM_016960 | 1.42 | 0.404910 |  | *Il5* | NM_010558 | 1.54 | 0.015032 |
| *Ccl22* | NM_009137 | 2.19 | 0.007140 |  | *Il6* | NM_001314054 | 0.94 | 0.771942 |
| *Ccl24* | NM_019577 | 0.97 | 0.951692 |  | *Il6ra* | NM_010559 | 1.00 | 0.813212 |
| *Ccl25* | NM_009138 | 0.51 | 0.003533 |  | *Il7* | NM_008371 | 1.23 | 0.263055 |
| *Ccl3* | NM_011337 | 1.64 | 0.019190 |  | *Il9* | NM_008373 | 1.32 | 0.239836 |
| *Ccl4* | NM_013652 | 1.58 | 0.009426 |  | *Itgb2* | NM_008404 | 1.06 | 0.608470 |
| *Ccl5* | NM_013653 | 1.10 | 0.444994 |  | *Kng1* | NM_023125 | 1.38 | 0.306698 |
| *Ccl7* | NM_013654 | 1.77 | 0.080167 |  | *Lta* | NM_010735 | ND | ND |
| *Ccl8* | NM_021443 | 0.20 | 0.002736 |  | *Ltb* | NM_008518 | 1.26 | 0.292648 |
| *Ccr1* | NM_009912 | 1.96 | 0.000443 |  | *Ly96* | NM_016923 | 1.09 | 0.528771 |
| *Ccr2* | NM_009915 | 1.02 | 0.783430 |  | *Myd88* | NM_010851 | 0.88 | 0.307699 |
| *Ccr3* | NM_009914 | 0.59 | 0.610676 |  | *Nfkb1* | NM_008689 | 1.18 | 0.007104 |
| *Ccr4* | NM_009916 | 1.69 | 0.029309 |  | *Nos2* | NM_001313921 | 1.10 | 0.406671 |
| *Ccr7* | NM_007719 | 0.85 | 0.710060 |  | *Nr3c1* | NM_008173 | 1.03 | 0.737201 |
| *Cd14* | NM_009841 | 0.68 | 0.005439 |  | *Ptgs2* | NM_011198 | 0.88 | 0.499317 |
| *Cd40* | NM_011611 | 0.58 | 0.121579 |  | *Ripk2* | NM_138952 | 1.22 | 0.090294 |
| *Cd40lg* | NM_011616 | 1.55 | 0.017242 |  | *Sele* | NM_011345 | 1.34 | 0.731576 |
| *Cebpb* | NM_009883 | 0.76 | 0.005809 |  | *Tirap* | NM_054096 | 0.94 | 0.665099 |
| *Crp* | NM_007768 | 1.69 | 0.050323 |  | *Tlr1* | NM_030682 | 2.01 | 0.068493 |
| *Csf1* | NM_007778 | 0.81 | 0.107112 |  | *Tlr2* | NM_011905 | 1.09 | 0.432074 |
| *Cxcl1* | NM_008176 | 1.06 | 0.935726 |  | *Tlr3* | NM_126166 | 0.85 | 0.187014 |
| *Cxcl10* | NM_021274 | 2.08 | 0.009330 |  | *Tlr4* | NM_021297 | 1.57 | 0.010141 |
| *Cxcl11* | NM_019494 | 2.23 | 0.029080 |  | *Tlr5* | NM_016928 | 0.79 | 0.137492 |
| *Cxcl2* | NM_009140 | 1.50 | 0.045961 |  | *Tlr6* | NM_011604 | 0.72 | 0.044612 |
| *Cxcl3* | NM_203320 | 1.61 | 0.022658 |  | *Tlr7* | NM_133211 | 0.72 | 0.247546 |
| *Cxcl5* | NM_009141 | 1.72 | 0.170026 |  | *Tlr9* | NM_031178 | 1.34 | 0.015313 |
| *Cxcl9* | NM_008599 | 0.56 | 0.079789 |  | *Tnf* | NM_013693 | 0.78 | 0.250315 |
| *Cxcr1* | NM_178241 | 1.16 | 0.933094 |  | *Tnfsf14* | NM_019418 | 1.44 | 0.036203 |
| *Cxcr2* | NM_009909 | 4.34 | 0.007914 |  | *Tollip* | NM_023764 | 1.19 | 0.017069 |
| *Cxcr4* | NM_009911 | 2.53 | 0.000013 |  | *Actb* | NM_007393 | 1.06 | 0.389759 |
| *Fasl* | NM_010177 | 1.30 | 0.078231 |  | *B2m* | NM_009735 | 0.53 | 0.000409 |
| *Fos* | NM_010234 | 1.43 | 0.084880 |  | *Gapdh* | NM_008084 | 1.40 | 0.015409 |
| *Ifng* | NM_008337 | 1.77 | 0.091094 |  | *Gusb* | NM_010368 | 1.12 | 0.189867 |
| *Il10* | NM_010548 | 1.53 | 0.246030 |  |  |  |  |  |
| *Il10rb* | NM_008349 | 1.09 | 0.467113 |  |  |  |  |  |

**­Supplementary Table 3. Differential mRNA levels of nonexposed muscles of Snell dwarf mice vs those of control mice.**

Expression which surpassed 2-fold regulation (below 0.5 fold change or above 2 fold change) with a P value < 0.05 was considered differentially expressed. ND, Not detected. Not highlighted – unchanged, Orange – upregulated, Blue - downregulated. Sample sizes were N = 8 per group.
