# Supplementary material for: Transcriptional and morphological responses following distinct muscle contraction protocols for Snell dwarf (Pit1dw/dw ) mice
Source: Physiol Rep. 2024 Sep 3;12(17):e70027. doi: 10.14814/phy2.70027 (PMC11371489; doi:10.14814/phy2.70027)
Supplement: Supplementary file 13 — Table S4. [file PHY2-12-e70027-s010.docx]

|  | RefSeq | 30°/s protocol vs nonexposed | |  |  | RefSeq | 30°/s protocol vs nonexposed | |
| --- | --- | --- | --- | --- | --- | --- | --- | --- |
|  |  |  |  |  |  |  |  |  |
|  |  | Fold change | P value |  |  |  | Fold change | P value |
| *Bcl6* | NM_009744 | 0.50 | 0.000040 |  | *Il17a* | NM_010552 | 0.57 | 0.005869 |
| *C3* | NM_009778 | 0.61 | 0.202976 |  | *Il18* | NM_008360 | 2.53 | 0.000846 |
| *C3ar1* | NM_009779 | 31.80 | 0.000087 |  | *Il1a* | NM_010554 | 0.68 | 0.034768 |
| *C4b* | NM_009780 | 1.19 | 0.743118 |  | *Il1b* | NM_008361 | 21.30 | 0.000257 |
| *Ccl1* | NM_011329 | 1.27 | 0.377084 |  | *Il1r1* | NM_008362 | 1.83 | 0.000385 |
| *Ccl11* | NM_011330 | 0.36 | 0.000013 |  | *Il1rap* | NM_008364 | 1.00 | 0.847103 |
| *Ccl12* | NM_011331 | 15.79 | 0.000098 |  | *Il1rn* | NM_031167 | 13.98 | 0.000426 |
| *Ccl17* | NM_011332 | 1.49 | 0.170572 |  | *Il22* | NM_016971 | 0.57 | 0.148528 |
| *Ccl19* | NM_011888 | 0.50 | 0.000599 |  | *Il23a* | NM_031252 | 1.48 | 0.111961 |
| *Ccl2* | NM_011333 | 28.56 | 0.000096 |  | *Il23r* | NM_144548 | 0.58 | 0.108085 |
| *Ccl20* | NM_016960 | 3.31 | 0.010521 |  | *Il5* | NM_010558 | 0.72 | 0.031134 |
| *Ccl22* | NM_009137 | 2.62 | 0.000463 |  | *Il6* | NM_001314054 | 4.16 | 0.002690 |
| *Ccl24* | NM_019577 | 1.44 | 0.122530 |  | *Il6ra* | NM_010559 | 3.13 | 0.000014 |
| *Ccl25* | NM_009138 | 0.47 | 0.003466 |  | *Il7* | NM_008371 | 0.61 | 0.008857 |
| *Ccl3* | NM_011337 | 9.14 | 0.002317 |  | *Il9* | NM_008373 | 0.64 | 0.135225 |
| *Ccl4* | NM_013652 | 2.38 | 0.008174 |  | *Itgb2* | NM_008404 | 20.50 | 0.000020 |
| *Ccl5* | NM_013653 | 3.75 | 0.001560 |  | *Kng1* | NM_023125 | 0.53 | 0.090804 |
| *Ccl7* | NM_013654 | 42.21 | 0.000148 |  | *Lta* | NM_010735 | ND | ND |
| *Ccl8* | NM_021443 | 25.65 | 0.000002 |  | *Ltb* | NM_008518 | 1.03 | 0.961633 |
| *Ccr1* | NM_009912 | 16.48 | 0.000001 |  | *Ly96* | NM_016923 | 3.04 | 0.000018 |
| *Ccr2* | NM_009915 | 17.65 | 0.000000 |  | *Myd88* | NM_010851 | 3.52 | 0.000011 |
| *Ccr3* | NM_009914 | 30.56 | 0.000001 |  | *Nfkb1* | NM_008689 | 1.25 | 0.005838 |
| *Ccr4* | NM_009916 | 0.75 | 0.205198 |  | *Nos2* | NM_001313921 | 0.87 | 0.242129 |
| *Ccr7* | NM_007719 | 2.86 | 0.001629 |  | *Nr3c1* | NM_008173 | 0.48 | 0.000029 |
| *Cd14* | NM_009841 | 9.55 | 0.000053 |  | *Ptgs2* | NM_011198 | 7.37 | 0.000112 |
| *Cd40* | NM_011611 | 3.63 | 0.000014 |  | *Ripk2* | NM_138952 | 1.21 | 0.039160 |
| *Cd40lg* | NM_011616 | 0.85 | 0.824608 |  | *Sele* | NM_011345 | 0.86 | 0.382284 |
| *Cebpb* | NM_009883 | 0.63 | 0.003012 |  | *Tirap* | NM_054096 | 0.49 | 0.003159 |
| *Crp* | NM_007768 | 0.42 | 0.008896 |  | *Tlr1* | NM_030682 | 40.36 | 0.001105 |
| *Csf1* | NM_007778 | 2.11 | 0.000012 |  | *Tlr2* | NM_011905 | 8.28 | 0.000003 |
| *Cxcl1* | NM_008176 | 12.11 | 0.000221 |  | *Tlr3* | NM_126166 | 1.68 | 0.004478 |
| *Cxcl10* | NM_021274 | 5.74 | 0.000140 |  | *Tlr4* | NM_021297 | 2.95 | 0.000031 |
| *Cxcl11* | NM_019494 | 0.65 | 0.118837 |  | *Tlr5* | NM_016928 | 2.16 | 0.001088 |
| *Cxcl2* | NM_009140 | 0.99 | 0.849618 |  | *Tlr6* | NM_011604 | 3.27 | 0.000033 |
| *Cxcl3* | NM_203320 | 1.59 | 0.040495 |  | *Tlr7* | NM_133211 | 11.88 | 0.000357 |
| *Cxcl5* | NM_009141 | 54.65 | 0.001973 |  | *Tlr9* | NM_031178 | 8.48 | 0.000056 |
| *Cxcl9* | NM_008599 | 2.88 | 0.021945 |  | *Tnf* | NM_013693 | 3.45 | 0.000904 |
| *Cxcr1* | NM_178241 | 0.58 | 0.271250 |  | *Tnfsf14* | NM_019418 | 1.54 | 0.010192 |
| *Cxcr2* | NM_009909 | 3.25 | 0.035404 |  | *Tollip* | NM_023764 | 0.72 | 0.000173 |
| *Cxcr4* | NM_009911 | 7.39 | 0.000180 |  | *Actb* | NM_007393 | 2.20 | 0.000191 |
| *Fasl* | NM_010177 | 2.11 | 0.070209 |  | *B2m* | NM_009735 | 1.57 | 0.035382 |
| *Fos* | NM_010234 | 7.65 | 0.000763 |  | *Gapdh* | NM_008084 | 0.33 | 0.000058 |
| *Ifng* | NM_008337 | 1.64 | 0.065923 |  | *Gusb* | NM_010368 | 4.25 | 0.000033 |
| *Il10* | NM_010548 | 4.00 | 0.003376 |  |  |  |  |  |
| *Il10rb* | NM_008349 | 2.42 | 0.000392 |  |  |  |  |  |

**Supplementary Table 4. Differential mRNA levels of muscles of control mice 3 days post 30°/s protocol vs nonexposed muscles.**

Expression which surpassed 2-fold regulation (below 0.5 fold change or above 2 fold change) with a P value < 0.05 was considered differentially expressed. ND, Not detected. Not highlighted – unchanged, Orange – upregulated, Blue - downregulated. Sample sizes were N = 7-8 per group.
