# Supplementary material for: Transcriptional and morphological responses following distinct muscle contraction protocols for Snell dwarf (Pit1dw/dw ) mice
Source: Physiol Rep. 2024 Sep 3;12(17):e70027. doi: 10.14814/phy2.70027 (PMC11371489; doi:10.14814/phy2.70027)
Supplement: Supplementary file 14 — Table S5. [file PHY2-12-e70027-s017.docx]

|  | RefSeq | 500°/s protocol vs nonexposed | |  |  | RefSeq | 500°/s protocol vs nonexposed | |
| --- | --- | --- | --- | --- | --- | --- | --- | --- |
|  |  |  |  |  |  |  |  |  |
|  |  | Fold change | P value |  |  |  | Fold change | P value |
| *Bcl6* | NM_009744 | 0.56 | 0.000852 |  | *Il17a* | NM_010552 | 0.59 | 0.052272 |
| *C3* | NM_009778 | 0.97 | 0.826321 |  | *Il18* | NM_008360 | 1.78 | 0.020353 |
| *C3ar1* | NM_009779 | 12.34 | 0.033149 |  | *Il1a* | NM_010554 | 0.74 | 0.024459 |
| *C4b* | NM_009780 | 1.69 | 0.181643 |  | *Il1b* | NM_008361 | 3.98 | 0.031368 |
| *Ccl1* | NM_011329 | 0.93 | 0.960122 |  | *Il1r1* | NM_008362 | 1.33 | 0.108314 |
| *Ccl11* | NM_011330 | 0.56 | 0.000881 |  | *Il1rap* | NM_008364 | 0.85 | 0.400907 |
| *Ccl12* | NM_011331 | 9.97 | 0.000592 |  | *Il1rn* | NM_031167 | 6.14 | 0.004405 |
| *Ccl17* | NM_011332 | 1.44 | 0.145608 |  | *Il22* | NM_016971 | 0.53 | 0.049762 |
| *Ccl19* | NM_011888 | 0.71 | 0.073637 |  | *Il23a* | NM_031252 | 1.37 | 0.156284 |
| *Ccl2* | NM_011333 | 12.02 | 0.001878 |  | *Il23r* | NM_144548 | 0.62 | 0.116176 |
| *Ccl20* | NM_016960 | 1.37 | 0.385402 |  | *Il5* | NM_010558 | 0.85 | 0.282356 |
| *Ccl22* | NM_009137 | 1.88 | 0.046071 |  | *Il6* | NM_001314054 | 1.61 | 0.055158 |
| *Ccl24* | NM_019577 | 1.53 | 0.072151 |  | *Il6ra* | NM_010559 | 1.75 | 0.040201 |
| *Ccl25* | NM_009138 | 0.77 | 0.305006 |  | *Il7* | NM_008371 | 0.57 | 0.019823 |
| *Ccl3* | NM_011337 | 4.94 | 0.006671 |  | *Il9* | NM_008373 | 0.62 | 0.107634 |
| *Ccl4* | NM_013652 | 1.37 | 0.136485 |  | *Itgb2* | NM_008404 | 9.51 | 0.015445 |
| *Ccl5* | NM_013653 | 2.11 | 0.140633 |  | *Kng1* | NM_023125 | 0.68 | 0.234949 |
| *Ccl7* | NM_013654 | 16.41 | 0.002015 |  | *Lta* | NM_010735 | ND | ND |
| *Ccl8* | NM_021443 | 15.67 | 0.000967 |  | *Ltb* | NM_008518 | 1.03 | 0.852758 |
| *Ccr1* | NM_009912 | 7.48 | 0.003411 |  | *Ly96* | NM_016923 | 1.89 | 0.024642 |
| *Ccr2* | NM_009915 | 8.36 | 0.002210 |  | *Myd88* | NM_010851 | 1.89 | 0.034464 |
| *Ccr3* | NM_009914 | 13.01 | 0.006203 |  | *Nfkb1* | NM_008689 | 1.03 | 0.563589 |
| *Ccr4* | NM_009916 | 0.84 | 0.290802 |  | *Nos2* | NM_001313921 | 0.74 | 0.263678 |
| *Ccr7* | NM_007719 | 1.33 | 0.210749 |  | *Nr3c1* | NM_008173 | 0.63 | 0.009345 |
| *Cd14* | NM_009841 | 4.37 | 0.000171 |  | *Ptgs2* | NM_011198 | 2.47 | 0.003163 |
| *Cd40* | NM_011611 | 1.77 | 0.044371 |  | *Ripk2* | NM_138952 | 0.98 | 0.919004 |
| *Cd40lg* | NM_011616 | 0.83 | 0.331180 |  | *Sele* | NM_011345 | 0.75 | 0.311228 |
| *Cebpb* | NM_009883 | 0.67 | 0.000914 |  | *Tirap* | NM_054096 | 0.66 | 0.117079 |
| *Crp* | NM_007768 | 0.50 | 0.041785 |  | *Tlr1* | NM_030682 | 16.48 | 0.006975 |
| *Csf1* | NM_007778 | 1.46 | 0.037649 |  | *Tlr2* | NM_011905 | 3.63 | 0.006910 |
| *Cxcl1* | NM_008176 | 3.74 | 0.022612 |  | *Tlr3* | NM_126166 | 1.14 | 0.354432 |
| *Cxcl10* | NM_021274 | 3.28 | 0.033830 |  | *Tlr4* | NM_021297 | 1.94 | 0.016815 |
| *Cxcl11* | NM_019494 | 0.64 | 0.103881 |  | *Tlr5* | NM_016928 | 1.62 | 0.050983 |
| *Cxcl2* | NM_009140 | 0.73 | 0.210762 |  | *Tlr6* | NM_011604 | 2.21 | 0.010036 |
| *Cxcl3* | NM_203320 | 1.50 | 0.129958 |  | *Tlr7* | NM_133211 | 6.29 | 0.007459 |
| *Cxcl5* | NM_009141 | 13.32 | 0.021370 |  | *Tlr9* | NM_031178 | 3.83 | 0.018948 |
| *Cxcl9* | NM_008599 | 2.03 | 0.243352 |  | *Tnf* | NM_013693 | 1.35 | 0.344362 |
| *Cxcr1* | NM_178241 | 0.59 | 0.117241 |  | *Tnfsf14* | NM_019418 | 1.02 | 0.721687 |
| *Cxcr2* | NM_009909 | 1.49 | 0.198524 |  | *Tollip* | NM_023764 | 0.76 | 0.003455 |
| *Cxcr4* | NM_009911 | 2.70 | 0.077361 |  | *Actb* | NM_007393 | 1.31 | 0.083724 |
| *Fasl* | NM_010177 | 1.31 | 0.299801 |  | *B2m* | NM_009735 | 1.16 | 0.366951 |
| *Fos* | NM_010234 | 4.70 | 0.002600 |  | *Gapdh* | NM_008084 | 0.55 | 0.008634 |
| *Ifng* | NM_008337 | 1.05 | 0.447371 |  | *Gusb* | NM_010368 | 2.34 | 0.019272 |
| *Il10* | NM_010548 | 4.79 | 0.164141 |  |  |  |  |  |
| *Il10rb* | NM_008349 | 1.54 | 0.095026 |  |  |  |  |  |

**Supplementary Table 5. Differential mRNA levels of muscles of control mice 3 days post 500°/s protocol vs nonexposed muscles**.

Expression which surpassed 2-fold regulation (below 0.5 fold change or above 2 fold change) with a P value < 0.05 was considered differentially expressed. ND, Not detected. Not highlighted – unchanged, Orange – upregulated, Blue - downregulated. Sample sizes were N = 8 per group.
