# Supplementary material for: Transcriptional and morphological responses following distinct muscle contraction protocols for Snell dwarf (Pit1dw/dw ) mice
Source: Physiol Rep. 2024 Sep 3;12(17):e70027. doi: 10.14814/phy2.70027 (PMC11371489; doi:10.14814/phy2.70027)
Supplement: Supplementary file 15 — Table S6. [file PHY2-12-e70027-s019.docx]

|  | RefSeq | 500°/s protocol vs  30°/s protocol | |  |  | RefSeq | 500°/s protocol vs  30°/s protocol | |
| --- | --- | --- | --- | --- | --- | --- | --- | --- |
|  |  |  |  |  |  |  |  |  |
|  |  | Fold change | P value |  |  |  | Fold change | P value |
| *Bcl6* | NM_009744 | 1.12 | 0.347020 |  | *Il17a* | NM_010552 | 1.04 | 0.394070 |
| *C3* | NM_009778 | 1.59 | 0.182619 |  | *Il18* | NM_008360 | 0.70 | 0.223885 |
| *C3ar1* | NM_009779 | 0.39 | 0.130416 |  | *Il1a* | NM_010554 | 1.10 | 0.658843 |
| *C4b* | NM_009780 | 1.42 | 0.255994 |  | *Il1b* | NM_008361 | 0.19 | 0.005336 |
| *Ccl1* | NM_011329 | 0.73 | 0.386375 |  | *Il1r1* | NM_008362 | 0.73 | 0.164482 |
| *Ccl11* | NM_011330 | 1.53 | 0.022224 |  | *Il1rap* | NM_008364 | 0.85 | 0.458920 |
| *Ccl12* | NM_011331 | 0.63 | 0.212226 |  | *Il1rn* | NM_031167 | 0.44 | 0.039035 |
| *Ccl17* | NM_011332 | 0.97 | 0.973955 |  | *Il22* | NM_016971 | 0.93 | 0.603298 |
| *Ccl19* | NM_011888 | 1.42 | 0.106964 |  | *Il23a* | NM_031252 | 0.93 | 0.835910 |
| *Ccl2* | NM_011333 | 0.42 | 0.047423 |  | *Il23r* | NM_144548 | 1.07 | 0.883474 |
| *Ccl20* | NM_016960 | 0.41 | 0.035161 |  | *Il5* | NM_010558 | 1.17 | 0.211167 |
| *Ccl22* | NM_009137 | 0.72 | 0.229001 |  | *Il6* | NM_001314054 | 0.39 | 0.014720 |
| *Ccl24* | NM_019577 | 1.06 | 0.732847 |  | *Il6ra* | NM_010559 | 0.56 | 0.034338 |
| *Ccl25* | NM_009138 | 1.65 | 0.029093 |  | *Il7* | NM_008371 | 0.94 | 0.939822 |
| *Ccl3* | NM_011337 | 0.54 | 0.143453 |  | *Il9* | NM_008373 | 0.97 | 0.991835 |
| *Ccl4* | NM_013652 | 0.58 | 0.109180 |  | *Itgb2* | NM_008404 | 0.46 | 0.126284 |
| *Ccl5* | NM_013653 | 0.56 | 0.572593 |  | *Kng1* | NM_023125 | 1.29 | 0.351887 |
| *Ccl7* | NM_013654 | 0.39 | 0.040520 |  | *Lta* | NM_010735 | ND | ND |
| *Ccl8* | NM_021443 | 0.61 | 0.189075 |  | *Ltb* | NM_008518 | 1.00 | 0.886604 |
| *Ccr1* | NM_009912 | 0.45 | 0.056020 |  | *Ly96* | NM_016923 | 0.62 | 0.104799 |
| *Ccr2* | NM_009915 | 0.47 | 0.029528 |  | *Myd88* | NM_010851 | 0.54 | 0.046832 |
| *Ccr3* | NM_009914 | 0.43 | 0.063461 |  | *Nfkb1* | NM_008689 | 0.83 | 0.138976 |
| *Ccr4* | NM_009916 | 1.12 | 0.580277 |  | *Nos2* | NM_001313921 | 0.84 | 0.849055 |
| *Ccr7* | NM_007719 | 0.46 | 0.027889 |  | *Nr3c1* | NM_008173 | 1.31 | 0.124405 |
| *Cd14* | NM_009841 | 0.46 | 0.010384 |  | *Ptgs2* | NM_011198 | 0.33 | 0.001666 |
| *Cd40* | NM_011611 | 0.49 | 0.017893 |  | *Ripk2* | NM_138952 | 0.81 | 0.068397 |
| *Cd40lg* | NM_011616 | 0.97 | 0.715034 |  | *Sele* | NM_011345 | 0.87 | 0.733547 |
| *Cebpb* | NM_009883 | 1.06 | 0.756594 |  | *Tirap* | NM_054096 | 1.36 | 0.144062 |
| *Crp* | NM_007768 | 1.19 | 0.424957 |  | *Tlr1* | NM_030682 | 0.41 | 0.070768 |
| *Csf1* | NM_007778 | 0.69 | 0.025036 |  | *Tlr2* | NM_011905 | 0.44 | 0.009586 |
| *Cxcl1* | NM_008176 | 0.31 | 0.020127 |  | *Tlr3* | NM_126166 | 0.68 | 0.048115 |
| *Cxcl10* | NM_021274 | 0.57 | 0.247775 |  | *Tlr4* | NM_021297 | 0.66 | 0.090911 |
| *Cxcl11* | NM_019494 | 0.98 | 0.992496 |  | *Tlr5* | NM_016928 | 0.75 | 0.235486 |
| *Cxcl2* | NM_009140 | 0.74 | 0.244776 |  | *Tlr6* | NM_011604 | 0.68 | 0.135099 |
| *Cxcl3* | NM_203320 | 0.94 | 0.742195 |  | *Tlr7* | NM_133211 | 0.53 | 0.157599 |
| *Cxcl5* | NM_009141 | 0.24 | 0.041476 |  | *Tlr9* | NM_031178 | 0.45 | 0.058727 |
| *Cxcl9* | NM_008599 | 0.71 | 0.828650 |  | *Tnf* | NM_013693 | 0.39 | 0.014485 |
| *Cxcr1* | NM_178241 | 1.02 | 0.663872 |  | *Tnfsf14* | NM_019418 | 0.66 | 0.087842 |
| *Cxcr2* | NM_009909 | 0.46 | 0.086570 |  | *Tollip* | NM_023764 | 1.04 | 0.568904 |
| *Cxcr4* | NM_009911 | 0.36 | 0.057526 |  | *Actb* | NM_007393 | 0.60 | 0.019033 |
| *Fasl* | NM_010177 | 0.62 | 0.434124 |  | *B2m* | NM_009735 | 0.74 | 0.372791 |
| *Fos* | NM_010234 | 0.61 | 0.184016 |  | *Gapdh* | NM_008084 | 1.66 | 0.050681 |
| *Ifng* | NM_008337 | 0.64 | 0.559096 |  | *Gusb* | NM_010368 | 0.55 | 0.052323 |
| *Il10* | NM_010548 | 1.20 | 0.425864 |  |  |  |  |  |
| *Il10rb* | NM_008349 | 0.64 | 0.171563 |  |  |  |  |  |

**­Supplementary Table 6. Differential mRNA levels of muscles of control mice 3 days post 500°/s protocol vs 30°/s protocol.**

Expression which surpassed 2-fold regulation (below 0.5 fold change or above 2 fold change) with a P value < 0.05 was considered differentially expressed. ND, Not detected. Not highlighted – unchanged, Orange – upregulated, Blue - downregulated. Sample sizes were N = 7-8 per group.
