# Supplementary material for: Transcriptional and morphological responses following distinct muscle contraction protocols for Snell dwarf (Pit1dw/dw ) mice
Source: Physiol Rep. 2024 Sep 3;12(17):e70027. doi: 10.14814/phy2.70027 (PMC11371489; doi:10.14814/phy2.70027)
Supplement: Supplementary file 16 — Table S7. [file PHY2-12-e70027-s011.docx]

|  | RefSeq | 30°/s protocol vs nonexposed | |  |  | RefSeq | 30°/s protocol vs nonexposed | |
| --- | --- | --- | --- | --- | --- | --- | --- | --- |
|  |  |  |  |  |  |  |  |  |
|  |  | Fold change | P value |  |  |  | Fold change | P value |
| *Bcl6* | NM_009744 | 0.79 | 0.281308 |  | *Il17a* | NM_010552 | 1.08 | 0.846826 |
| *C3* | NM_009778 | 1.07 | 0.840249 |  | *Il18* | NM_008360 | 1.71 | 0.003645 |
| *C3ar1* | NM_009779 | 3.44 | 0.001025 |  | *Il1a* | NM_010554 | 0.97 | 0.982903 |
| *C4b* | NM_009780 | 1.62 | 0.132854 |  | *Il1b* | NM_008361 | 3.10 | 0.003004 |
| *Ccl1* | NM_011329 | 1.55 | 0.101805 |  | *Il1r1* | NM_008362 | 1.24 | 0.162515 |
| *Ccl11* | NM_011330 | 0.72 | 0.079410 |  | *Il1rap* | NM_008364 | 1.15 | 0.177479 |
| *Ccl12* | NM_011331 | 1.48 | 0.214780 |  | *Il1rn* | NM_031167 | 2.80 | 0.001441 |
| *Ccl17* | NM_011332 | 1.14 | 0.699596 |  | *Il22* | NM_016971 | 1.10 | 0.738362 |
| *Ccl19* | NM_011888 | 0.96 | 0.843475 |  | *Il23a* | NM_031252 | 1.73 | 0.020365 |
| *Ccl2* | NM_011333 | 1.72 | 0.013029 |  | *Il23r* | NM_144548 | 1.04 | 0.959141 |
| *Ccl20* | NM_016960 | 1.06 | 0.896253 |  | *Il5* | NM_010558 | 1.47 | 0.039197 |
| *Ccl22* | NM_009137 | 1.89 | 0.030471 |  | *Il6* | NM_001314054 | 1.12 | 0.449612 |
| *Ccl24* | NM_019577 | 1.89 | 0.007052 |  | *Il6ra* | NM_010559 | 1.44 | 0.049227 |
| *Ccl25* | NM_009138 | 0.85 | 0.423804 |  | *Il7* | NM_008371 | 1.21 | 0.290238 |
| *Ccl3* | NM_011337 | 2.14 | 0.007704 |  | *Il9* | NM_008373 | 0.61 | 0.059705 |
| *Ccl4* | NM_013652 | 1.13 | 0.373647 |  | *Itgb2* | NM_008404 | 3.44 | 0.000115 |
| *Ccl5* | NM_013653 | 3.14 | 0.000649 |  | *Kng1* | NM_023125 | 0.90 | 0.874125 |
| *Ccl7* | NM_013654 | 2.82 | 0.000154 |  | *Lta* | NM_010735 | ND | ND |
| *Ccl8* | NM_021443 | 4.38 | 0.011904 |  | *Ltb* | NM_008518 | 1.49 | 0.087755 |
| *Ccr1* | NM_009912 | 1.52 | 0.038815 |  | *Ly96* | NM_016923 | 1.71 | 0.002333 |
| *Ccr2* | NM_009915 | 3.58 | 0.001103 |  | *Myd88* | NM_010851 | 1.69 | 0.000024 |
| *Ccr3* | NM_009914 | 4.46 | 0.003033 |  | *Nfkb1* | NM_008689 | 1.09 | 0.217142 |
| *Ccr4* | NM_009916 | 1.04 | 0.968004 |  | *Nos2* | NM_001313921 | 1.23 | 0.360367 |
| *Ccr7* | NM_007719 | 1.03 | 0.501981 |  | *Nr3c1* | NM_008173 | 1.01 | 0.723685 |
| *Cd14* | NM_009841 | 2.07 | 0.004797 |  | *Ptgs2* | NM_011198 | 3.41 | 0.000707 |
| *Cd40* | NM_011611 | 1.61 | 0.011946 |  | *Ripk2* | NM_138952 | 1.11 | 0.238342 |
| *Cd40lg* | NM_011616 | 1.38 | 0.099671 |  | *Sele* | NM_011345 | 1.03 | 0.716587 |
| *Cebpb* | NM_009883 | 0.85 | 0.196995 |  | *Tirap* | NM_054096 | 1.19 | 0.361232 |
| *Crp* | NM_007768 | 0.93 | 0.580330 |  | *Tlr1* | NM_030682 | 5.30 | 0.002137 |
| *Csf1* | NM_007778 | 1.62 | 0.003471 |  | *Tlr2* | NM_011905 | 2.89 | 0.000353 |
| *Cxcl1* | NM_008176 | 4.65 | 0.005084 |  | *Tlr3* | NM_126166 | 1.44 | 0.028303 |
| *Cxcl10* | NM_021274 | 1.45 | 0.107195 |  | *Tlr4* | NM_021297 | 1.70 | 0.013220 |
| *Cxcl11* | NM_019494 | 1.62 | 0.384028 |  | *Tlr5* | NM_016928 | 1.79 | 0.003622 |
| *Cxcl2* | NM_009140 | 1.08 | 0.747813 |  | *Tlr6* | NM_011604 | 1.53 | 0.018497 |
| *Cxcl3* | NM_203320 | 1.24 | 0.377114 |  | *Tlr7* | NM_133211 | 2.07 | 0.011924 |
| *Cxcl5* | NM_009141 | 1.57 | 0.282941 |  | *Tlr9* | NM_031178 | 2.29 | 0.000399 |
| *Cxcl9* | NM_008599 | 3.43 | 0.017497 |  | *Tnf* | NM_013693 | 3.51 | 0.001050 |
| *Cxcr1* | NM_178241 | 0.96 | 0.866919 |  | *Tnfsf14* | NM_019418 | 1.07 | 0.687943 |
| *Cxcr2* | NM_009909 | 0.81 | 0.593816 |  | *Tollip* | NM_023764 | 1.07 | 0.272566 |
| *Cxcr4* | NM_009911 | 2.10 | 0.004531 |  | *Actb* | NM_007393 | 1.23 | 0.041719 |
| *Fasl* | NM_010177 | 1.91 | 0.014109 |  | *B2m* | NM_009735 | 1.06 | 0.392514 |
| *Fos* | NM_010234 | 10.44 | 0.000811 |  | *Gapdh* | NM_008084 | 0.93 | 0.856910 |
| *Ifng* | NM_008337 | 1.88 | 0.032287 |  | *Gusb* | NM_010368 | 1.66 | 0.000596 |
| *Il10* | NM_010548 | 1.49 | 0.269354 |  |  |  |  |  |
| *Il10rb* | NM_008349 | 1.41 | 0.051615 |  |  |  |  |  |

**­Supplementary Table 7. Differential mRNA levels of control mice 10 days post 30°/s protocol vs nonexposed muscles.**

Expression which surpassed 2-fold regulation (below 0.5 fold change or above 2 fold change) with a P value < 0.05 was considered differentially expressed. ND, Not detected. Not highlighted – unchanged, Orange – upregulated, Blue - downregulated. Sample sizes were N = 8 per group.
