# Supplementary material for: Transcriptional and morphological responses following distinct muscle contraction protocols for Snell dwarf (Pit1dw/dw ) mice
Source: Physiol Rep. 2024 Sep 3;12(17):e70027. doi: 10.14814/phy2.70027 (PMC11371489; doi:10.14814/phy2.70027)
Supplement: Supplementary file 17 — Table S8. [file PHY2-12-e70027-s001.docx]

|  | RefSeq | 500°/s protocol vs nonexposed | |  |  | RefSeq | 500°/s protocol vs nonexposed | |
| --- | --- | --- | --- | --- | --- | --- | --- | --- |
|  |  |  |  |  |  |  |  |  |
|  |  | Fold change | P value |  |  |  | Fold change | P value |
| *Bcl6* | NM_009744 | 0.89 | 0.788386 |  | *Il17a* | NM_010552 | 0.61 | 0.218040 |
| *C3* | NM_009778 | 0.87 | 0.516247 |  | *Il18* | NM_008360 | 1.43 | 0.013861 |
| *C3ar1* | NM_009779 | 2.55 | 0.003540 |  | *Il1a* | NM_010554 | 0.87 | 0.359326 |
| *C4b* | NM_009780 | 1.48 | 0.233347 |  | *Il1b* | NM_008361 | 0.85 | 0.852219 |
| *Ccl1* | NM_011329 | 0.97 | 0.944911 |  | *Il1r1* | NM_008362 | 1.08 | 0.476535 |
| *Ccl11* | NM_011330 | 0.85 | 0.363278 |  | *Il1rap* | NM_008364 | 1.02 | 0.821154 |
| *Ccl12* | NM_011331 | 3.65 | 0.013712 |  | *Il1rn* | NM_031167 | 1.59 | 0.235506 |
| *Ccl17* | NM_011332 | 1.05 | 0.937973 |  | *Il22* | NM_016971 | 0.57 | 0.099985 |
| *Ccl19* | NM_011888 | 0.93 | 0.975259 |  | *Il23a* | NM_031252 | 0.77 | 0.988683 |
| *Ccl2* | NM_011333 | 1.44 | 0.167887 |  | *Il23r* | NM_144548 | 0.56 | 0.019330 |
| *Ccl20* | NM_016960 | 0.67 | 0.169280 |  | *Il5* | NM_010558 | 1.14 | 0.401912 |
| *Ccl22* | NM_009137 | 1.71 | 0.086272 |  | *Il6* | NM_001314054 | 0.60 | 0.479980 |
| *Ccl24* | NM_019577 | 1.19 | 0.303939 |  | *Il6ra* | NM_010559 | 1.28 | 0.166461 |
| *Ccl25* | NM_009138 | 1.14 | 0.449370 |  | *Il7* | NM_008371 | 0.82 | 0.340751 |
| *Ccl3* | NM_011337 | 1.47 | 0.085273 |  | *Il9* | NM_008373 | 0.56 | 0.064500 |
| *Ccl4* | NM_013652 | 0.95 | 0.915659 |  | *Itgb2* | NM_008404 | 2.31 | 0.004856 |
| *Ccl5* | NM_013653 | 2.86 | 0.005053 |  | *Kng1* | NM_023125 | 0.75 | 0.186083 |
| *Ccl7* | NM_013654 | 2.50 | 0.036091 |  | *Lta* | NM_010735 | ND | ND |
| *Ccl8* | NM_021443 | 4.85 | 0.133833 |  | *Ltb* | NM_008518 | 0.60 | 0.983613 |
| *Ccr1* | NM_009912 | 1.27 | 0.152942 |  | *Ly96* | NM_016923 | 1.41 | 0.025129 |
| *Ccr2* | NM_009915 | 2.49 | 0.001901 |  | *Myd88* | NM_010851 | 1.38 | 0.006568 |
| *Ccr3* | NM_009914 | 3.71 | 0.000476 |  | *Nfkb1* | NM_008689 | 1.24 | 0.003513 |
| *Ccr4* | NM_009916 | 0.78 | 0.629915 |  | *Nos2* | NM_001313921 | 0.77 | 0.814852 |
| *Ccr7* | NM_007719 | 1.12 | 0.425313 |  | *Nr3c1* | NM_008173 | 1.09 | 0.292682 |
| *Cd14* | NM_009841 | 1.90 | 0.014242 |  | *Ptgs2* | NM_011198 | 1.42 | 0.137417 |
| *Cd40* | NM_011611 | 1.24 | 0.188918 |  | *Ripk2* | NM_138952 | 1.06 | 0.535484 |
| *Cd40lg* | NM_011616 | 0.93 | 0.667913 |  | *Sele* | NM_011345 | 0.65 | 0.226609 |
| *Cebpb* | NM_009883 | 0.81 | 0.237369 |  | *Tirap* | NM_054096 | 1.20 | 0.311732 |
| *Crp* | NM_007768 | 0.57 | 0.073458 |  | *Tlr1* | NM_030682 | 5.14 | 0.007895 |
| *Csf1* | NM_007778 | 1.18 | 0.271544 |  | *Tlr2* | NM_011905 | 2.06 | 0.012281 |
| *Cxcl1* | NM_008176 | 1.25 | 0.535288 |  | *Tlr3* | NM_126166 | 1.56 | 0.016336 |
| *Cxcl10* | NM_021274 | 1.74 | 0.035421 |  | *Tlr4* | NM_021297 | 1.55 | 0.038378 |
| *Cxcl11* | NM_019494 | 1.07 | 0.731151 |  | *Tlr5* | NM_016928 | 1.87 | 0.011615 |
| *Cxcl2* | NM_009140 | 0.65 | 0.233966 |  | *Tlr6* | NM_011604 | 1.64 | 0.003080 |
| *Cxcl3* | NM_203320 | 0.83 | 0.990511 |  | *Tlr7* | NM_133211 | 2.21 | 0.021189 |
| *Cxcl5* | NM_009141 | 1.33 | 0.418913 |  | *Tlr9* | NM_031178 | 1.94 | 0.003327 |
| *Cxcl9* | NM_008599 | 4.32 | 0.025772 |  | *Tnf* | NM_013693 | 1.63 | 0.219352 |
| *Cxcr1* | NM_178241 | 0.78 | 0.319550 |  | *Tnfsf14* | NM_019418 | 0.72 | 0.376825 |
| *Cxcr2* | NM_009909 | 0.73 | 0.223192 |  | *Tollip* | NM_023764 | 1.00 | 0.958036 |
| *Cxcr4* | NM_009911 | 1.87 | 0.045456 |  | *Actb* | NM_007393 | 1.22 | 0.032832 |
| *Fasl* | NM_010177 | 1.19 | 0.150178 |  | *B2m* | NM_009735 | 1.07 | 0.564318 |
| *Fos* | NM_010234 | 2.50 | 0.009297 |  | *Gapdh* | NM_008084 | 1.12 | 0.355971 |
| *Ifng* | NM_008337 | 1.77 | 0.131739 |  | *Gusb* | NM_010368 | 1.38 | 0.015405 |
| *Il10* | NM_010548 | 1.49 | 0.255794 |  |  |  |  |  |
| *Il10rb* | NM_008349 | 1.21 | 0.160893 |  |  |  |  |  |

**­Supplementary Table 8. Differential mRNA levels of control mice 10 days post 500°/s protocol vs nonexposed muscles.**

Expression which surpassed 2-fold regulation (below 0.5 fold change or above 2 fold change) with a P value < 0.05 was considered differentially expressed. ND, Not detected. Not highlighted – unchanged, Orange – upregulated, Blue - downregulated. Sample sizes were N = 8 per group.
