# Supplementary material for: Transcriptional and morphological responses following distinct muscle contraction protocols for Snell dwarf (Pit1dw/dw ) mice
Source: Physiol Rep. 2024 Sep 3;12(17):e70027. doi: 10.14814/phy2.70027 (PMC11371489; doi:10.14814/phy2.70027)
Supplement: Supplementary file 18 — Table S9. [file PHY2-12-e70027-s015.docx]

|  | RefSeq | 500°/s protocol vs  30°/s protocol | |  |  | RefSeq | 500°/s protocol vs  30°/s protocol | |
| --- | --- | --- | --- | --- | --- | --- | --- | --- |
|  |  |  |  |  |  |  |  |  |
|  |  | Fold change | P value |  |  |  | Fold change | P value |
| *Bcl6* | NM_009744 | 1.12 | 0.538719 |  | *Il17a* | NM_010552 | 0.56 | 0.165814 |
| *C3* | NM_009778 | 0.81 | 0.428842 |  | *Il18* | NM_008360 | 0.84 | 0.283431 |
| *C3ar1* | NM_009779 | 0.74 | 0.411499 |  | *Il1a* | NM_010554 | 0.89 | 0.371620 |
| *C4b* | NM_009780 | 0.92 | 0.913785 |  | *Il1b* | NM_008361 | 0.27 | 0.003166 |
| *Ccl1* | NM_011329 | 0.62 | 0.116577 |  | *Il1r1* | NM_008362 | 0.88 | 0.427964 |
| *Ccl11* | NM_011330 | 1.18 | 0.456645 |  | *Il1rap* | NM_008364 | 0.89 | 0.279977 |
| *Ccl12* | NM_011331 | 2.47 | 0.035315 |  | *Il1rn* | NM_031167 | 0.57 | 0.053001 |
| *Ccl17* | NM_011332 | 0.92 | 0.736341 |  | *Il22* | NM_016971 | 0.52 | 0.063305 |
| *Ccl19* | NM_011888 | 0.96 | 0.869461 |  | *Il23a* | NM_031252 | 0.44 | 0.082084 |
| *Ccl2* | NM_011333 | 0.84 | 0.796597 |  | *Il23r* | NM_144548 | 0.54 | 0.002853 |
| *Ccl20* | NM_016960 | 0.63 | 0.084370 |  | *Il5* | NM_010558 | 0.78 | 0.475017 |
| *Ccl22* | NM_009137 | 0.90 | 0.766665 |  | *Il6* | NM_001314054 | 0.53 | 0.243164 |
| *Ccl24* | NM_019577 | 0.63 | 0.075628 |  | *Il6ra* | NM_010559 | 0.89 | 0.398892 |
| *Ccl25* | NM_009138 | 1.34 | 0.130781 |  | *Il7* | NM_008371 | 0.67 | 0.042178 |
| *Ccl3* | NM_011337 | 0.69 | 0.212663 |  | *Il9* | NM_008373 | 0.93 | 0.963742 |
| *Ccl4* | NM_013652 | 0.84 | 0.630260 |  | *Itgb2* | NM_008404 | 0.67 | 0.143082 |
| *Ccl5* | NM_013653 | 0.91 | 0.933803 |  | *Kng1* | NM_023125 | 0.83 | 0.269239 |
| *Ccl7* | NM_013654 | 0.89 | 0.891634 |  | *Lta* | NM_010735 | ND | ND |
| *Ccl8* | NM_021443 | 1.11 | 0.433989 |  | *Ltb* | NM_008518 | 0.40 | 0.238624 |
| *Ccr1* | NM_009912 | 0.83 | 0.467115 |  | *Ly96* | NM_016923 | 0.82 | 0.208627 |
| *Ccr2* | NM_009915 | 0.69 | 0.127608 |  | *Myd88* | NM_010851 | 0.82 | 0.083442 |
| *Ccr3* | NM_009914 | 0.83 | 0.282795 |  | *Nfkb1* | NM_008689 | 1.14 | 0.554250 |
| *Ccr4* | NM_009916 | 0.75 | 0.595296 |  | *Nos2* | NM_001313921 | 0.62 | 0.354832 |
| *Ccr7* | NM_007719 | 1.09 | 0.981094 |  | *Nr3c1* | NM_008173 | 1.08 | 0.753076 |
| *Cd14* | NM_009841 | 0.92 | 0.709621 |  | *Ptgs2* | NM_011198 | 0.42 | 0.005501 |
| *Cd40* | NM_011611 | 0.77 | 0.332982 |  | *Ripk2* | NM_138952 | 0.96 | 0.518465 |
| *Cd40lg* | NM_011616 | 0.67 | 0.090423 |  | *Sele* | NM_011345 | 0.63 | 0.064908 |
| *Cebpb* | NM_009883 | 0.96 | 0.905081 |  | *Tirap* | NM_054096 | 1.01 | 0.841037 |
| *Crp* | NM_007768 | 0.61 | 0.103209 |  | *Tlr1* | NM_030682 | 0.97 | 0.747914 |
| *Csf1* | NM_007778 | 0.73 | 0.037443 |  | *Tlr2* | NM_011905 | 0.72 | 0.264609 |
| *Cxcl1* | NM_008176 | 0.27 | 0.007390 |  | *Tlr3* | NM_126166 | 1.08 | 0.926707 |
| *Cxcl10* | NM_021274 | 1.19 | 0.207603 |  | *Tlr4* | NM_021297 | 0.91 | 0.595028 |
| *Cxcl11* | NM_019494 | 0.66 | 0.167622 |  | *Tlr5* | NM_016928 | 1.04 | 0.829556 |
| *Cxcl2* | NM_009140 | 0.60 | 0.142122 |  | *Tlr6* | NM_011604 | 1.07 | 0.813585 |
| *Cxcl3* | NM_203320 | 0.67 | 0.559695 |  | *Tlr7* | NM_133211 | 1.07 | 0.917508 |
| *Cxcl5* | NM_009141 | 0.85 | 0.966257 |  | *Tlr9* | NM_031178 | 0.85 | 0.471997 |
| *Cxcl9* | NM_008599 | 1.26 | 0.472160 |  | *Tnf* | NM_013693 | 0.46 | 0.023614 |
| *Cxcr1* | NM_178241 | 0.81 | 0.283173 |  | *Tnfsf14* | NM_019418 | 0.68 | 0.251726 |
| *Cxcr2* | NM_009909 | 0.90 | 0.447553 |  | *Tollip* | NM_023764 | 0.93 | 0.267973 |
| *Cxcr4* | NM_009911 | 0.89 | 0.717217 |  | *Actb* | NM_007393 | 0.99 | 0.767474 |
| *Fasl* | NM_010177 | 0.62 | 0.048525 |  | *B2m* | NM_009735 | 1.01 | 0.743152 |
| *Fos* | NM_010234 | 0.24 | 0.003946 |  | *Gapdh* | NM_008084 | 1.21 | 0.306600 |
| *Ifng* | NM_008337 | 0.94 | 0.787045 |  | *Gusb* | NM_010368 | 0.83 | 0.166630 |
| *Il10* | NM_010548 | 1.00 | 0.900194 |  |  |  |  |  |
| *Il10rb* | NM_008349 | 0.86 | 0.358028 |  |  |  |  |  |

**­Supplementary Table 9. Differential mRNA levels of muscles of control mice 10 days post 500°/s protocol vs 30°/s protocol.**

Expression which surpassed 2-fold regulation (below 0.5 fold change or above 2 fold change) with a P value < 0.05 was considered differentially expressed. ND, Not detected. Not highlighted – unchanged, Orange – upregulated, Blue - downregulated. Sample sizes were N = 8 per group.
