# Supplementary material for: Transcriptional and morphological responses following distinct muscle contraction protocols for Snell dwarf (Pit1dw/dw ) mice
Source: Physiol Rep. 2024 Sep 3;12(17):e70027. doi: 10.14814/phy2.70027 (PMC11371489; doi:10.14814/phy2.70027)
Supplement: Supplementary file 19 — Table S10. [file PHY2-12-e70027-s022.docx]

|  | RefSeq | 30°/s protocol vs nonexposed | |  |  | RefSeq | 30°/s protocol vs nonexposed | |
| --- | --- | --- | --- | --- | --- | --- | --- | --- |
|  |  |  |  |  |  |  |  |  |
|  |  | Fold change | P value |  |  |  | Fold change | P value |
| *Bcl6* | NM_009744 | 0.44 | 0.000000 |  | *Il17a* | NM_010552 | 0.42 | 0.015465 |
| *C3* | NM_009778 | 1.20 | 0.341838 |  | *Il18* | NM_008360 | 8.20 | 0.000000 |
| *C3ar1* | NM_009779 | 64.79 | 0.000000 |  | *Il1a* | NM_010554 | 0.49 | 0.000631 |
| *C4b* | NM_009780 | 1.06 | 0.838771 |  | *Il1b* | NM_008361 | 3.57 | 0.000881 |
| *Ccl1* | NM_011329 | 0.70 | 0.254225 |  | *Il1r1* | NM_008362 | 2.70 | 0.000016 |
| *Ccl11* | NM_011330 | 0.49 | 0.000025 |  | *Il1rap* | NM_008364 | 1.34 | 0.026317 |
| *Ccl12* | NM_011331 | 11.81 | 0.000073 |  | *Il1rn* | NM_031167 | 17.06 | 0.000002 |
| *Ccl17* | NM_011332 | 0.95 | 0.788273 |  | *Il22* | NM_016971 | 0.31 | 0.068463 |
| *Ccl19* | NM_011888 | 0.67 | 0.016848 |  | *Il23a* | NM_031252 | 0.83 | 0.859740 |
| *Ccl2* | NM_011333 | 26.61 | 0.000095 |  | *Il23r* | NM_144548 | 0.66 | 0.645096 |
| *Ccl20* | NM_016960 | 1.70 | 0.136791 |  | *Il5* | NM_010558 | 0.39 | 0.000091 |
| *Ccl22* | NM_009137 | 0.95 | 0.908555 |  | *Il6* | NM_001314054 | 1.99 | 0.023929 |
| *Ccl24* | NM_019577 | 1.65 | 0.043984 |  | *Il6ra* | NM_010559 | 4.92 | 0.000000 |
| *Ccl25* | NM_009138 | 0.65 | 0.001156 |  | *Il7* | NM_008371 | 0.50 | 0.003265 |
| *Ccl3* | NM_011337 | 10.89 | 0.000200 |  | *Il9* | NM_008373 | 0.49 | 0.061759 |
| *Ccl4* | NM_013652 | 1.86 | 0.005757 |  | *Itgb2* | NM_008404 | 37.61 | 0.000000 |
| *Ccl5* | NM_013653 | 1.62 | 0.014022 |  | *Kng1* | NM_023125 | 0.42 | 0.033330 |
| *Ccl7* | NM_013654 | 35.83 | 0.000073 |  | *Lta* | NM_010735 | ND | ND |
| *Ccl8* | NM_021443 | 75.60 | 0.000125 |  | *Ltb* | NM_008518 | 0.70 | 0.078995 |
| *Ccr1* | NM_009912 | 21.40 | 0.000000 |  | *Ly96* | NM_016923 | 3.76 | 0.000000 |
| *Ccr2* | NM_009915 | 23.34 | 0.000000 |  | *Myd88* | NM_010851 | 4.74 | 0.000000 |
| *Ccr3* | NM_009914 | 74.20 | 0.000006 |  | *Nfkb1* | NM_008689 | 1.34 | 0.001358 |
| *Ccr4* | NM_009916 | 0.36 | 0.001579 |  | *Nos2* | NM_001313921 | 0.56 | 0.110530 |
| *Ccr7* | NM_007719 | 1.76 | 0.024255 |  | *Nr3c1* | NM_008173 | 0.48 | 0.000001 |
| *Cd14* | NM_009841 | 19.87 | 0.000000 |  | *Ptgs2* | NM_011198 | 2.42 | 0.001071 |
| *Cd40* | NM_011611 | 5.33 | 0.000003 |  | *Ripk2* | NM_138952 | 1.30 | 0.048083 |
| *Cd40lg* | NM_011616 | 0.53 | 0.046124 |  | *Sele* | NM_011345 | 0.58 | 0.008837 |
| *Cebpb* | NM_009883 | 0.66 | 0.002472 |  | *Tirap* | NM_054096 | 0.61 | 0.004319 |
| *Crp* | NM_007768 | 0.37 | 0.028168 |  | *Tlr1* | NM_030682 | 34.90 | 0.000000 |
| *Csf1* | NM_007778 | 3.84 | 0.000047 |  | *Tlr2* | NM_011905 | 7.84 | 0.000675 |
| *Cxcl1* | NM_008176 | 3.62 | 0.004844 |  | *Tlr3* | NM_126166 | 1.56 | 0.005410 |
| *Cxcl10* | NM_021274 | 1.41 | 0.149233 |  | *Tlr4* | NM_021297 | 2.25 | 0.000005 |
| *Cxcl11* | NM_019494 | 0.65 | 0.020017 |  | *Tlr5* | NM_016928 | 3.30 | 0.000000 |
| *Cxcl2* | NM_009140 | 0.41 | 0.000326 |  | *Tlr6* | NM_011604 | 6.91 | 0.000000 |
| *Cxcl3* | NM_203320 | 1.18 | 0.304792 |  | *Tlr7* | NM_133211 | 22.41 | 0.000000 |
| *Cxcl5* | NM_009141 | 34.52 | 0.000879 |  | *Tlr9* | NM_031178 | 6.14 | 0.000000 |
| *Cxcl9* | NM_008599 | 2.54 | 0.013458 |  | *Tnf* | NM_013693 | 8.59 | 0.000311 |
| *Cxcr1* | NM_178241 | 0.44 | 0.000113 |  | *Tnfsf14* | NM_019418 | 1.29 | 0.093445 |
| *Cxcr2* | NM_009909 | 1.95 | 0.068189 |  | *Tollip* | NM_023764 | 0.77 | 0.006875 |
| *Cxcr4* | NM_009911 | 4.76 | 0.000000 |  | *Actb* | NM_007393 | 3.66 | 0.000000 |
| *Fasl* | NM_010177 | 1.07 | 0.442511 |  | *B2m* | NM_009735 | 2.66 | 0.000002 |
| *Fos* | NM_010234 | 1.95 | 0.002831 |  | *Gapdh* | NM_008084 | 0.24 | 0.000001 |
| *Ifng* | NM_008337 | 0.93 | 0.919838 |  | *Gusb* | NM_010368 | 8.27 | 0.000000 |
| *Il10* | NM_010548 | 4.83 | 0.000088 |  |  |  |  |  |
| *Il10rb* | NM_008349 | 4.28 | 0.000000 |  |  |  |  |  |

**­Supplementary Table 10. Differential mRNA levels of Snell dwarf mice 3 days post 30°/s protocol vs nonexposed muscles.**

Expression which surpassed 2-fold regulation (below 0.5 fold change or above 2 fold change) with a P value < 0.05 was considered differentially expressed. ND, Not detected. Not highlighted – unchanged, Orange – upregulated, Blue - downregulated. Sample sizes were N = 8 per group.
