# Supplementary material for: Transcriptional and morphological responses following distinct muscle contraction protocols for Snell dwarf (Pit1dw/dw ) mice
Source: Physiol Rep. 2024 Sep 3;12(17):e70027. doi: 10.14814/phy2.70027 (PMC11371489; doi:10.14814/phy2.70027)
Supplement: Supplementary file 20 — Table S11. [file PHY2-12-e70027-s008.docx]

|  | RefSeq | 500°/s protocol vs nonexposed | |  |  | RefSeq | 500°/s protocol vs nonexposed | |
| --- | --- | --- | --- | --- | --- | --- | --- | --- |
|  |  |  |  |  |  |  |  |  |
|  |  | Fold change | P value |  |  |  | Fold change | P value |
| *Bcl6* | NM_009744 | 0.43 | 0.000000 |  | *Il17a* | NM_010552 | 0.25 | 0.000027 |
| *C3* | NM_009778 | 1.35 | 0.117986 |  | *Il18* | NM_008360 | 7.18 | 0.000000 |
| *C3ar1* | NM_009779 | 66.79 | 0.000000 |  | *Il1a* | NM_010554 | 0.54 | 0.000122 |
| *C4b* | NM_009780 | 1.09 | 0.912369 |  | *Il1b* | NM_008361 | 4.28 | 0.001006 |
| *Ccl1* | NM_011329 | 0.45 | 0.051292 |  | *Il1r1* | NM_008362 | 2.33 | 0.000015 |
| *Ccl11* | NM_011330 | 0.58 | 0.000132 |  | *Il1rap* | NM_008364 | 1.36 | 0.010245 |
| *Ccl12* | NM_011331 | 13.25 | 0.000000 |  | *Il1rn* | NM_031167 | 18.78 | 0.000000 |
| *Ccl17* | NM_011332 | 1.66 | 0.039520 |  | *Il22* | NM_016971 | 0.27 | 0.005997 |
| *Ccl19* | NM_011888 | 0.68 | 0.018962 |  | *Il23a* | NM_031252 | 0.76 | 0.037846 |
| *Ccl2* | NM_011333 | 29.13 | 0.000000 |  | *Il23r* | NM_144548 | 0.55 | 0.177451 |
| *Ccl20* | NM_016960 | 2.01 | 0.017365 |  | *Il5* | NM_010558 | 0.46 | 0.000055 |
| *Ccl22* | NM_009137 | 1.32 | 0.187582 |  | *Il6* | NM_001314054 | 1.40 | 0.150260 |
| *Ccl24* | NM_019577 | 1.51 | 0.114709 |  | *Il6ra* | NM_010559 | 4.18 | 0.000000 |
| *Ccl25* | NM_009138 | 0.70 | 0.000791 |  | *Il7* | NM_008371 | 0.50 | 0.002625 |
| *Ccl3* | NM_011337 | 13.26 | 0.000000 |  | *Il9* | NM_008373 | 0.76 | 0.357165 |
| *Ccl4* | NM_013652 | 2.00 | 0.000049 |  | *Itgb2* | NM_008404 | 36.78 | 0.000000 |
| *Ccl5* | NM_013653 | 1.53 | 0.009759 |  | *Kng1* | NM_023125 | 0.38 | 0.013630 |
| *Ccl7* | NM_013654 | 33.50 | 0.000000 |  | *Lta* | NM_010735 | ND | ND |
| *Ccl8* | NM_021443 | 79.79 | 0.000018 |  | *Ltb* | NM_008518 | 0.75 | 0.101376 |
| *Ccr1* | NM_009912 | 23.81 | 0.000000 |  | *Ly96* | NM_016923 | 3.61 | 0.000000 |
| *Ccr2* | NM_009915 | 23.54 | 0.000000 |  | *Myd88* | NM_010851 | 4.79 | 0.000000 |
| *Ccr3* | NM_009914 | 68.93 | 0.000001 |  | *Nfkb1* | NM_008689 | 1.28 | 0.001082 |
| *Ccr4* | NM_009916 | 0.50 | 0.001194 |  | *Nos2* | NM_001313921 | 0.62 | 0.086795 |
| *Ccr7* | NM_007719 | 2.05 | 0.008926 |  | *Nr3c1* | NM_008173 | 0.50 | 0.000000 |
| *Cd14* | NM_009841 | 18.49 | 0.000000 |  | *Ptgs2* | NM_011198 | 2.61 | 0.000029 |
| *Cd40* | NM_011611 | 5.97 | 0.000000 |  | *Ripk2* | NM_138952 | 1.43 | 0.004521 |
| *Cd40lg* | NM_011616 | 0.38 | 0.001186 |  | *Sele* | NM_011345 | 0.56 | 0.001771 |
| *Cebpb* | NM_009883 | 0.68 | 0.000041 |  | *Tirap* | NM_054096 | 0.58 | 0.001293 |
| *Crp* | NM_007768 | 0.44 | 0.004570 |  | *Tlr1* | NM_030682 | 35.69 | 0.000000 |
| *Csf1* | NM_007778 | 3.33 | 0.000000 |  | *Tlr2* | NM_011905 | 7.35 | 0.000481 |
| *Cxcl1* | NM_008176 | 4.67 | 0.000011 |  | *Tlr3* | NM_126166 | 1.59 | 0.000885 |
| *Cxcl10* | NM_021274 | 1.74 | 0.037262 |  | *Tlr4* | NM_021297 | 2.22 | 0.000000 |
| *Cxcl11* | NM_019494 | 0.60 | 0.005390 |  | *Tlr5* | NM_016928 | 3.06 | 0.000000 |
| *Cxcl2* | NM_009140 | 0.78 | 0.676344 |  | *Tlr6* | NM_011604 | 6.95 | 0.000000 |
| *Cxcl3* | NM_203320 | 2.48 | 0.036763 |  | *Tlr7* | NM_133211 | 23.76 | 0.000000 |
| *Cxcl5* | NM_009141 | 46.94 | 0.000044 |  | *Tlr9* | NM_031178 | 5.83 | 0.000000 |
| *Cxcl9* | NM_008599 | 1.57 | 0.134057 |  | *Tnf* | NM_013693 | 8.68 | 0.000001 |
| *Cxcr1* | NM_178241 | 0.59 | 0.068622 |  | *Tnfsf14* | NM_019418 | 1.45 | 0.001210 |
| *Cxcr2* | NM_009909 | 2.44 | 0.028716 |  | *Tollip* | NM_023764 | 0.75 | 0.000465 |
| *Cxcr4* | NM_009911 | 4.23 | 0.000000 |  | *Actb* | NM_007393 | 3.54 | 0.000000 |
| *Fasl* | NM_010177 | 0.53 | 0.020808 |  | *B2m* | NM_009735 | 2.77 | 0.000003 |
| *Fos* | NM_010234 | 1.45 | 0.060632 |  | *Gapdh* | NM_008084 | 0.28 | 0.000000 |
| *Ifng* | NM_008337 | 0.37 | 0.054891 |  | *Gusb* | NM_010368 | 7.52 | 0.000000 |
| *Il10* | NM_010548 | 4.44 | 0.000010 |  |  |  |  |  |
| *Il10rb* | NM_008349 | 3.85 | 0.000000 |  |  |  |  |  |

**­Supplementary Table 11. Differential mRNA levels of Snell dwarf mice 3 days post 500°/s protocol vs nonexposed muscles.**

Expression which surpassed 2-fold regulation (below 0.5 fold change or above 2 fold change) with a P value < 0.05 was considered differentially expressed. ND, Not detected. Not highlighted – unchanged, Orange – upregulated, Blue - downregulated. Sample sizes were N = 8 per group.
