# Supplementary material for: Transcriptional and morphological responses following distinct muscle contraction protocols for Snell dwarf (Pit1dw/dw ) mice
Source: Physiol Rep. 2024 Sep 3;12(17):e70027. doi: 10.14814/phy2.70027 (PMC11371489; doi:10.14814/phy2.70027)
Supplement: Supplementary file 21 — Table S12. [file PHY2-12-e70027-s007.docx]

|  | RefSeq | 500°/s protocol vs  30°/s protocol | |  |  | RefSeq | 500°/s protocol vs  30°/s protocol | |
| --- | --- | --- | --- | --- | --- | --- | --- | --- |
|  |  |  |  |  |  |  |  |  |
|  |  | Fold change | P value |  |  |  | Fold change | P value |
| *Bcl6* | NM_009744 | 0.88 | 0.127009 |  | *Il17a* | NM_010552 | 0.56 | 0.189519 |
| *C3* | NM_009778 | 1.03 | 0.833911 |  | *Il18* | NM_008360 | 0.93 | 0.342017 |
| *C3ar1* | NM_009779 | 1.35 | 0.688003 |  | *Il1a* | NM_010554 | 0.99 | 0.820593 |
| *C4b* | NM_009780 | 0.93 | 0.594777 |  | *Il1b* | NM_008361 | 1.32 | 0.293718 |
| *Ccl1* | NM_011329 | 0.58 | 0.228890 |  | *Il1r1* | NM_008362 | 0.95 | 0.513432 |
| *Ccl11* | NM_011330 | 1.04 | 0.989161 |  | *Il1rap* | NM_008364 | 1.01 | 0.998226 |
| *Ccl12* | NM_011331 | 1.14 | 0.661985 |  | *Il1rn* | NM_031167 | 1.42 | 0.464510 |
| *Ccl17* | NM_011332 | 1.45 | 0.071697 |  | *Il22* | NM_016971 | 0.73 | 0.215999 |
| *Ccl19* | NM_011888 | 0.95 | 0.728841 |  | *Il23a* | NM_031252 | 0.77 | 0.193264 |
| *Ccl2* | NM_011333 | 1.55 | 0.734710 |  | *Il23r* | NM_144548 | 0.80 | 0.512252 |
| *Ccl20* | NM_016960 | 1.33 | 0.485490 |  | *Il5* | NM_010558 | 0.93 | 0.673918 |
| *Ccl22* | NM_009137 | 1.26 | 0.243526 |  | *Il6* | NM_001314054 | 0.73 | 0.159810 |
| *Ccl24* | NM_019577 | 0.91 | 0.413356 |  | *Il6ra* | NM_010559 | 0.99 | 0.429381 |
| *Ccl25* | NM_009138 | 0.98 | 0.686700 |  | *Il7* | NM_008371 | 0.98 | 0.917508 |
| *Ccl3* | NM_011337 | 1.38 | 0.460872 |  | *Il9* | NM_008373 | 1.41 | 0.260921 |
| *Ccl4* | NM_013652 | 1.15 | 0.702370 |  | *Itgb2* | NM_008404 | 1.27 | 0.713270 |
| *Ccl5* | NM_013653 | 0.76 | 0.203506 |  | *Kng1* | NM_023125 | 0.80 | 0.265875 |
| *Ccl7* | NM_013654 | 1.29 | 0.770764 |  | *Lta* | NM_010735 | ND | ND |
| *Ccl8* | NM_021443 | 1.04 | 0.997893 |  | *Ltb* | NM_008518 | 0.91 | 0.532359 |
| *Ccr1* | NM_009912 | 1.57 | 0.251454 |  | *Ly96* | NM_016923 | 1.02 | 0.840982 |
| *Ccr2* | NM_009915 | 1.23 | 0.634181 |  | *Myd88* | NM_010851 | 1.12 | 0.560838 |
| *Ccr3* | NM_009914 | 1.14 | 0.827490 |  | *Nfkb1* | NM_008689 | 0.96 | 0.479622 |
| *Ccr4* | NM_009916 | 1.21 | 0.710258 |  | *Nos2* | NM_001313921 | 0.98 | 0.933081 |
| *Ccr7* | NM_007719 | 1.03 | 0.728050 |  | *Nr3c1* | NM_008173 | 0.92 | 0.398542 |
| *Cd14* | NM_009841 | 1.13 | 0.958351 |  | *Ptgs2* | NM_011198 | 1.15 | 0.668031 |
| *Cd40* | NM_011611 | 1.14 | 0.370815 |  | *Ripk2* | NM_138952 | 1.11 | 0.372937 |
| *Cd40lg* | NM_011616 | 0.66 | 0.112505 |  | *Sele* | NM_011345 | 0.92 | 0.427389 |
| *Cebpb* | NM_009883 | 0.99 | 0.669573 |  | *Tirap* | NM_054096 | 0.89 | 0.145815 |
| *Crp* | NM_007768 | 0.89 | 0.280920 |  | *Tlr1* | NM_030682 | 1.27 | 0.530457 |
| *Csf1* | NM_007778 | 0.95 | 0.428396 |  | *Tlr2* | NM_011905 | 1.04 | 0.815546 |
| *Cxcl1* | NM_008176 | 1.46 | 0.604471 |  | *Tlr3* | NM_126166 | 0.93 | 0.489974 |
| *Cxcl10* | NM_021274 | 1.17 | 0.372886 |  | *Tlr4* | NM_021297 | 1.02 | 0.965024 |
| *Cxcl11* | NM_019494 | 0.81 | 0.227368 |  | *Tlr5* | NM_016928 | 0.89 | 0.088249 |
| *Cxcl2* | NM_009140 | 1.81 | 0.044143 |  | *Tlr6* | NM_011604 | 1.20 | 0.607475 |
| *Cxcl3* | NM_203320 | 2.21 | 0.054083 |  | *Tlr7* | NM_133211 | 1.23 | 0.406931 |
| *Cxcl5* | NM_009141 | 2.20 | 0.631765 |  | *Tlr9* | NM_031178 | 1.00 | 0.772354 |
| *Cxcl9* | NM_008599 | 0.46 | 0.129846 |  | *Tnf* | NM_013693 | 1.01 | 0.751207 |
| *Cxcr1* | NM_178241 | 1.18 | 0.347730 |  | *Tnfsf14* | NM_019418 | 1.20 | 0.436517 |
| *Cxcr2* | NM_009909 | 1.45 | 0.412236 |  | *Tollip* | NM_023764 | 0.93 | 0.168125 |
| *Cxcr4* | NM_009911 | 1.03 | 0.726754 |  | *Actb* | NM_007393 | 1.10 | 0.686684 |
| *Fasl* | NM_010177 | 0.42 | 0.027147 |  | *B2m* | NM_009735 | 1.03 | 0.726876 |
| *Fos* | NM_010234 | 0.66 | 0.031968 |  | *Gapdh* | NM_008084 | 0.95 | 0.500604 |
| *Ifng* | NM_008337 | 0.29 | 0.008576 |  | *Gusb* | NM_010368 | 1.11 | 0.875961 |
| *Il10* | NM_010548 | 0.91 | 0.483421 |  |  |  |  |  |
| *Il10rb* | NM_008349 | 0.99 | 0.756074 |  |  |  |  |  |

**­Supplementary Table 12. Differential mRNA levels of muscles of Snell dwarf mice 3 days post 500°/s protocol vs 30°/s protocol.**

Expression which surpassed 2-fold regulation (below 0.5 fold change or above 2 fold change) with a P value < 0.05 was considered differentially expressed. ND, Not detected. Not highlighted – unchanged, Orange – upregulated, Blue - downregulated. Sample sizes were N = 8 per group.
