# Supplementary material for: Transcriptional and morphological responses following distinct muscle contraction protocols for Snell dwarf (Pit1dw/dw ) mice
Source: Physiol Rep. 2024 Sep 3;12(17):e70027. doi: 10.14814/phy2.70027 (PMC11371489; doi:10.14814/phy2.70027)
Supplement: Supplementary file 22 — Table S13. [file PHY2-12-e70027-s014.docx]

|  | RefSeq | 30°/s protocol vs nonexposed | |  |  | RefSeq | 30°/s protocol vs nonexposed | |
| --- | --- | --- | --- | --- | --- | --- | --- | --- |
|  |  |  |  |  |  |  |  |  |
|  |  | Fold change | P value |  |  |  | Fold change | P value |
| *Bcl6* | NM_009744 | 0.58 | 0.035169 |  | *Il17a* | NM_010552 | 0.63 | 0.010761 |
| *C3* | NM_009778 | 1.05 | 0.767774 |  | *Il18* | NM_008360 | 2.98 | 0.000892 |
| *C3ar1* | NM_009779 | 6.99 | 0.000091 |  | *Il1a* | NM_010554 | 0.70 | 0.020513 |
| *C4b* | NM_009780 | 1.29 | 0.297408 |  | *Il1b* | NM_008361 | 1.30 | 0.410076 |
| *Ccl1* | NM_011329 | 1.06 | 0.953739 |  | *Il1r1* | NM_008362 | 1.04 | 0.760442 |
| *Ccl11* | NM_011330 | 0.98 | 0.986400 |  | *Il1rap* | NM_008364 | 1.20 | 0.200406 |
| *Ccl12* | NM_011331 | 4.75 | 0.009930 |  | *Il1rn* | NM_031167 | 1.51 | 0.178059 |
| *Ccl17* | NM_011332 | 1.10 | 0.569582 |  | *Il22* | NM_016971 | 0.68 | 0.123634 |
| *Ccl19* | NM_011888 | 0.95 | 0.596525 |  | *Il23a* | NM_031252 | 1.18 | 0.135350 |
| *Ccl2* | NM_011333 | 0.82 | 0.629479 |  | *Il23r* | NM_144548 | 0.70 | 0.112696 |
| *Ccl20* | NM_016960 | 0.64 | 0.109469 |  | *Il5* | NM_010558 | 0.81 | 0.061216 |
| *Ccl22* | NM_009137 | 0.77 | 0.659509 |  | *Il6* | NM_001314054 | 1.26 | 0.326170 |
| *Ccl24* | NM_019577 | 0.90 | 0.567350 |  | *Il6ra* | NM_010559 | 1.34 | 0.040057 |
| *Ccl25* | NM_009138 | 1.07 | 0.422954 |  | *Il7* | NM_008371 | 0.92 | 0.586494 |
| *Ccl3* | NM_011337 | 2.62 | 0.002464 |  | *Il9* | NM_008373 | 0.57 | 0.025349 |
| *Ccl4* | NM_013652 | 0.99 | 0.769948 |  | *Itgb2* | NM_008404 | 4.04 | 0.002704 |
| *Ccl5* | NM_013653 | 4.09 | 0.000058 |  | *Kng1* | NM_023125 | 0.66 | 0.110143 |
| *Ccl7* | NM_013654 | 1.90 | 0.067739 |  | *Lta* | NM_010735 | ND | ND |
| *Ccl8* | NM_021443 | 22.93 | 0.000676 |  | *Ltb* | NM_008518 | 1.29 | 0.260753 |
| *Ccr1* | NM_009912 | 1.43 | 0.087292 |  | *Ly96* | NM_016923 | 1.71 | 0.000943 |
| *Ccr2* | NM_009915 | 3.67 | 0.000248 |  | *Myd88* | NM_010851 | 1.79 | 0.003336 |
| *Ccr3* | NM_009914 | 9.97 | 0.000784 |  | *Nfkb1* | NM_008689 | 1.10 | 0.208122 |
| *Ccr4* | NM_009916 | 0.61 | 0.002100 |  | *Nos2* | NM_001313921 | 0.98 | 0.745022 |
| *Ccr7* | NM_007719 | 1.26 | 0.479145 |  | *Nr3c1* | NM_008173 | 0.99 | 0.956230 |
| *Cd14* | NM_009841 | 3.94 | 0.000048 |  | *Ptgs2* | NM_011198 | 1.31 | 0.186301 |
| *Cd40* | NM_011611 | 2.90 | 0.000485 |  | *Ripk2* | NM_138952 | 1.09 | 0.498857 |
| *Cd40lg* | NM_011616 | 0.85 | 0.595939 |  | *Sele* | NM_011345 | 0.64 | 0.022444 |
| *Cebpb* | NM_009883 | 0.68 | 0.001435 |  | *Tirap* | NM_054096 | 0.82 | 0.265405 |
| *Crp* | NM_007768 | 1.63 | 0.071563 |  | *Tlr1* | NM_030682 | 4.04 | 0.002236 |
| *Csf1* | NM_007778 | 1.50 | 0.020704 |  | *Tlr2* | NM_011905 | 3.14 | 0.240601 |
| *Cxcl1* | NM_008176 | 1.55 | 0.094817 |  | *Tlr3* | NM_126166 | 1.71 | 0.012500 |
| *Cxcl10* | NM_021274 | 1.49 | 0.201078 |  | *Tlr4* | NM_021297 | 1.49 | 0.014391 |
| *Cxcl11* | NM_019494 | 1.16 | 0.296503 |  | *Tlr5* | NM_016928 | 3.90 | 0.000087 |
| *Cxcl2* | NM_009140 | 0.51 | 0.003485 |  | *Tlr6* | NM_011604 | 2.36 | 0.000251 |
| *Cxcl3* | NM_203320 | 0.80 | 0.578441 |  | *Tlr7* | NM_133211 | 4.56 | 0.000457 |
| *Cxcl5* | NM_009141 | 1.58 | 0.096333 |  | *Tlr9* | NM_031178 | 3.10 | 0.000050 |
| *Cxcl9* | NM_008599 | 9.45 | 0.001736 |  | *Tnf* | NM_013693 | 3.07 | 0.002280 |
| *Cxcr1* | NM_178241 | 0.83 | 0.335001 |  | *Tnfsf14* | NM_019418 | 0.77 | 0.069212 |
| *Cxcr2* | NM_009909 | 0.41 | 0.019978 |  | *Tollip* | NM_023764 | 0.87 | 0.042283 |
| *Cxcr4* | NM_009911 | 1.23 | 0.122045 |  | *Actb* | NM_007393 | 1.18 | 0.053250 |
| *Fasl* | NM_010177 | 1.26 | 0.297384 |  | *B2m* | NM_009735 | 1.73 | 0.004926 |
| *Fos* | NM_010234 | 2.59 | 0.000659 |  | *Gapdh* | NM_008084 | 0.82 | 0.103244 |
| *Ifng* | NM_008337 | 1.23 | 0.705785 |  | *Gusb* | NM_010368 | 1.49 | 0.005001 |
| *Il10* | NM_010548 | 1.70 | 0.044254 |  |  |  |  |  |
| *Il10rb* | NM_008349 | 1.54 | 0.002758 |  |  |  |  |  |

**­Supplementary Table 13. Differential mRNA levels of Snell dwarf mice 10 days post 30°/s protocol vs nonexposed muscles.**

Expression which surpassed 2-fold regulation (below 0.5 fold change or above 2 fold change) with a P value < 0.05 was considered differentially expressed. ND, Not detected. Not highlighted – unchanged, Orange – upregulated, Blue - downregulated. Sample sizes were N = 8-9 per group.
