# Supplementary material for: Transcriptional and morphological responses following distinct muscle contraction protocols for Snell dwarf (Pit1dw/dw ) mice
Source: Physiol Rep. 2024 Sep 3;12(17):e70027. doi: 10.14814/phy2.70027 (PMC11371489; doi:10.14814/phy2.70027)
Supplement: Supplementary file 23 — Table S14. [file PHY2-12-e70027-s024.docx]

|  | RefSeq | 500°/s protocol vs nonexposed | |  |  | RefSeq | 500°/s protocol vs nonexposed | |
| --- | --- | --- | --- | --- | --- | --- | --- | --- |
|  |  |  |  |  |  |  |  |  |
|  |  | Fold change | P value |  |  |  | Fold change | P value |
| *Bcl6* | NM_009744 | 0.63 | 0.049510 |  | *Il17a* | NM_010552 | 0.56 | 0.054920 |
| *C3* | NM_009778 | 0.91 | 0.732182 |  | *Il18* | NM_008360 | 1.93 | 0.005022 |
| *C3ar1* | NM_009779 | 3.42 | 0.001382 |  | *Il1a* | NM_010554 | 0.73 | 0.049618 |
| *C4b* | NM_009780 | 1.29 | 0.333332 |  | *Il1b* | NM_008361 | 0.86 | 0.481459 |
| *Ccl1* | NM_011329 | 0.89 | 0.918383 |  | *Il1r1* | NM_008362 | 0.99 | 0.941195 |
| *Ccl11* | NM_011330 | 0.86 | 0.707407 |  | *Il1rap* | NM_008364 | 0.97 | 0.747697 |
| *Ccl12* | NM_011331 | 3.35 | 0.084674 |  | *Il1rn* | NM_031167 | 0.92 | 0.955148 |
| *Ccl17* | NM_011332 | 0.77 | 0.105939 |  | *Il22* | NM_016971 | 0.60 | 0.166995 |
| *Ccl19* | NM_011888 | 0.91 | 0.307525 |  | *Il23a* | NM_031252 | 0.88 | 0.869758 |
| *Ccl2* | NM_011333 | 0.64 | 0.216871 |  | *Il23r* | NM_144548 | 0.85 | 0.800638 |
| *Ccl20* | NM_016960 | 0.96 | 0.756789 |  | *Il5* | NM_010558 | 0.63 | 0.019267 |
| *Ccl22* | NM_009137 | 0.73 | 0.066762 |  | *Il6* | NM_001314054 | 0.47 | 0.059961 |
| *Ccl24* | NM_019577 | 0.97 | 0.911447 |  | *Il6ra* | NM_010559 | 1.07 | 0.554065 |
| *Ccl25* | NM_009138 | 0.96 | 0.939205 |  | *Il7* | NM_008371 | 0.57 | 0.044269 |
| *Ccl3* | NM_011337 | 1.86 | 0.013821 |  | *Il9* | NM_008373 | 0.57 | 0.052531 |
| *Ccl4* | NM_013652 | 0.66 | 0.237905 |  | *Itgb2* | NM_008404 | 2.58 | 0.000192 |
| *Ccl5* | NM_013653 | 1.83 | 0.009370 |  | *Kng1* | NM_023125 | 0.75 | 0.714966 |
| *Ccl7* | NM_013654 | 1.16 | 0.502400 |  | *Lta* | NM_010735 | ND | ND |
| *Ccl8* | NM_021443 | 12.84 | 0.004650 |  | *Ltb* | NM_008518 | 1.09 | 0.776015 |
| *Ccr1* | NM_009912 | 1.33 | 0.120819 |  | *Ly96* | NM_016923 | 1.43 | 0.010067 |
| *Ccr2* | NM_009915 | 2.06 | 0.004395 |  | *Myd88* | NM_010851 | 1.49 | 0.004431 |
| *Ccr3* | NM_009914 | 4.60 | 0.001105 |  | *Nfkb1* | NM_008689 | 0.87 | 0.696611 |
| *Ccr4* | NM_009916 | 0.57 | 0.004391 |  | *Nos2* | NM_001313921 | 0.73 | 0.466698 |
| *Ccr7* | NM_007719 | 0.88 | 0.336404 |  | *Nr3c1* | NM_008173 | 0.84 | 0.237000 |
| *Cd14* | NM_009841 | 2.01 | 0.003049 |  | *Ptgs2* | NM_011198 | 1.08 | 0.657634 |
| *Cd40* | NM_011611 | 2.57 | 0.002404 |  | *Ripk2* | NM_138952 | 0.71 | 0.669131 |
| *Cd40lg* | NM_011616 | 1.01 | 0.756607 |  | *Sele* | NM_011345 | 0.65 | 0.044177 |
| *Cebpb* | NM_009883 | 0.69 | 0.030036 |  | *Tirap* | NM_054096 | 0.96 | 0.744387 |
| *Crp* | NM_007768 | 1.49 | 0.070243 |  | *Tlr1* | NM_030682 | 3.48 | 0.002200 |
| *Csf1* | NM_007778 | 1.35 | 0.111038 |  | *Tlr2* | NM_011905 | 1.76 | 0.983475 |
| *Cxcl1* | NM_008176 | 1.24 | 0.381524 |  | *Tlr3* | NM_126166 | 1.04 | 0.240600 |
| *Cxcl10* | NM_021274 | 1.32 | 0.233002 |  | *Tlr4* | NM_021297 | 0.98 | 0.921232 |
| *Cxcl11* | NM_019494 | 0.88 | 0.888145 |  | *Tlr5* | NM_016928 | 1.94 | 0.012127 |
| *Cxcl2* | NM_009140 | 0.43 | 0.012011 |  | *Tlr6* | NM_011604 | 1.48 | 0.019748 |
| *Cxcl3* | NM_203320 | 0.61 | 0.070458 |  | *Tlr7* | NM_133211 | 2.81 | 0.009762 |
| *Cxcl5* | NM_009141 | 0.84 | 0.606350 |  | *Tlr9* | NM_031178 | 1.81 | 0.014842 |
| *Cxcl9* | NM_008599 | 5.76 | 0.015065 |  | *Tnf* | NM_013693 | 1.86 | 0.051101 |
| *Cxcr1* | NM_178241 | 0.96 | 0.696651 |  | *Tnfsf14* | NM_019418 | 0.64 | 0.044839 |
| *Cxcr2* | NM_009909 | 0.44 | 0.066029 |  | *Tollip* | NM_023764 | 0.90 | 0.320796 |
| *Cxcr4* | NM_009911 | 0.85 | 0.897208 |  | *Actb* | NM_007393 | 0.99 | 0.612483 |
| *Fasl* | NM_010177 | 1.15 | 0.411889 |  | *B2m* | NM_009735 | 1.33 | 0.060545 |
| *Fos* | NM_010234 | 1.92 | 0.105559 |  | *Gapdh* | NM_008084 | 0.75 | 0.272825 |
| *Ifng* | NM_008337 | 1.36 | 0.599160 |  | *Gusb* | NM_010368 | 1.25 | 0.019160 |
| *Il10* | NM_010548 | 1.39 | 0.156256 |  |  |  |  |  |
| *Il10rb* | NM_008349 | 1.22 | 0.048900 |  |  |  |  |  |

**­Supplementary Table 14. Differential mRNA levels of Snell dwarf mice 10 days post 500°/s protocol vs nonexposed muscles.**

Expression which surpassed 2-fold regulation (below 0.5 fold change or above 2 fold change) with a P value < 0.05 was considered differentially expressed. ND, Not detected. Not highlighted – unchanged, Orange – upregulated, Blue - downregulated. Sample sizes were N = 8 per group.
