# Supplementary material for: Transcriptional and morphological responses following distinct muscle contraction protocols for Snell dwarf (Pit1dw/dw ) mice
Source: Physiol Rep. 2024 Sep 3;12(17):e70027. doi: 10.14814/phy2.70027 (PMC11371489; doi:10.14814/phy2.70027)
Supplement: Supplementary file 24 — Table S15. [file PHY2-12-e70027-s012.docx]

|  | RefSeq | 500°/s protocol vs  30°/s protocol | |  |  | RefSeq | 500°/s protocol vs  30°/s protocol | |
| --- | --- | --- | --- | --- | --- | --- | --- | --- |
|  |  |  |  |  |  |  |  |  |
|  |  | Fold change | P value |  |  |  | Fold change | P value |
| *Bcl6* | NM_009744 | 1.09 | 0.971856 |  | *Il17a* | NM_010552 | 0.89 | 0.920113 |
| *C3* | NM_009778 | 0.86 | 0.545965 |  | *Il18* | NM_008360 | 0.65 | 0.074661 |
| *C3ar1* | NM_009779 | 0.49 | 0.016133 |  | *Il1a* | NM_010554 | 1.06 | 0.736763 |
| *C4b* | NM_009780 | 1.00 | 0.876090 |  | *Il1b* | NM_008361 | 0.66 | 0.128841 |
| *Ccl1* | NM_011329 | 0.84 | 0.937925 |  | *Il1r1* | NM_008362 | 0.95 | 0.699295 |
| *Ccl11* | NM_011330 | 0.87 | 0.725851 |  | *Il1rap* | NM_008364 | 0.81 | 0.116067 |
| *Ccl12* | NM_011331 | 0.71 | 0.774355 |  | *Il1rn* | NM_031167 | 0.61 | 0.240469 |
| *Ccl17* | NM_011332 | 0.70 | 0.025991 |  | *Il22* | NM_016971 | 0.87 | 0.890128 |
| *Ccl19* | NM_011888 | 0.96 | 0.510855 |  | *Il23a* | NM_031252 | 0.75 | 0.248036 |
| *Ccl2* | NM_011333 | 0.77 | 0.533399 |  | *Il23r* | NM_144548 | 1.22 | 0.301360 |
| *Ccl20* | NM_016960 | 1.51 | 0.141814 |  | *Il5* | NM_010558 | 0.78 | 0.267281 |
| *Ccl22* | NM_009137 | 0.94 | 0.296556 |  | *Il6* | NM_001314054 | 0.37 | 0.005260 |
| *Ccl24* | NM_019577 | 1.08 | 0.659310 |  | *Il6ra* | NM_010559 | 0.80 | 0.127610 |
| *Ccl25* | NM_009138 | 0.89 | 0.469903 |  | *Il7* | NM_008371 | 0.62 | 0.082915 |
| *Ccl3* | NM_011337 | 0.71 | 0.181564 |  | *Il9* | NM_008373 | 0.99 | 0.731514 |
| *Ccl4* | NM_013652 | 0.67 | 0.236853 |  | *Itgb2* | NM_008404 | 0.64 | 0.090740 |
| *Ccl5* | NM_013653 | 0.45 | 0.002904 |  | *Kng1* | NM_023125 | 1.14 | 0.451686 |
| *Ccl7* | NM_013654 | 0.61 | 0.187920 |  | *Lta* | NM_010735 | ND | ND |
| *Ccl8* | NM_021443 | 0.56 | 0.282179 |  | *Ltb* | NM_008518 | 0.85 | 0.398164 |
| *Ccr1* | NM_009912 | 0.93 | 0.907008 |  | *Ly96* | NM_016923 | 0.84 | 0.224400 |
| *Ccr2* | NM_009915 | 0.56 | 0.129460 |  | *Myd88* | NM_010851 | 0.83 | 0.197132 |
| *Ccr3* | NM_009914 | 0.46 | 0.074570 |  | *Nfkb1* | NM_008689 | 0.79 | 0.246946 |
| *Ccr4* | NM_009916 | 0.95 | 0.809188 |  | *Nos2* | NM_001313921 | 0.74 | 0.581740 |
| *Ccr7* | NM_007719 | 0.70 | 0.102486 |  | *Nr3c1* | NM_008173 | 0.85 | 0.297335 |
| *Cd14* | NM_009841 | 0.51 | 0.091926 |  | *Ptgs2* | NM_011198 | 0.82 | 0.400923 |
| *Cd40* | NM_011611 | 0.89 | 0.425676 |  | *Ripk2* | NM_138952 | 0.66 | 0.328051 |
| *Cd40lg* | NM_011616 | 1.19 | 0.512986 |  | *Sele* | NM_011345 | 1.01 | 0.923100 |
| *Cebpb* | NM_009883 | 1.01 | 0.766341 |  | *Tirap* | NM_054096 | 1.17 | 0.373033 |
| *Crp* | NM_007768 | 0.91 | 0.534500 |  | *Tlr1* | NM_030682 | 0.86 | 0.208472 |
| *Csf1* | NM_007778 | 0.90 | 0.723760 |  | *Tlr2* | NM_011905 | 0.56 | 0.005316 |
| *Cxcl1* | NM_008176 | 0.80 | 0.470471 |  | *Tlr3* | NM_126166 | 0.61 | 0.236314 |
| *Cxcl10* | NM_021274 | 0.89 | 0.791846 |  | *Tlr4* | NM_021297 | 0.65 | 0.026354 |
| *Cxcl11* | NM_019494 | 0.76 | 0.308464 |  | *Tlr5* | NM_016928 | 0.50 | 0.053011 |
| *Cxcl2* | NM_009140 | 0.83 | 0.871188 |  | *Tlr6* | NM_011604 | 0.63 | 0.063977 |
| *Cxcl3* | NM_203320 | 0.76 | 0.431772 |  | *Tlr7* | NM_133211 | 0.62 | 0.135915 |
| *Cxcl5* | NM_009141 | 0.53 | 0.057682 |  | *Tlr9* | NM_031178 | 0.58 | 0.050157 |
| *Cxcl9* | NM_008599 | 0.61 | 0.200628 |  | *Tnf* | NM_013693 | 0.60 | 0.091437 |
| *Cxcr1* | NM_178241 | 1.15 | 0.448949 |  | *Tnfsf14* | NM_019418 | 0.83 | 0.484750 |
| *Cxcr2* | NM_009909 | 1.07 | 0.557680 |  | *Tollip* | NM_023764 | 1.04 | 0.540370 |
| *Cxcr4* | NM_009911 | 0.69 | 0.147143 |  | *Actb* | NM_007393 | 0.84 | 0.454489 |
| *Fasl* | NM_010177 | 0.91 | 0.872694 |  | *B2m* | NM_009735 | 0.77 | 0.252218 |
| *Fos* | NM_010234 | 0.74 | 0.666836 |  | *Gapdh* | NM_008084 | 0.91 | 0.961306 |
| *Ifng* | NM_008337 | 1.10 | 0.854482 |  | *Gusb* | NM_010368 | 0.84 | 0.138843 |
| *Il10* | NM_010548 | 0.82 | 0.820313 |  |  |  |  |  |
| *Il10rb* | NM_008349 | 0.80 | 0.100436 |  |  |  |  |  |

**­Supplementary Table 15. Differential mRNA levels of muscles of Snell dwarf mice 10 days post 500°/s protocol vs 30°/s protocol.**

Expression which surpassed 2-fold regulation (below 0.5 fold change or above 2 fold change) with a P value < 0.05 was considered differentially expressed. ND, Not detected. Not highlighted – unchanged, Orange – upregulated, Blue - downregulated. Sample sizes were N = 8-9 per group.
